# Interplay of ferroptotic and apoptotic cell death and its modulation by BH3-mimetics

**DOI:** 10.1038/s41418-025-01514-7

**Published:** 2025-04-29

**Authors:** Yun Qiu, Juliana A. Hüther, Bianca Wank, Antonia Rath, René Tykwe, Maceler Aldrovandi, Bernhard Henkelmann, Julia Mergner, Toshitaka Nakamura, Sabine Laschat, Marcus Conrad, Daniela Stöhr, Markus Rehm

**Affiliations:** 1 Institute of Cell Biology and Immunology, University of Stuttgart, Stuttgart, Germany; 2 Institute of Organic Chemistry, University of Stuttgart, Stuttgart, Germany; 3 Institute of Metabolism and Cell Death, Molecular Targets and Therapeutics Center, Helmholtz Zentrum Munich, Neuherberg, Germany; 4 Bavarian Center for Biomolecular Mass Spectrometry at MRI, TUM, Munich, Germany; 5 Stuttgart Research Center Systems Biology, University of Stuttgart, Stuttgart, Germany

**Keywords:** Cell biology, Biochemistry

## Abstract

Ferroptosis and apoptosis are widely considered to be independent cell death modalities. Ferroptotic cell death is a consequence of insufficient radical detoxification and progressive lipid peroxidation, which is counteracted by glutathione peroxidase-4 (GPX4). Apoptotic cell death can be triggered by a wide variety of stresses, including oxygen radicals, and can be suppressed by anti-apoptotic members of the BCL-2 protein family. Mitochondria are the main interaction site of BCL-2 family members and likewise a major source of oxygen radical stress. We therefore studied if ferroptosis and apoptosis might intersect and possibly interfere with one another. Indeed, cells dying from impaired GPX4 activity displayed hallmarks of both ferroptotic and apoptotic cell death, with the latter including (transient) membrane blebbing, submaximal cytochrome-c release and caspase activation. Targeting BCL-2, MCL-1 or BCL-XL with BH3-mimetics under conditions of moderate ferroptotic stress in many cases synergistically enhanced overall cell death and frequently skewed primarily ferroptotic into apoptotic outcomes. Surprisingly though, in other cases BH3-mimetics, most notably the BCL-XL inhibitor WEHI-539, counter-intuitively suppressed cell death and promoted cell survival following GPX4 inhibition. Further studies revealed that most BH3-mimetics possess previously undescribed antioxidant activities that counteract ferroptotic cell death at commonly employed concentration ranges. Our results therefore show that ferroptosis and apoptosis can intersect. We also show that combining ferroptotic stress with BH3-mimetics, context-dependently can either enhance and convert cell death outcomes between ferroptosis and apoptosis or can also suppress cell death by intrinsic antioxidant activities.

## Introduction

Ferroptosis and apoptosis are widely considered to be independent cell death modalities. Ferroptotic death results from excessive lipid peroxidation, which manifests when the cellular antioxidant systems are overwhelmed in their capacity to reduce (phospho)lipid hydroperoxides to their respective alcohols. In this process, the glutathione-dependent antioxidant system plays a key role with glutathione peroxidase-4 (GPX4) efficiently neutralizing phospholipid hydroperoxides at the expense of reduced glutathione (GSH). Cellular GSH levels are maintained by the activity of glutathione reductase or by de novo synthesis [1]. For GSH synthesis, many cells rely on sufficient uptake of extracellular cystine, the oxidised and dimeric form of cysteine, through the cystine-glutamate antiporter system x_c_^−^ [2]. Indeed, cystine was identified as an essential component of common cell culture media already in the early days when developing tissue/cell culture systems [3]. Both GPX4 and system x_c_^−^ are pharmacological targets of synthetic ferroptosis-inducing agents, with RSL3 and erastin having been major hits in screens for compounds toxic to otherwise highly treatment resistant RAS-mutated cancer cells [4, 5]. While RSL3 directly inhibits GPX4 by covalent interaction with the active site selenocysteine and was the lead for the development of related GPX4 inhibitors, erastin indirectly promotes ferroptosis by inhibiting system x_c_^−^ and cystine uptake, causing cellular GSH deprivation and GPX4 inactivation [6, 7]. Ultimately, the membrane damage arising from widespread lipid peroxidation is thought to result in cell swelling and eventually cell rupture, whereby cellular content is released into the extracellular space. Whether specific molecular mechanisms are involved in the orchestration of this final stage of cell rupture during ferroptotic death is still being studied, with conflicting conclusions on the role of ninjurin-1 for plasma membrane permeabilisation [8, 9].

In contrast to ferroptotic cell death, the primary endpoint of apoptosis is not cell swelling and lysis, but cell condensation and dismantling into membrane-enclosed apoptotic bodies or “blebs” [10]. Apoptosis is an active cellular response to external and internal stress conditions, which stands in contrast to ferroptosis as a consequence of perturbing steady-state cellular metabolism and redox fitness. In most scenarios of apoptotic cell death, mitochondrial outer membrane permeabilisation (MOMP) is a key decision point for triggering the execution of this form of cell death. MOMP is controlled by the BCL-2 protein family, with BAX and BAK constituting the major pore forming proteins and BCL-2, MCL-1 and BCL-XL acting as their antagonists [11]. A third group of BCL-2 family members are BH3-only proteins, of which the sensitizing BH3-only proteins act as neutralizers of BCL-2, MCL-1 and BCL-XL, thereby allowing BAX and/or BAK activation and oligomerisation to proceed. BH3-mimetics, small molecules that emulate the function of sensitizer BH3-only proteins, are now widely used in experimental settings to pharmacologically trigger MOMP [11, 12]. As a BCL-2 inhibitor, venetoclax has been approved for clinical use in a number of leukemias, while optimal translational avenues for inhibitors of MCL-1 and BCL-XL in single or combination treatments are currently being evaluated [11, 12].

Mitochondria are a major site of membrane-associated redox signalling and likewise a source of cellular oxidative stress that could lead to ferroptotic cell death. At the same time, oxidative stress can sensitise to apoptosis [13], where mitochondria are the central hub for the integration of apoptotic stress signals prior to triggering apoptosis execution. We therefore set out to study if both ferroptosis and apoptosis could possibly be coupled in response to GPX4 inhibition, and also if BH3-mimetics might modify cellular fates between survival and ferroptosis or apoptosis outcomes.

## Materials and methods

### Reagents and antibodies

S63845 and Z-VAD-FMK (zVAD) were purchased from APExBIO Technology (Houston, TX, USA). ABT-199 was from Active Biochemicals Co. Limited (Hong Kong, China). A-1331852 was from TargetMol Chemicals Inc. (Boston, MA, USA), WEHI-539 hydrochloride was from MedChemExpress (Princeton, NJ, USA). Erastin was from Merck Chemicals GmbH (#329600, Darmstadt, Germany). DMSO, bovine serum albumin (BSA) and Sodium deoxycholate were from Carl Roth (Karlsruhe, Germany). Q-VD-Oph (QVD), 4-hydroxytamoxifen and AZD5991 were from Selleckchem (Houston, TX, USA). Roswell Park Memorial Institute (RPMI) 1460 medium, Dulbecco’s Modified Eagle (DMEM) medium, PBS without Mg²⁺/Ca²⁺ and sodium pyruvate were purchased from Thermo Fisher Scientific (Gibco, Waltham, MA, USA). Lipofectamine™ RNAiMAX transfection reagent, Hoechst 33342, C11 BODIPY 581/591, and MitoTracker CMXRos were purchased from Invitrogen (Carlsbad, CA, USA). Ferrostatin-1 (FER), (1S, 3 R)-RSL3 (RSL3), glutathione reductase, fetal bovine serum, and propidium iodide (PI) were purchased from Sigma-Aldrich (Munich, Germany). Western blocking reagent, cOmplete protease inhibitor cocktail, PhosSTOP phosphatase inhibitor cocktail were from Roche Diagnostics (Mannheim, Germany). Paraformaldehyde was from Santa Cruz Biotechnology (Dallas, Texas, USA). STY-BODIPY were from Cayman Chemical Company (Ann Arbour, Michigan, USA). Fluoromount G was from Southern Biotechnology Associates Inc (Birmingham, Alabama, USA). 2,2’-Azobis(2-amidinopropane) dihydrochloride was from Sigma-Aldrich (St. Louis, Missouri, USA). Phosphatidylcholine (chicken egg) was purchased from Lipoid (Ludwigshafen am Rhein, Rheinland-Pfalz, Germany). Ammonium acetate for LC-MS LiChropur™, dichloromethane, diethylenetriaminepentaacetic acid, butylated hydroxytoluene, L-alpha-phosphatidylcholine from soybean, reduced glutathione and glutathione reductase from *Saccharomyces cerevisiae* were purchased from Sigma-Aldrich (St. Louis, Missouri, USA). SPLASH® LIPIDOMIX® and UltimateSPLASH® ONE Mass Spec Standard were from Avanti Polar Lipids Inc (Alabaster, USA). Water, methanol, acetonitrile and 2-propanol were from CHEMSOLUTE® (Renningen, Germany). Chloroform, ammonium formate (NH_4_HCO_2_) and formic acid were from Honeywell Riedel-de Haën (Seelze, Germany). All antibodies used in immunoblotting were as follows: mouse monoclonal α-tubulin (1:5000, #3873), rabbit polyclonal β-Actin (1:1000, #4967), rabbit polyclonal caspase-3 (1:1000, #9662), mouse monoclonal BCL-2 (1:1000, #15071), rabbit monoclonal BCL-XL (1:1000, #2764), rabbit monoclonal MCL-1 (1:1000, #94296), rabbit polyclonal BAX (1:1000, #2772), rabbit polyclonal BAK (1:1000, #3814) and rabbit monoclonal Tom20 (1:1000, #42406) all from Cell Signaling Technology (Danvers, MA, USA). Rabbit monoclonal GPX4 (1:1000, ab125066) from Abcam (Cambridge, UK), mouse monoclonal PARP (1:1000, #556494, clone 4C10-5) and mouse monoclonal cytochrome-c (1:1000, #556433) from BD Pharmingen (Heidelberg, Germany). Goat anti-mouse IgG HRP-linked antibody (#115-035-062) and goat anti-rabbit IgG HRP-linked antibody (#111-035-144) were purchased from Dianova (Hamburg, Germany). Goat anti-rabbit IRDye680CW and IRDye800CW (1:10000; #926-32211, #926-68071) were purchased from LI-COR Biosciences (Bad Homburg, Germany). The following antibodies were used in immunofluorescence staining: mouse monoclonal cytochrome-c (1:100) from Invitrogen (Carlsbad, CA, USA). Goat anti-rabbit IgG (H + L) Alexa Fluor 488 (#A-11008) and goat anti-mouse IgG (H + L) Alexa Fluor 647 (#A-21235) were from Invitrogen (Carlsbad, CA, USA).

### Cell culture

4-Hydroxytamoxifen-inducible *Gpx4*^*−/−*^ immortalised mouse embryonic fibroblasts (Pfa1 cells) and HT1080_M_ *Gpx4*^*−/−*^ cells used in this study were described previously [14, 15]. All other commercially available cell lines were used as authenticated materials (Microsynth, Göttingen, Germany). HT1080_S_ and HT1080_M_ are two strains of the HT1080 cell line that differ in their sensitivity towards RSL3 (Supplemental Fig. 1A). Both are genetically authenticated as 100% HT1080, exhibit highly similar proteome profiles (Supplemental Fig. 1B) and are assumed to have arisen from biological cell line variability and plasticity [16]. All cell lines were grown in RPMI 1460 medium supplemented with 10% fetal bovine serum except HT-22 cells which were cultivated in DMEM supplemented with 10% fetal bovine serum and 1 mM sodium pyruvate. HT1080_M_ *GPX4*^*−/−*^ cells were cultured in the presence of 2 µM FER. All cell lines were tested negative for mycoplasma contamination.

### Cell death assessment

Cells were seeded 24 h prior to stimulation. PI (1 µM) was used as a fluorescent indicator for cell death. Cells were pre-incubated with QVD for 30 min before the addition of the other reagents, where applicable. Cells were monitored in an IncuCyte S3 (Sartorius, Göttingen, Germany) and cell death was quantified using the IncuCyte analysis software, utilizing the cell-by-cell analysis module.

### Immunoblotting

Immunoblotting was performed as previously described [17]. Cells were harvested by trypsinization and subsequently washed with cold phosphate-buffered saline (PBS, 2.67 mM KCl, 1.47 mM KH_2_PO_4_, 137.9 mM NaCl, 8.6 mM Na_2_HPO_4_, pH 7.4). Cells were centrifuged at 300 *g* for 5 min, with the subsequent removal of supernatants. Lysis buffer (150 mM NaCl, 1 mM EDTA, 20 mM TRIS, 1% (v/v) Triton X-100, pH 7.6) supplemented with 1× cOmplete protease inhibitor cocktail was added and the samples were incubated for 15 min on ice. Following centrifugation at 16,100 *g* for 15 min at 4˚C, protein concentrations were determined by Bradford assay. Thereafter, 5 x Laemmli buffer (500 mM DTT, 10% (w/v) SDS, 25% (v/v) glycerol, 0.05% (w/v) bromophenol blue, 312.5 mM Tris-HCl) was added and the samples were incubated at 95°C for 5 min. Protein samples were separated using 4-12% Bis-Tris mini protein gels and transferred to a nitrocellulose membrane using an iBlot2 gel transfer device (Thermo Fisher Scientific, Waltham, MA, USA). Thereafter, the membrane was blocked by using a western blocking reagent for 1 h, and subsequently incubated with the primary and secondary antibodies. Signals were acquired with an ECL imager (Amersham^TM^ Imager 600, GE Healthcare Life Sciences, Chicago, IL, USA). Luminescence was detected at a depth of 12-bit in the linear detection range. Alternatively, images were acquired with a LICOR Odyssey Imager (Lincoln, NA, USA). For presentation, images were contrast-adjusted with ImageJ (National Institute of Health, USA, http://rsb.info.nih.gov/ij) and converted to 8 bit.

### Cell fractionation

Cells were cultured in 6-well plates one day prior to stimulation. On the following day, after the designated stimulation period, the medium was aspirated. Then, 150 μl of permeabilisation buffer (210 mM D-Mannitol, 70 mM D-(+)-Sucrose, 10 mM HEPES, 5 mM Succinate, 0.5 mM EGTA, 100 µg/ml Digitonin, pH 7.2) were added to the centre of each well. The plate was gently rocked from side to side and from top to bottom for 45 seconds to ensure even distribution of the permeabilisation buffer. The supernatant from each well was collected and stored on ice (cytosolic fraction). Subsequently, 150 μl of lysis buffer containing 1x cOmplete protease inhibitor was added to the wells and pellet fractions were collected. Both fractions were then centrifuged at 13,000 *g* for 10 min at 4°C. The resulting supernatants were retained and protein concentrations were determined using the DC Protein Assay Kit II (Bio-Rad, Feldkirchen, Germany).

### Gene silencing with siRNA

Cells were seeded in 6-well plates and transfected 24 h later. For that, Lipofectamine® RNAiMAX Reagent and siRNA were diluted in Opti-MEM® medium according to the manufacturer’s instructions (Thermo Fisher Scientific, Waltham, MA, USA). The diluted siRNAs (10 nM) and Lipofectamine® RNAiMAX Reagent (10 nM) were mixed at a ratio of 1:1, and incubated at room temperature (RT) for 5 min. Thereafter, the solution was added drop by drop to each well. After 72 h, the transfection reagent was aspirated.

### C11 BODIPY 581/591 measurements

Cells were seeded one day prior to stimulation. The treatments were followed by incubation with C11 BODIPY 581/591 (1.5 µM) for 30 min at 37˚C in the dark. Cells were harvested by trypsinisation, and centrifuged at 300 *g* for 5 min at RT. The cell pellet was resuspended in 100 µl PBS. For flow cytometric analysis, at least 10,000 events were acquired per sample using one of two flow cytometers: either a flow cytometer (CytoFLEX and CytExpert 2.4, Beckman Coulter), which utilizes a 488-nm laser paired with a 530/30 nm bandpass filter, or a flow cytometry (MACSQuant Analyzer 10, Miltenyi Biotec, Bergisch Gladbach, Germany), which is equipped with a 488-nm laser and a 525/50 nm bandpass filter for detection. Data from all experiments were analysed using the FlowJo software (Treestar).

### Lipid extraction

Pfa1 cells were seeded in 10 cm petri dishes (4 ×10^5^ cells/dish). On the next day, cells were treated with RSL3, in the presence or absence of WEHI-539. Four hours later, cellular lipids were extracted using the Folch method [18]. Briefly, cells were washed with antioxidant buffer (100 µM diethylenetriamine pentaacetate and 100 µM butylated hydroxytoluene in PBS, pH 7.4), and harvested by trypsinisation. Cells were centrifuged at 1500 rpm for 5 min at 4°C, cell pellets were washed with antioxidant buffer and centrifuged. The supernatant was removed, and cell pellets were resuspended in 100 µl antioxidant buffer. Cell pellets were then transferred into glass tubes containing ice-cold methanol (1.5 ml), chloroform (3 ml) and internal standards (SPLASH^®^ LIPIDOMIX^®^ (0.5 μl) and UltimateSPLASH® ONE (5 μl)). Samples were vortexed and incubated for 1 h at 4°C on a roller shaker. Phase separation was induced by addition of water (1.25 ml), followed by vortexing, incubation for 10 min at 4°C on a roller shaker and centrifugation (10 min, 4°C, 600 *g*). The lower layer was collected, and the sample re-extracted. Lipid extracts were dried in a vacuum evaporator, dissolved in 120 µL of isopropanol and stored in −80°C before analysis by LC/MS/MS. All solvents were spiked with 0.01% (w/v) BHT and cooled on ice prior lipid extraction.

### Mass spectrometry analysis of PE, PC, LPE and LPC lipids

Hydrophilic interaction chromatography (HILIC) was carried out on a SCIEX Triple Quad™ 7500 System equipped with a Luna® NH2 column (100 ×2 mm; 3 µm, 100 Å, Phenomenex). Lipids were separated by gradient elution with solvent A (acetonitrile/dichloromethane, 93:7, v/v) and B (acetonitrile/water, 50:50, v/v) both containing 5 mM ammonium acetate. Mobile phase B was adjusted to pH 8.2 with NH_3_ (15%). Separation was performed at 35°C using following gradient: 0-2 min – 0% B isocratic (flow 0.2 ml/min), 2-11 min – 0 to 40% B (flow 0.5 ml/min), 11-11.5 min – 40 to 70% B (flow 0.5 ml/min), 11.5-12.5 min – 70 to 100% B (flow 0.5 ml/min), 12.5-15 min – 100% B isocratic (flow 0.5 ml/min), 15-15.1 min – 100 to 0% B followed by 2.5 min re-equilibration at 0% B. The triple quadrupole mass spectrometer was operated with the following settings: TEM 500°C, GS1 45, GS2 70, CUR 45, CAD 9, IS − 3500 V. Data was acquired in multiple reaction monitoring (MRM) mode. For quantification, the area under the curve for the precursor mass to fragment mass was integrated, using SCIEX OS software, and normalized by appropriate lipid species from UltimateSPLASH® ONE Mass Spec Standard (Avanti), e.g. by PC(17:0_22:4(d5)), PE(17:0_18:1((d5), LPC(17:0(d5)) and LPE(17:0((d5). Isotopic correction was performed using an application developed specifically for targeted data acquired with class-based chromatographic separations in MRM mode [19]. The application is available at https://slinghub.shinyapps.io/LICAR/.

### In silico oxidation

Following phospholipid quantification in Pfa1 cells, the most abundant PUFA-containing PCs (25 molecular species) and PEs (31 molecular species) (Supplemental Table 1) were used for in silico oxidation by LPPtiger 2 software [20]. The oxidation was performed considering a maximum of 2 sites and addition of 3 oxygens. The list of modifications included hydroperoxy, hydroxy, epoxy, and keto groups and truncated products. Elemental composition of predicted oxidized PCs and PEs was used to compose inclusion lists used for MS analysis.

### Mass spectrometry analysis of oxidized PCs and PEs

Reversed phase liquid chromatography (RPLC) was carried out on a Shimadzu ExionLC equipped with an Accucore^TM^ C18 column (150 ×2.1 mm; 2.6 µm, 80 Å, Thermo Fisher Scientific). Oxidized lipids were separated by gradient elution with solvent A (acetonitrile/water, 1:1, v/v) and B (isopropanol/acetonitrile/water, 85:10:5, v/v) both containing 5 mM NH_4_HCO_2_ and 0.1% (v/v) formic acid. Separation was performed at 50°C with a flow rate of 0.3 ml/min using following gradient: 0-20 min – 10 to 86% B (curve 4), 20-22 min – 86 to 95% B, 22-26 min – 95% isocratic, 26-26.1 min – 95 to 10% B followed by 5 min re-equilibration at 10% B [21]. Mass spectrometry analysis was performed on a e SCIEX Triple Quad™ 7500 System equipped with an ESI source and operated in negative ion mode, followed by data analysis using SCIEX OS software. Data was acquired in MRM mode, monitoring transitions from the precursor ion to fragment ion (Supplemental Table 2). The mass spectrometer was operated with the following parameters: TEM 500°C, GS1 40, GS2 70, CUR 45, CAD 9, IS − 3000 V. The area under the curve for the precursor mass to fragment mass was integrated and normalized by appropriate lipid species from SPLASH^®^ LIPIDOMIX^®^ (Avanti), e.g. by LPE(18:1(d7)) or PE(15:0/18:1(d7)). For identification, product ion spectra were obtained at the apex of the MRM transitions, with the MS operating in ion trap mode and compared to in silico MS/MS libraries generated by LPPtiger 2 software.

### Data analysis and visualization

Quantified normalized peak areas were log-transformed and autoscaled in MetaboAnalyst v.6 online platform v6.0 (https://www.metaboanalyst.ca, Xia Lab, McGill University, Montreal, Canada). Zero values were replaced by 0.2× the minimum values detected for a given oxidized lipid within the samples. Oxidized lipid species showing a significant difference (ANOVA, adjusted P-value (false discovery rate (FDR)) cutoff: 0.05) between samples were used for the heat maps. Data processed by MetaboAnalyst were exported as CSV files and used for preparing heat maps in Genesis v.1.8.1 (Thallinger Lab, Graz University of Technology, Graz, Austria). Samples were clustered by average linkage weighted pair group method with arithmetic mean (WPGMA) agglomeration rule.

### Determination of GPX4 activity

Cells were seeded in 10 cm petri dishes and treated 24 h later with RSL3 in the presence or absence of WEHI-539 or FER for the indicated time points. The cells were washed with PBS, harvested, and then lysed in 100 µl of extraction buffer (1 mM EDTA, 150 mM KCl, 2 mM β-mercaptoethanol, 0.1% (w/v) CHAPS, 100 mM KH_2_PO_4_/K_2_HPO_4_, pH 7.4). Cell lysates were subsequently centrifuged at 17,000 *g* and 4°C for 2 min. Then, 50 µl of protein supernatant was added to 1 ml reaction buffer (100 mM KH_2_PO_4_/K_2_HPO_4_, 5 mM EDTA, 5 mM glutathione, 0.1% peroxide-free Triton X-100, and 0.2 mM fresh NADPH). For the blank, 1 µl glutathione reductase was added in addition. Finally, 20 µl L-alpha-phosphatidylcholine hydroperoxide solution (25 µM) was added to initiate the reaction. GPX4 activity was measured by the absorbance decrease of NADPH at 340 nm.

For normalisation, the protein concentration of the samples was measured by Pierce™ 660 nm Protein Assay Kit using BSA as standard (Thermo Fisher Scientific, Waltham, MA, USA). The measurements were performed in a SpectraMax microplate reader (Molecular Device GmbH). The results were normalised to that of the DMSO control.

### FENIX assay

Egg phosphatidylcholine was weighed in a glass vial and dissolved in a minimal volume of chloroform. The solvent was evaporated under nitrogen, resulting in a thin film on the vial wall. The film was hydrated with PBS solution to produce a 20 mM lipid suspension. This lipid suspension underwent 10-15 freeze–thaw–sonication cycles, with each cycle involving freezing the vial in liquid nitrogen and thawing in a 37°C water bath. Finally, the lipid suspension was extruded 20–25 times using a mini extruder equipped with a 100 nm polycarbonate membrane (Avanti Polar Lipids, Alabaster, USA). Liposomes (from the above suspension) and STY-BODIPY (a 3 mM stock in DMSO) were diluted by PBS, resulting in the mastermix. The test compounds (2 µl) were added to a 96-well plate, followed by the addition of 295 µl mastermix. The plate was incubated for 10 min at 37°C in a plate reader. Subsequently, the plate was ejected from the plate reader, and 3 µl of 2,2’-Azobis (2-amidinopropane) dihydrochloride (AAPH, 1 mM) was added, followed by mixing for 5 min, resulting in final concentrations of 1 mM liposomes, 1 µM STY-BODIPY, 1 mM AAPH and the test compounds at the indicated concentrations. Fluorescence (λ_ex_ = 488 nm, λ_em_ = 518 nm) was recorded every 10 min for the duration of the experiment. Data were transformed using the response factor of STY-BODIPY, followed by subtraction from the values of the DMSO control group in the absence of the radical initiator. Data were further analysed using GraphPad Prism 8.0.

### Immunofluorescence staining

Cells were seeded on sterilised glass coverslips one day prior to the experiment. MitoTracker CMXRos (200 nM) was added 30 min before the end of stimulation times. Afterwards, cells were washed two times with PBS and fixed with 4% paraformaldehyde (in PBS) for 10 min in the dark. Permeabilisation of the cells was carried out after three more washing steps with PBS using 0.1% (v/v) Triton X-100 (in PBS) for 10 min at RT and protected from light. Cells were washed once with PBS before unspecific binding sites were blocked with 4% BSA (in PBS) for 30 min at RT in the dark. After blocking, cells were incubated with the respective primary antibodies at RT for 1 h in the dark. Subsequently, cells were washed once with PBS and incubated with the respective fluorophore-labeled antibodies for 45 min at RT and protected from light. After incubation, cell nuclei were stained with Hoechst 33342 (1 µg/ml) for 10 min at RT in the dark. Subsequently, cells were washed three times with PBS before the coverslips were mounted on microscopy slides using Fluoromount G. Images of the probes were taken using a confocal microscope (Zeiss LSM710, Carl Zeiss, Jena, Germany), with an 40x/1.30 oil objective. Images were analysed using the CellProfiler software [22]. Masks were generated for cytoplasm and mitochondria and staining intensities were measured.

### Lactate dehydrogenase (LDH) assay

At specified time points, 100 μl of cell culture supernatant was collected. The remaining cells were lysed with 100 μl of 0.1% TritonX-100 solution in PBS. To determine the background signal, 100 μl of either cell culture media or 0.1% Triton X-100 solution in PBS was used. Subsequently, 100 μl of reaction master mix consisting of the components of the LDH reagent kit (Takara Bio Inc., Shiga, Otsu, Japan) (reagent A and reagent B in a ratio of 1:45) was added to each sample and incubated for a period of 15 to 30 min. The absorbance of the samples was measured at 492 nm by spectrophotometry. The results were normalised to the DMSO control.

### Live cell imaging

Cells were seeded one day prior to treatment in a four-compartment glass bottom dish. After stimulation with RSL3 in the presence or absence of QVD and/or FER (pre-incubation for 30 min), cells were imaged every 10 min for a total duration of 24 h using a Zeiss Cell Observer microscope (20x/0.80, Axio Observer.Z1/7, Carl Zeiss, Jena, Germany). Analysis of the cell morphology and cell fate was done manually in the ZEN software.

### Cell death assessment using flow cytometry

Cells were seeded in 12-well plates one day prior to treatment. Cells were then stimulated with RSL3, in the presence of FER or WEHI-539. After the indicated times, cells were harvested by trypsinisation and centrifuged at 300 *g* for 5 min at RT. Cell pellets were resuspended in 100 µl PI solution (2 µg/ml in PBS) and incubated for 10 min at 37°C. The cell suspension was analysed using a flow cytometer (CytoFLEX and CytExpert 2.4, Beckman Coulter), and at least 10,000 events were analysed per sample. Data were analysed using FlowJo software (Treestar).

### Calculation of the coefficient of drug interaction (CDI)

CDIs were determined according to the Webb´s fractional product [23]. CDI = E(AB)/(E(A) * E(B)), where E(A), E(B), and E(AB) represent the percentages of surviving cells after treatment with compound A, compound B, and the combination of A and B, respectively, relative to the control groups. CDI ≤ 0.9 indicates synergism, while CDI > 1.1 indicates antagonism.

### Long-term survival assay

Cells were subjected to continuous treatment with WEHI-539 either in the presence or absence of RSL3 over a 7-day period. Cells were split when cell densities reached 80–100%. Supernatants were replaced every two days. Cells were monitored and analysed using an IncuCyte S3 station, utilizing the cell-by-cell analysis module.

### Electrochemical methods

Differential pulse voltammetry (DPV) experiments were performed at RT and under Argon atmosphere. An AutolabPGSTAT204 potentiostat (Metrohm Autolab, Utrecht, Netherlands) was used for all measurements. The experiment setup contained a three-electrode glass cell equipped with an AgCl-coated silver wire as pseudo reference electrode (RE), a Pt plate as counter electrode (CE) and a working electrode (WE) Au (vacuum deposited on glass slides over 3 nm of adhesion Cr layer; 30 nm Au layer) on S ~ 0.5 cm^2^ slides. For all electrochemical measurements a 0.1 M solution of the supporting electrolyte Bu_4_NPF_6_ (Sigma Aldrich, electrochemical grade) in CH_2_Cl_2_ (Sigma Aldrich, HPLC grade, dry) was employed as received. All substrates were measured in a concentration of 0.3 mM in 0.1 M CH_2_Cl_2_ / Bu_4_NPF_6_ with a scan rate of 0.2 Vs-1 from 0 V to 2 V for the oxidation and from 0 V to -2 V for the reduction. The experiment solution was degassed by Argon before the measurements. The potentials were referenced to the formal potential of the Fc | Fc^+^ reference redox couple, measured in the same conditions as the analytes.

### Proteomics

For each cell line, two biological replicates (B1 and B2) were obtained from separate 10 cm dishes harvested on different days. Immediately before lysis, SDC lysis buffer was freshly prepared, consisting of 4% sodium deoxycholate in 100 mM Tris-HCl (pH 8.5), supplemented with 1× complete EDTA-free protease inhibitor cocktail and 1× PhosSTOP phosphatase inhibitor cocktail. Cell dishes were washed twice with room temperature PBS without Mg²⁺/Ca²⁺. Dishes were then placed on ice and SDC lysis buffer was added. After 5 min incubation on ice, the lysate was collected with cell scrapers. The lysate was then boiled at 95°C for 5 min while shaking at 800 rpm and stored at −80°C until further use.

Protein concentrations were measured by BCA assay according to the manufacturer’s instructions. Two separate sample digests were performed to generate two technical replicates for each cell line sample (B1, B2), in-solution digestion (T1) and SP3-digestion (T2). In-solution: lysate containing 200 μg protein was reduced and alkylated with 10 mM TCEP/40 mM CAA for 5 min at 45°C and samples digested with Trypsin [2×1:100 (w/w) enzyme-to-protein ratio, Pierce] overnight. After digestion, samples were acidified with formic acid (FA), centrifuged at 20,000 *g* for 10 min to pellet the SDC precipitation, and the supernatant was desalted on C18 SepPAC50 columns (WATERS). Peptides were eluted with 0.1% FA in 50% ACN and vacuum dried. SP3: the SDC detergent was removed from lysates by SP3 cleanup on an Agilent BRAVO system (Agilent Technologies). Briefly, lysate containing 200 μg of protein was mixed with SP3 beads, and proteins were precipitated onto a 50:50 mixture of Sera-Mag Speed Bead types A and B (Thermo Fisher Scientific) in 70% acetonitrile. Beads were washed two times with 80% ethanol in water and once with acetonitrile. Bead-bound proteins were reduced in 100 μl with 10 mM DTT, 100 mM Ammoniumbicarbonate followed by alkylation of cysteines with 55 mM chloroacetamide. The tryptic digest was performed overnight with a 1:50 enzyme-to-protein ratio. On the next day, the supernatant of each sample was acidified with FA and desalted on C18 SepPAC50 columns (WATERS). Peptides were eluted with 0.1% FA in 50% ACN and vacuum dried. Dry peptides were reconstituted in 0.1% FA prior to liquid chromatography-coupled mass spectrometry (LC-MS/MS) analysis.

LC-MS/MS analysis was then performed on an Eclipse mass spectrometer (Thermo Fisher Scientific) coupled on-line to a Dionex Ultimate 3000 RSLCnano system. The liquid chromatography setup consisted of a 75 μm x 2 cm trap column and a 75 μm x 40 cm analytical column, packed in-house with Reprosil Pur ODS-3 1.9 μm particles (Dr. Maisch GmbH). 400 ng peptides were loaded onto the trap column using 0.1% FA in water at a flow rate of 5 μl/min and separated using a 110 min linear gradient from 4% to 32% of solvent B (0.1% (v/v) formic acid, 5% (v/v) DMSO in acetonitrile) at 300 nl/min flow rate. nanoLC solvent A was 0.1% (v/v) formic acid, 5% (v/v) DMSO in HPLC grade water. The Eclipse mass spectrometer was operated in data independent acquisition (DIA) and positive ionization mode. DIA was performed with one full MS event followed by 40 MS/MS windows in one cycle resulting in a cycle time of 3 s. The full MS settings included an automatic gain control (AGC) target value of 100% in the 360–1,300 m/z range with a maximum injection time (max IT) of 50 ms and a resolution of 120,000 at m/z 200. 40 variable DIA precursor windows ranged from 368 m/z (lower boundary of 1st window) to 1179 m/z (upper boundary of 40th window). Precursor ions were fragmented by higher energy collision induced dissociation (HCD) and a normalized collision energy of 30%. MS/MS spectra were acquired with an AGC target value of 1000% for the precursor window with a max IT of 54 ms and a resolution of 30,000 at m/z 200.

### LC-MS/MS data analysis

DIA-NN version 1.8.1 was used to generate an in-silico predicted spectral library composed of the human proteome (UniprotKB reference proteome, UP000005640, download 01/2021) and common contaminants (MaxQuant contaminants.fasta) with trypsin as digestion enzyme and one missed cleavage specified. Subsequently the acquired raw files were processed in library-free mode using DIA-NN default settings and the match between runs function enabled. The iq R script was used to calculate MaxLFQ protein intensity values. If not stated otherwise, all protein abundance values refer to log2 transformed MaxLFQ protein intensity values. Sample intensities were median centered and Principle component Analysis (PCA) was performed on the 5739 protein IDs with quantitative values in all samples.

### Statistical analysis

GraphPad Prism 8.0 software (GraphPad Software, San Diego, CA, USA) was used for statistical analysis.

## Results

### GPX4 inhibition can trigger ferroptotic and apoptotic cell death

**Fig. 1:**
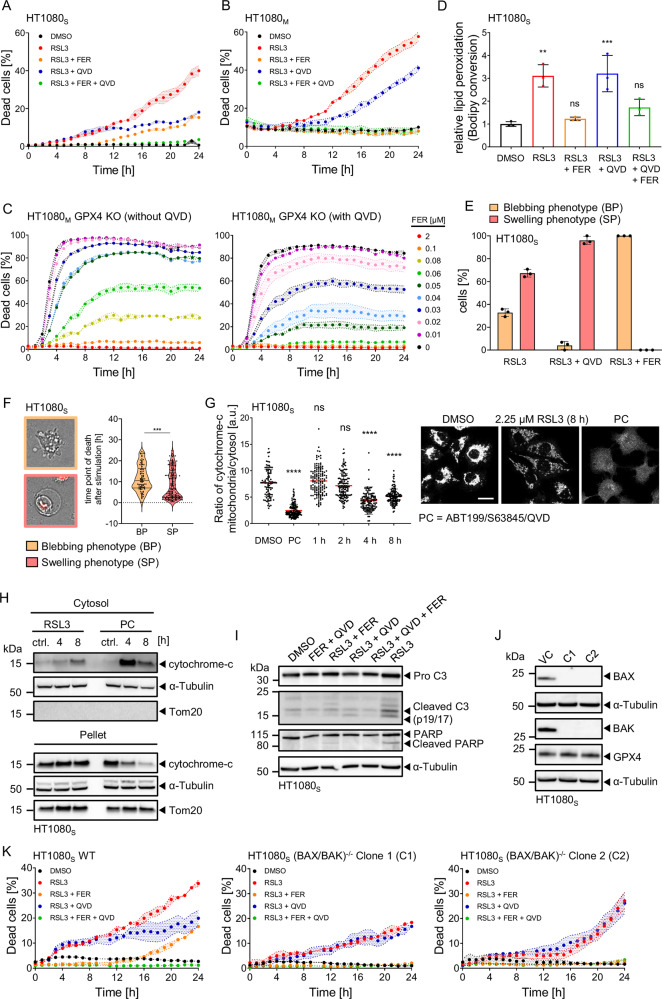
GPX4 inhibition can trigger ferroptotic and apoptotic cell death. **A**, **B** Quantification of cell death, calculated as percentage of PI-positive cells. Cells were stimulated with 4.5 µM RSL3 (HT1080_S_), 300 nM RSL3 (HT1080_S_), 50 µM QVD and 2 µM ferrostatin-1 (FER). Data are the mean ± range of technical duplicates (**A**) or mean ± SEM of technical triplicates (**B**). Panels show one representative examples of at least three independent experiments. **C** Cell death was determined for cells cultured in presence or absence of 50 µM QVD and decreasing amounts of FER. Data are means ± SEM of technical triplicates of one representative example of three independent experiments. **D** Cells were stimulated with 4.5 µM RSL3 for 24 h and analysed for lipid peroxidation (C11 BODIPY 581/591 conversion) via flow cytometry. Data show mean values ± SD of three independent experiments. Asterisks indicate statistical significance compared to the control group (*p ≤ 0.05, **p ≤ 0.01; One-way Anova with Tukey’s post hoc test). **E** Cells were stimulated with 2.25 µM RSL3 and monitored at 10 min intervals via life-cell-imaging. Data are the mean ± SD of three independent experiments. **F** Data show median and quartiles of cells pooled from three independent experiments. Asterisks indicate statistical significance (***p ≤ 0.001; Mann-Whitney test). **G** Subcellular analysis of cytochrome-c redistribution. Cells were stimulated with 2.25 µM RSL3 for the indicated time points or with 10 µM ABT-199 + 10 µM S63845 + 50 µM QVD for 4 h as apoptotic positive control (PC). Fixed cells were immunostained for cytochrome-c and segmented based on MitoTracker signals. Scale bar in representative images, 20 µm. Data show means and scatter from one out of three representative experiments, with about 100 cells per condition. Asterisks indicate statistical significance compared to the control group (****p ≤ 0.0001; ns = not significant, One-way Anova with Tukey’s post hoc test). **H** Cells were stimulated with 4.5 µM RSL3 or 10 µM ABT-199 + 10 µM S63845 + 50 µM QVD (Positive ctrl. = PC) and fractioned into cytoplasm and pellet containing the mitochondria. One representative of three independent experiments is shown. **I** Immunoblotting of caspase-3 (C3) and PARP cleavage in HT1080_S_ cells upon RSL3 (4.5 µM) treatment for 16 h, one representative of three independent experiments is shown. **J** Immunoblots of BAX, BAK and GPX4 expression in vector control cells (VC) and HT1080_S_ (Bax/Bak)^−/−^ clones. **K** Quantification of cell death, calculated as percentage of PI-positive cells. Cells were stimulated with 2.25 µM RSL3, 50 µM QVD and 2 µM FER. Data show the mean ± range of technical duplicates of one representative example of four independent repeats.

Ferroptosis and apoptosis are widely considered to be independent processes resulting in lytic or non-lytic cell death, respectively. Interestingly, we observed in two strains of HT1080 cells (Supplemental Fig. 1A,B), the most widely used human cellular model system for ferroptosis studies, that GPX4 inhibition by RSL3 resulted in cell death that could be prevented partially by pan-caspase inhibitors Q-VD-OPh or z-VAD-FMK (Fig. 1A, B, Supplemental Fig. 1C-E). Suppression of cell death upon caspase inhibition was observed also when reducing RSL3 concentrations (Supplemental Fig. 1F,G), or when inducing cell death by erastin, as an inhibitor of the cystine-glutamate antiporter system x_c_^−^ (Supplemental Fig. 1H-K**)**. Q-VD-OPh also prevented cell death induced by submaximal RSL3 concentrations in HT29 colorectal cancer cells and Pfa1 mouse embryonic fibroblasts (Supplemental Fig. 2A, B). Adding lipid ROS scavenger ferrostatin-1 fully prevented cell death, at least within the first 12 hours of treatment (Fig. 1A, B, Supplemental Fig. 1C-G), indicating that oxidative lipid damage appears to be the primary damage and prerequisite for a fraction of cells to develop a dependence on caspase activity to execute cell death. Data from surviving cells supported this further, since cells treated with RSL3 or erastin proliferated faster upon co-treatment with ferrostatin-1 when compared to Q-VD-OPh co-treatments (Supplemental Fig. 2C–F). Still, caspase inhibition appears to clearly support survival and proliferation of GPX4-deficient cells, which rely on the continuous presence of ferrostatin-1, but which required substantially lower ferrostatin-1 concentrations in the presence of Q-VD-OPh (Fig. 1C, Supplemental Fig. 2G). In line with this, cells that were treated with RSL3 and Q-VD-OPh, followed by drug washout, were able to survive and proliferate (Supplemental Fig. 3A–D). Importantly, Q-VD-OPh did not prevent lipid peroxidation upon RSL3 treatment, as indicated by measuring C11 BODIPY conversion in cells as well as STY-BODIPY conversion in a cell free FENIX assay (Fig. 1D, Supplemental Fig. 3E), confirming that the cell-protective effect of caspase inhibition is downstream of lipid peroxidation arising from GPX4 inhibition. Reducing the concentration of Q-VD-OPh showed that the concentration needed to prevent full maturation of caspase-3 and PARP cleavage corresponded to the concentrations required to reduce cell death (Supplemental Fig. 3F, G).

Morphological studies of dying cells demonstrated that both cell swelling, a phenotype expected during the final stages of cells succumbing to ferroptosis, as well as cell shrinkage and membrane blebbing, indicative of apoptosis execution, could be observed in cells dying upon GPX4 inhibition (Supplemental Movie 1). A substantial portion of cells displayed, sometimes transient, membrane blebbing before inflating and taking up propidium iodide as a marker of impaired plasma membrane integrity. A quantification of these phenotypes in dying cells revealed that approximately one third of cells displayed membrane blebbing, transiently or terminally, following RSL3 treatment, and that this phenotype could be suppressed by caspase inhibition (Fig. 1E). Cells with blebbing phenotypes tended to die later than cells that ruptured following cell swelling (Fig. 1F). This indicates that within the intra-population heterogeneity of cell death phenotypes, features of apoptosis execution kinetically manifest somewhat later than cell death that is primarily ferroptotic. Taken together, these data support the notion that apoptosis signalling appears to contribute to cell death upon GPX4 inhibition, preferentially in fractions of cells that tend to die at later times.

Further evidence for engagement of apoptotic signalling upon GPX4 inhibition was obtained from analysing cytochrome-c distributions. RSL3 treatment resulted in cytochrome-c release into the cytosol, as evidenced by subcellular immunofluorescence analysis, yet less prominently than in positive controls of effective mitochondrial outer membrane permeabilisation (Fig. 1G, Supplemental Fig. 4). Corresponding results were obtained by biochemical analysis of cytosolic cell extracts, in which cytochrome-c could be detected following GPX4 or system x_c_^−^ inhibition (Fig. 1H, Supplemental Fig. 5A–D). This finding aligned with the observation that RSL3 treatment induced effector caspase-3 processing and, as proof of effector caspase activity, cleavage of PARP (Fig. 1I). Apoptosis engagement was BAX/BAK dependent, since cells fully resistant to canonical induction of mitochondrial apoptosis (Fig. 1J, Supplemental Fig. 5E) were partially protected from RSL3 and erastin, quantitatively identical to caspase inhibition by Q-VD-OPh co-treatment (Fig. 1K, Supplemental Fig. 5F). In line with this, cytochrome-c was not released in HT1080 (BAX/BAK)^−/−^ cells and neither were procaspase-3 or PARP processed (Supplemental Fig. 6).

Overall, these data therefore suggest that stress and cellular damage arising from GPX4 inhibition can induce cell death that proceeds with ferroptotic but also apoptotic features, wherein the latter requires the engagement of the mitochondrial apoptosis pathway.

### BH3-mimetics can synergistically enhance cell death induced by GPX4 inhibition

Mitochondrial apoptosis is governed by the BCL-2 protein family, whose anti-apoptotic family members can be targeted by highly specific pharmacological inhibitors (BH3-mimetics). BH3-mimetics are not only widely used in fundamental research but also are of substantial clinical interest as novel therapeutic candidates to combat otherwise non-responsive cancers in single or combination treatments [12]. Due to the indications for mitochondrial apoptotic engagement, we hypothesised that BH3-mimetics could promote cell death induced by GPX4 inhibition and possibly shift outcomes between ferroptotic and apoptotic cell death modalities.

**Fig. 2:**
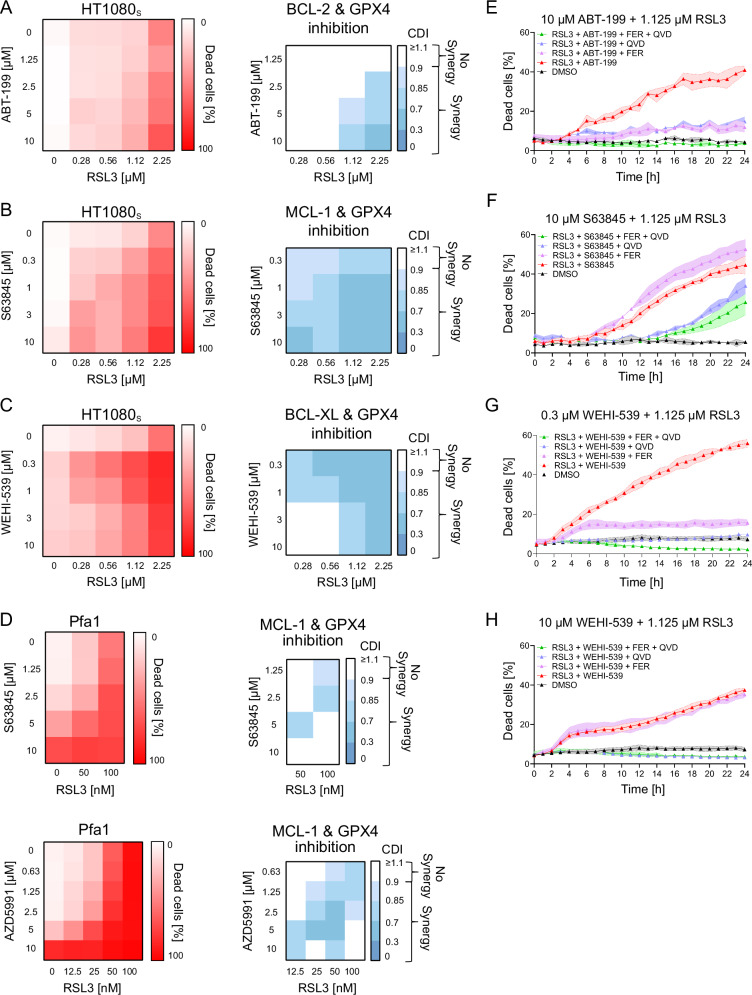
BH3-mimetics sensitise cells to RSL3-induced cell death. **A**–**D** Left heat map: Quantification of cell death, calculated as percentage of PI-positive cells. Cells were stimulated with the indicated concentrations of RSL3, the BCL-2 inhibitor ABT-199, the MCL-1 inhibitors S63845 or AZD5991, or the BCL-XL inhibitor WEHI-539 for 24 h. Data shown are means of two or three technical replicates. Right heat map: The coefficient of drug interaction (CDI) was calculated as Webb’s fractional product. **E**–**H** Cell death was determined by PI uptake. Cells were treated as indicated, in the presence or absence of ferrostatin-1 (FER, 2 µM) or Q-VD-OPh (QVD, 50 µM). Data are mean ± SD of three technical replicates. Panels show one representative example of at least 2 independent experiments.

We therefore treated HT1080_S_ cells with concentration combinations of RSL3 and the clinically used BCL-2 inhibitor ABT-199, the BCL-XL inhibitor WEHI-539, or the MCL-1 inhibitor S63845. In all cases, combination treatments strongly and synergistically enhanced cell death (Fig. 2A–C). Similar results were obtained for Pfa1 cells treated with RSL3 in combination with MCL-1 inhibitor S63845 or the alternative MCL-1 inhibitor AZD5991 (Fig. 2D). In another ferroptosis-susceptible cell line, U87, AZD5991 in combination with RSL3 did not result in overt synergies at the measurement endpoint, but substantially accelerated cell death execution, likewise highlighting a positive interaction in this dual drug treatment (Supplemental Fig. 7).

We next investigated the influence of BH3-mimetics on the type of cell death induced by RSL3. Synergistic cell death of HT1080_S_ cells exposed to RSL3 and ABT-199 could be partially prevented by both Q-VD-OPh and ferrostatin-1 (Fig. 2A, E), demonstrating both ferroptotic and apoptotic contributions to cell death. In contrast, the synergistic cell death upon combining RSL3 with the MCL-1 inhibitor S63845 predominantly induced cell death that was no longer prevented by ferrostatin-1, but partially by Q-VD-OPh, indicating a shift towards apoptosis as the primary terminal cell fate (Fig. 2F). The synergistic induction of cell death by the combination of RSL3 and WEHI-539 was most pronounced at lower amounts of the BCL-XL inhibitor. At these concentrations, cells were partially protected by ferrostatin-1 but fully rescued by Q-VD-OPh (Fig. 2G). At higher concentrations of WEHI-539, ferrostatin-1 lost its protective function and cell death apparently fully depended on caspase activity (Fig. 2H).

Together these data reveal that the addition of BH3-mimetics can synergistically enhance RSL3-induced cell death and furthermore that this is accompanied by shifting the cell death modality towards apoptosis.

### BH3-mimetics can suppress cell death induced by GPX4 inhibition

**Fig. 3:**
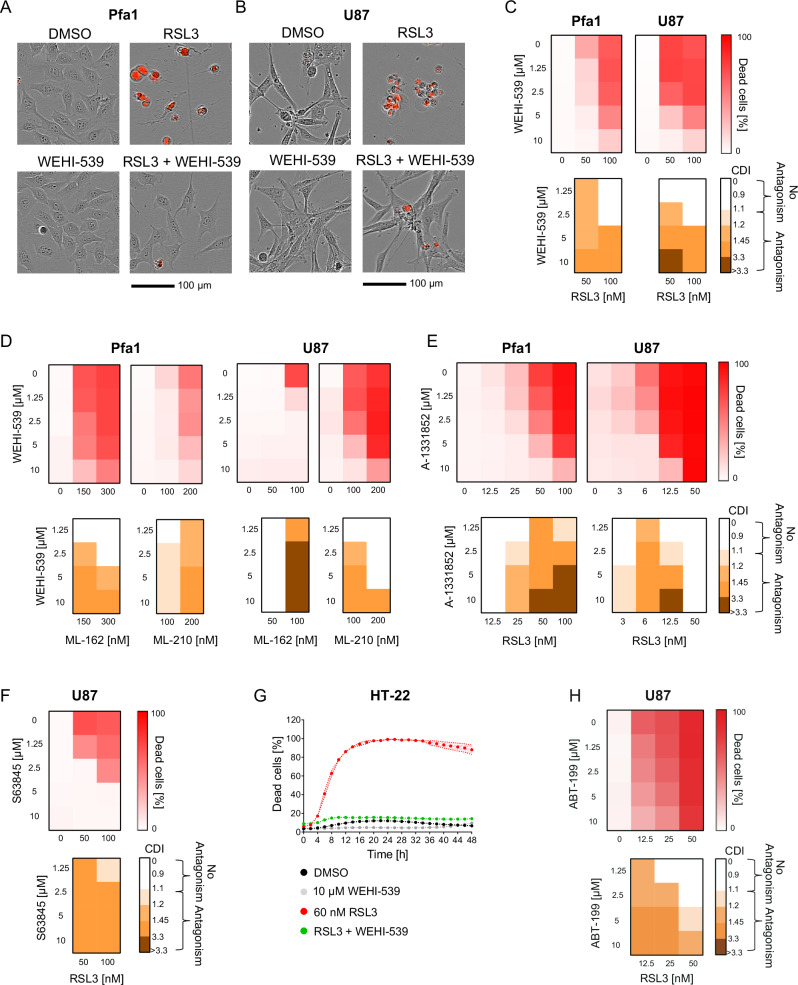
BH3-mimetics can suppress cell death induced by GPX4 inhibition. **A**, **B** Brightfield images with PI fluorescence overlays show cells treated for 24 h with either WEHI-539 (10 µM), RSL3 (100 nM), or both combined. **C**–**F**, **H** Cells were treated for 24 h with the indicated concentrations of GPX4 inhibitors (RSL3, ML-162, ML-210) in combination with BCL-XL inhibitors (WEHI-539, A-1331852), or the MCL-1 inhibitor S63845, or the BCL-2 inhibitor ABT-199. Cell death was determined by PI uptake. Antagonism was determined from the coefficient of drug interaction CDI by the Webb’s fractional product. Data represent the mean of two or three technical replicates, panels show one representative of at least two independent experiments. **G** Quantification of cell death, calculated as percentage of PI-positive cells. Data are the mean ± SEM of technical triplicates. One representative experiment of four independent experiments is shown.

During our analyses on the cell death enhancing effects of BH3-mimetics, we noted that the BCL-XL inhibitor WEHI-539 appeared to reduce peak cell death in HT1080_S_ cells when applied in concentrations of 1 µM and higher (see Fig. 2C). We therefore expanded our studies with this BH3-mimetic to additional cell lines and conditions. To our surprise, WEHI-539 very potently suppressed RSL3-induced death in Pfa1 and U87 cells, as evidenced by reduced PI uptake (Fig. 3A, B) and by reduced LDH release (Supplemental Fig. 8). A screen across a wider range of concentration combinations confirmed WEHI-539 to dose-dependently and potently antagonise RSL3-induced cell death in these cell lines (Fig. 3C). Comparable results were obtained when replacing RSL3 with alternative GPX4 inhibitors, ML-162 and ML-210 (Fig. 3D). A suppression of cell death upon GPX4 inhibition was observed likewise when using A-1331852 as an alternative BCL-XL inhibitor in Pfa1 and U87 cells (Fig. 3E) and when combining the MCL-1 inhibitor S63845 with RSL3 in U87 cells (Fig. 3F). WEHI-539 also suppressed cell death in HT-22 cells (Fig. 3G), a hippocampal neuronal cell line frequently used to study ferroptosis susceptibilities [24, 25], as did ABT-199 in U87 cells (Fig. 3H).

These results reveal a striking contrast to the previous conditions, where synergistic cell death induction had been observed for combination treatments with RSL3 and BH3-mimetics, demonstrating that BH3-mimetics can context-dependently suppress cell death in response to GPX4 inhibition.

### Cell death commitment upon GPX4 inhibition and dependencies on WEHI-539 for cell survival

**Fig. 4:**
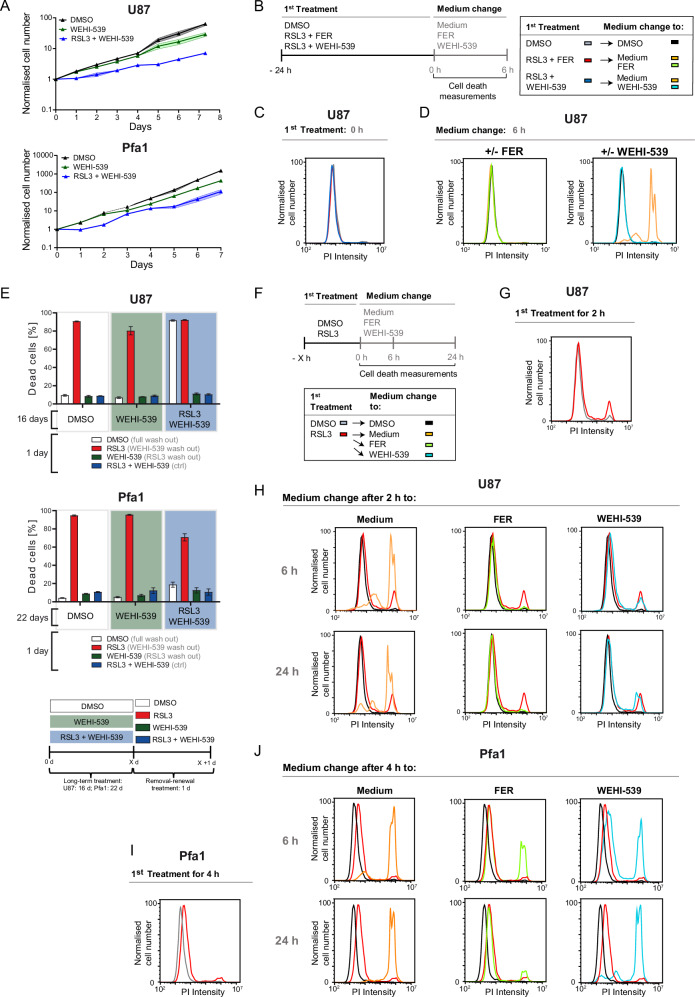
Cell death commitment upon GPX4 inhibition and dependencies on WEHI-539 for cell survival. **A** Cells were treated with WEHI-539 (10 µM) and proliferation was observed for 7 days, both in the presence and absence of RSL3 (100 nM). Supernatants were changed every second day. Data are mean ± SD of technical triplicates from one representative of 2 independent experiments. **B**–**D** The scheme (**B**) illustrates the 24 h treatment schedule with RSL3 (100 nM) and WEHI-539 (10 µM) or ferrostatin-1 (FER, 2 µM), followed by PBS washing. Cell death was measured by PI uptake via flow cytometry after 6 h. Data are from one representative of three independent experiments. **E** Cells were subjected to prolonged treatment with RSL3 (100 nM) and WEHI-539 (10 µM) for 16 days (U87 cells) or 22 days (Pfa1 cells), then washed with PBS and re-exposed as indicated. Cell death was determined by PI uptake. Data shown are mean ± SD of technical triplicates from one representative of two (Pfa1) or three (U87) independent experiments. **F**–**J** Scheme (**F**) displays the treatment schedule. Cells received a 1st treatment with DMSO or RSL3 (100 nM) for 2 h (**G**) or 4 h (**I**). Cells were then washed and exposed to either FER or WEHI-539 (**H**, **J**). Cell death was measured by PI uptake after the first treatment as well as 6 h and 24 h after the medium change. One representative of two independent experiments is shown.

Based on WEHI-539 as a representative BH3-mimetic, we next further studied the potency of ferroptosis antagonism upon GPX4 inhibition as well as effects on cell death susceptibility upon long-term exposure. Both Pfa1 and U87 cells regained proliferative capacity in continuous presence of RSL3/WEHI-539 after approximately 24 h of treatment, with proliferation rates similar to those of controls (Fig. 4A). WEHI-539 therefore not only suppresses cell death but also permits cells to overcome or adapt to RSL3-induced stress, so that proliferation resumes. To understand if differences exist between cell protection by WEHI-539 and ferrostatin-1 in combination treatments with RSL3, drug-containing medium was aspirated and replaced with fresh growth medium after 24 h of incubation (Fig. 4B). U87 control cells resisted 24 h RSL3 treatment in presence of either ferrostatin-1 or WEHI-539 during the combination treatment (Fig. 4C), corresponding to the screening analyses in Fig. 3. However, U87 cells died upon removal of WEHI-539 but not upon removal of ferrostatin-1 from the medium when RSL3 was washed out (Fig. 4D). In Pfa1 cells, only a comparably small fraction of cells died upon washout, yet also here the removal of WEHI-539 was more likely to result in cell death than removing ferrostatin-1 (Supplemental Fig. 9A, C). To also clarify if cells can adapt to RSL3-induced stress in presence of WEHI-539 in the long term, we continuously treated U87 and Pfa1 cells for 2-3 weeks with either WEHI-539 or RSL3/WEHI-539, followed by full drug washout or selective WEHI-539 or RSL3 washout. Both cell lines retained RSL3 responsiveness regardless of whether they were cultured in WEHI-539- or RSL3/WEHI-539-containing medium, yet RSL3 responsiveness was diminished in Pfa1 cells upon full RSL3/WEHI-539 drug washout, as only a fraction of cells committed cell death in this setting (Fig. 4E). Overall, these results indicate that cells grown in medium containing RSL3/WEHI-539 for prolonged periods develop a dependency on the continued presence of WEHI-539 extending to times after which RSL3 has already been removed.

To understand whether a “point of no return” in cell death commitment upon RSL3 exposure can be identified, we next treated U87 cells with RSL3, followed by time-shifted addition of ferrostatin-1 or WEHI-539 (Fig. 4F). A pre-treatment of 2 h was sufficient for the first U87 cells to die from exposure to RSL3 (Fig. 4G). Replacing the supernatant with regular growth medium failed to rescue the majority of cells from dying, whereas both ferrostatin-1 and WEHI-539 prevented cell death execution for at least 24 h of follow up observation (Fig. 4H). In Pfa1 cells, cell death upon addition of ferrostatin-1 or WEHI-539 after a 4 h RSL3 pre-treatment was effectively suppressed by ferrostatin-1. Compared to the control, also WEHI-539 protected Pfa1 cells, yet less efficiently than ferrostatin-1 (Fig. 4I, J). Reducing the pre-treatment duration to 2 h in Pfa1 cells substantially enhanced the capability of WEHI-539 to avert cell death (Supplemental Fig. 10A, B). These data thus show that the BH3-mimetic WEHI-539 can exert its protective effect downstream of the initial GPX4 inhibition and that a point of no return has not yet been (entirely) passed 2-4 h later, since WEHI-539 as well as ferrostatin-1 can still rescue these cells.

### BH3 mimetics exhibit off-target activities that can suppress ferroptotic cell death

**Fig. 5:**
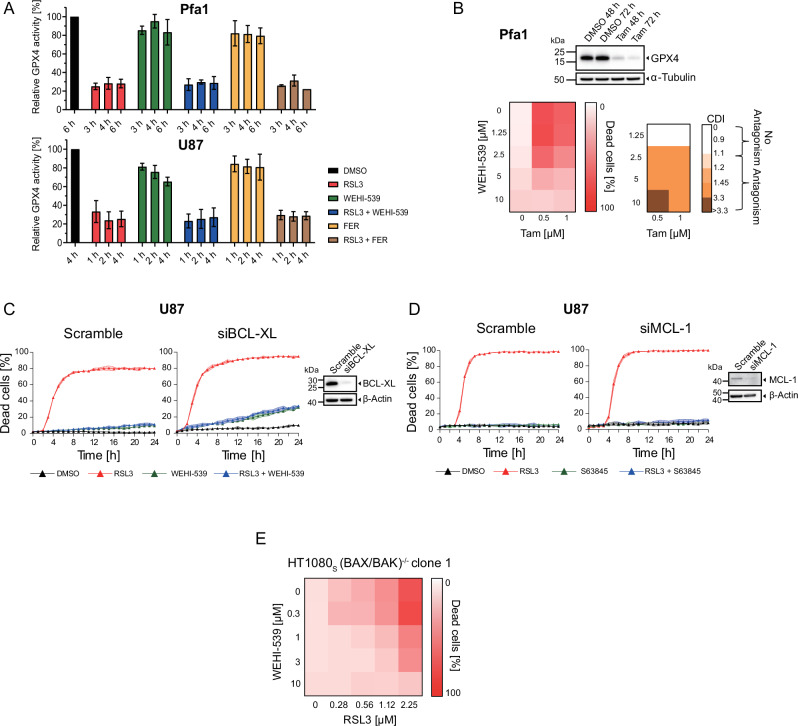
BH3 mimetics exhibit off-target activities that can suppress ferroptotic cell death. **A** Measurement of GPX4 activity. Cells were treated with 10 µM WEHI-539, 2 µM ferrostatin-1 (FER) and 100 nM RSL3 as indicated. Data show mean ± range of two technical replicates from one of two independent experiments. **B** Pfa1 cells with inducible GPX4 knock out were co-treated with WEHI-539 and tamoxifen (Tam) for 72 h. Cell death was determined by PI uptake. Antagonism was calculated by the Webb´s fractional product. GPX4 loss was confirmed by immunoblotting. Data show means from one representative out of three independent experiments. **C** U87 cells were treated with RSL3 (100 nM), WEHI-539 (10 μM), following depletion of BCL-XL expression. Cell death was determined by PI uptake performing live cell imaging. Data shown are means ± SD of three technical replicates from one out of two independent experiments. **D** U87 cells were treated with RSL3 (100 nM), S63845 (10 μM), following depletion of MCL-1 expression. Cell death was determined by PI uptake performing live cell imaging. Data shown are means ± SD of three technical replicates from one out of two independent experiments. **E** Quantification of cell death, calculated as percentage of PI-positive cells. Cells were stimulated with the indicated concentrations of RSL3 and WEHI-539 for 24 h. Data shown are means of two technical replicates. One representative example of three independent experiments is presented.

Next, we evaluated if off-target effects could be responsible for the anti-ferroptotic activity of BH3-mimetics. First, we studied if direct neutralisation of RSL3 by WEHI-539 can be ruled out. In both U87 and Pfa-1 cells, WEHI-539 failed to prevent RSL3 from inhibiting GPX4 activity, as did ferrostatin-1 (Fig. 5A). Instead of pharmacologically inhibiting GPX4, we also assessed if cells depleted of GPX4 by conditional tamoxifen-induced *Gpx4* targeting [14] could be rescued by WEHI-539. Indeed, WEHI-539 effectively prevented cell death upon GPX4 loss (Fig. 5B). Strikingly, depleting cells of BCL-XL expression did not prevent WEHI-539 from suppressing RSL3-induced cell death (Fig. 5C). Similarly, the MCL-1 inhibitor S63845 could still prevent RSL3-induced cell death when the expression of its primary pharmacological target was suppressed (Fig. 5D). Furthermore, in contrast to promoting cell death in wild type HT1080 cells (Fig. 2C, G), WEHI-539 suppressed cell death in BAX/BAK-deficient HT1080 cells (Fig. 5E). Overall, this indicates that BH3-mimetics prevent ferroptotic cell death through off-target activities independent of apoptosis signalling and downstream of GPX4 inhibition.

### BH3 mimetics suppress oxidative damage by intrinsic antioxidant activities

**Fig. 6:**
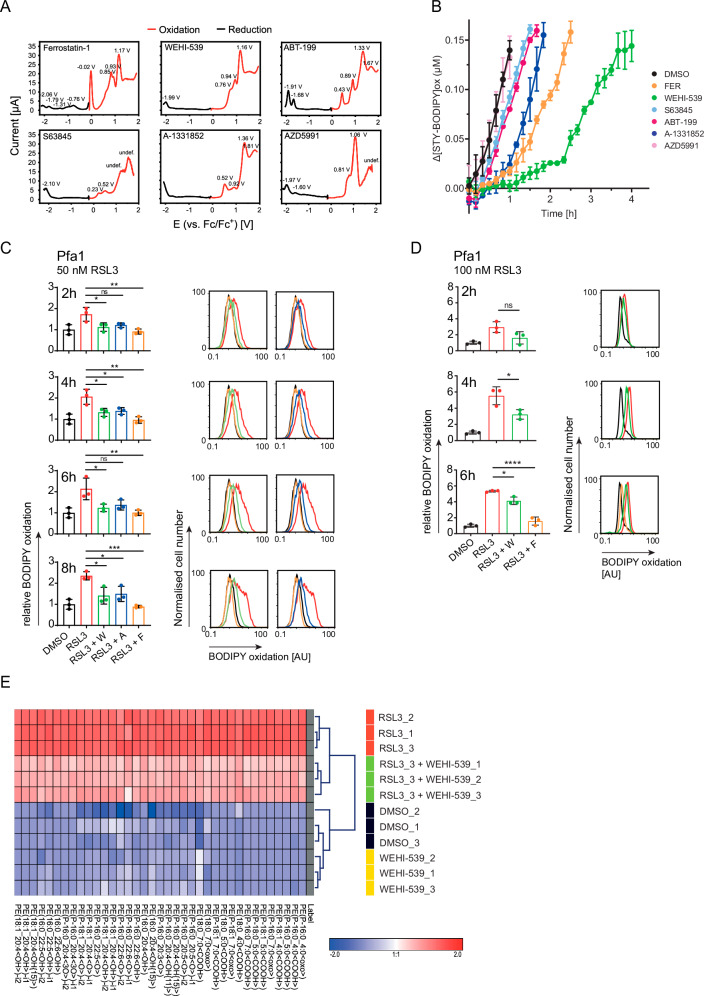
BH3 mimetics suppress oxidative damage by intrinsic antioxidant activities. **A** Voltammetry measurements for ferrostatin-1 (FER) and BH3-mimetics. Red and black curves correspond to the first oxidation or reduction measurement, respectively for each substrate. Peak undefined due to substrate deterioration (undef.). **B** FENIX assay to assess antioxidant activities of FER and BH3 mimetics by measuring STY-BODIPY (1 mM) co-autoxidation. All compounds were tested at 2 μM. Data were normalised to the DMSO control that lacked STY-BODIPY and are presented as the mean ± SD of three technical replicates from one out of three independent experiments. **C** Pfa1 cells were stimulated with 50 nM RSL3, 10 µM WEHI-539 (W),10 µM A-1331852 (A) and 2 µM ferrostatin-1 (F) for the indicated times before they were analysed for lipid peroxidation (C11 BODIPY 581/591 conversion) via flow cytometry. Data shown are mean ± SD of three independent experiments. One-way ANOVA with Tukey’s multiple comparisons test *p ≤ 0.05, **p ≤ 0.01, ***p ≤ 0.001, ns = not significant. Overlay graphs are from one representative experiment. DMSO control (0 h) is the same in all bar graphs and plots. **D** Pfa1 cells were stimulated with 100 nM RSL3, 10 μM WEHI-539 (W) and 2 μM FER (F) for the indicated times before they were analysed for lipid peroxidation (C11 BODIPY 581/591 conversion) via flow cytometry. Data shown are mean ± SD of three independent experiments. One-way ANOVA with Tukey‘s multiple comparisons test *p ≤ 0.05, ****p ≤ 0.0001, ns not significant. Overlay graphs are from one representative experiment. DMSO control (0h) is the same in all bar graphs and plots. **E** Oxidised lipids species measured by LC-MS/MS. Pfa1 cells were treated with 10 µM WEHI-539 and 100 nM RSL3 alone or in combination for 4 h. The heat map shows relative amounts of significantly altered oxidized PEs (ANOVA, adjusted *p* value [FDR] cutoff: 0.05). The color scheme corresponds to auto-scaled log fold change relative to the mean log value within the samples. PE, phosphatidylethanolamines, i indicates isomeric lipids. Samples were clustered by average linkage weighted pair group method with arithmetic mean (WPGMA) agglomeration rule. Data are from one experiment with three technical replicates.

With the anti-ferroptotic activity of BH3-mimetics being off-target activities, it can be speculated that these compounds might have antioxidant activities similar to ferrostatin-1 and other radical trapping agents. To study the electrochemical properties of BH3-mimetics in comparison to ferrostatin-1, we employed differential pulse voltammetry measurements (Fig. 6A). These measure the propensity of substances to accept or donate electrons under externally applied electric potentials. As such, voltammetry can describe the intrinsic redox activity of chemical compounds. Ferrostatin is known to easily donate electrons and prior voltammetry measurements in a methanol environment have been reported before [26]. To compare ferrostatin-1 to BH3-mimetics, we here conducted these measurements in CH_2_Cl_2_ due to the otherwise limited solubility of BH3-mimetics at the concentrations required for comparative measurements. Ferrostatin-1 showed several, mostly overlapping oxidation peaks, with only the first peak being baseline separated. Such a propensity to donate electrons under positive electric potentials was seen for all BH3-mimetics, wherein WEHI-539 showed the closest resemblance to ferrostatin-1. AZD5991 and S63845 appeared unstable, as seen by noisy spectra and undefinable peaks at high positive voltages.

As in vitro readouts for the potency of BH3-mimetics to interfere with self-propagating lipid peroxidation, we compared these to ferrostatin-1 in FENIX assays. Tested at equimolar concentrations, especially the BCL-XL inhibitors WEHI-539 and A-1331852 showed substantial antioxidant activities, whereas AZD5991 was the only BH3-mimetic not separable from the negative control (Fig. 6B). Kinetic analyses *in cellulo* demonstrated that both WEHI-539 and A-1331852 effectively suppressed BODIPY oxidation in Pfa1 cells when treated with 50 nM RSL3 (Fig. 6C, Supplemental Fig. 11A). Similar results were obtained in U87 cells, yet A-1331852 was less effective (Supplemental Fig. 11B, C). At higher concentrations of RSL3 (100 nM), BODIPY oxidation gradually proceeded in the presence of WEHI-539 to become indistinguishable from the positive control after 4–6 h in Pfa1 cells (Fig. 6D). However, despite full BODIPY conversion within this period, which would widely be interpreted as a surrogate indicator for maximal lipid peroxidation, RSL3/WEHI-539 combination treatments clearly did not kill these cells within 24 h of treatment (Fig. 3C). In contrast to the accumulative oxidation of a fixed amount of dye over time, the rate of lipid turnover in living cells might instead permit RSL3/WEHI-539 treated cells to limit the abundance of oxidized lipids to sublethal amounts that do not compromise membrane integrity. We therefore also performed direct lipidomic analyses, which indeed demonstrated that the overall extent of RSL3-induced lipid oxidation was substantially reduced in presence of WEHI-539 (Fig. 6E).

Overall, these data demonstrate that most BH3-mimetics possess intrinsic anti-oxidant activities, with A-1331852 and WEHI-539 showing the strongest effect. Furthermore, these antioxidant activities suffice to reduce lipid oxidation in living cells substantially, affording the survival of sublethally damaged cells.

## Discussion

Here we demonstrate that ferroptosis and apoptosis can manifest as coupled cell death modalities, and furthermore that cell death sensitisation by BH3-mimetics can redirect cell fate outcomes from ferroptosis to apoptosis. Surprisingly, previously undescribed antioxidant properties of BH3-mimetics can counter-intuitively suppress ferroptosis in cells that do not rely on respective BH3-mimetic targets for apoptosis prevention and cell survival under ferroptotic stress.

Hallmarks of apoptosis, such as cytochrome-c release into the cytosol, effector caspase activation and (transient) membrane blebbing, can be observed in cells exposed to ferroptotic stress. This might have remained obscure so far due to prior studies often lacking sufficiently detailed kinetic studies of cell death manifestation under inclusion of appropriate control conditions, or due to applying extreme ferroptotic stress conditions that molecularly and phenotypically left no space for apoptotic hallmarks to manifest prominently. This also applies to the morphological endpoint, since cells that have ruptured and spilled their content obviously can no longer develop an apoptotic phenotype, even if that cell death route had already been initiated in such cells. As we show, apoptosis only partially contributed to overall cell death in HT1080 cells, the most widely used model system to study ferroptosis. The cellular phenotype of apoptotic membrane blebbing [10] often was observed only transiently, before being overridden by cell inflation and membrane rupture. The latter is also observed during apoptosis as a delayed “secondary necrosis” phenotype, especially in absence of the clearing of cellular debris by neighbouring cells or macrophages [27]. However, under ferroptotic stress the features of membrane blebbing and cell lysis kinetically appear tightly coupled and indeed can co-occur in time. Ferroptotic membrane damage therefore likely destabilises membranes to such an extent that apoptotic blebs might lose integrity while cells have already entered the execution phase of apoptosis, thus resulting in rapid cell lysis. Cell lysis is also the endpoint of necroptosis and pyroptosis, two forms of cell death which exhibit strong interplay with apoptosis and during which protein pores permeabilise the plasma membrane [28–31]. Membrane damage by ferroptotic lipid oxidation might promote such lytic cell death modalities in combinatorial stress conditions, as shown for example for gasdermin D-mediated pyroptosis [32], especially if lipid oxidation eases the insertion and interaction of pore forming proteins into and within the plasma membrane. This equally might apply for BAX/BAK-dependent pore formation as a key event during apoptosis signalling at mitochondrial membranes. Unsaturated lipids support BAX and BAK pore formation in membranes [33], and ferroptotic oxidative damage to such lipids might further support the formation of BAX/BAK pores. Indeed, we show here that the presence of BAX/BAK is crucial for apoptosis engagement under ferroptotic oxidative stress. Radical stress is indeed long known to promote apoptosis [34]. A direct mitochondrial link between GPX4 activity and apoptosis resistance under radical stress was also suggested, where GPX4 suppresses the peroxidation of the mitochondrial lipid cardiolipin and prevents the release of cytochrome-c [35], yet these findings have not been validated independently so far.

As shown here, RSL3-induced ferroptotic stress suffices to release submaximal amounts of cytochrome-c from mitochondria into the cytosol, and therefore contrasts with canonical apoptosis induction, where BH3-only proteins induce cytochrome-c release that proceeds in an all-or-none manner and which triggers highly effective effector caspase activation [36, 37]. Limited cytochrome-c release, as seen in our study, in many cases is sufficient to trigger the manifestation of apoptotic hallmarks. This is in contrast to scenarios of residual cytochrome-c release upon low or transient apoptotic stress that nevertheless causes sublethal damage due to effector caspase activation [38–40]. Still, the submaximal cytochrome-c release observed upon RSL3-induced ferroptotic stress likely explains why apoptosis cannot be observed as the primary but nevertheless as a significant contributing cell death modality in our experimental conditions.

To provoke BAX/BAK-dependent permeabilisation of the outer mitochondrial membrane, BH3-mimetics are widely used research tools as well as therapeutically relevant agents [11, 12]. We identified both synergistic interactions with GPX4 inhibition and surprisingly also the opposite, prevention of cell death. It is conceivable that lipid oxidation, which as explained above could enhance apoptosis sensitivity, induces a stronger reliance on cell line-specific subsets of anti-apoptotic BCL-2 family members for survival, and that antagonising these family members results in synergistic rerouting of cell death towards apoptosis. This is in line with a previous study that reported the sensitisation of acute myeloid leukemia cells to ABT-199/venetoxclax upon exposure to RSL3 [41]. Cancer cells escaping treatment can enter a non-genetic state of persistence, and such persister cells tend to develop a dependency on GPX4 activity for survival [42, 43]. Interestingly, this persister state can manifest upon inefficient BH3-mimetic treatments that induce sublethal amounts of MOMP [44]. In this scenario, low amounts of cytosolic cytochrome-c trigger the integrated stress response and an ATF4-dependent metabolic reprogramming that increases cellular sensitivity to GPX4 inhibition. To which extent this might contribute to the synergies we observed in our co-treatment settings, however, remains to be studied. Since we observed residual cytochrome-c release even with RSL3 single-agent treatment, it would likewise be worthwhile to investigate if initially non-lethal GPX4 inhibition could potentially promote metabolic adaptation through submaximal cytochrome-c release that then elevates ferroptosis susceptibility.

Where BH3-mimetics are applied that do not target anti-apoptotic family members required to withstand radical stress, anti-oxidative properties of such BH3-mimetics likely prevail and suppress cell death, as evidenced here by reduced C11 BODIPY and lipid oxidation in cells surviving GPX4 inhibition. Antioxidant properties that reduce cellular ferroptosis susceptibility have been described for various bioactive compounds in a large screening effort in HT1080 cells before [45], yet BH3-mimetics were not studied systematically and across different cell lines. Furthermore, since combination studies with BH3-mimetics and ferroptosis stressors were mostly limited to BCL-2 antagonism [41, 46], and since ABT-199/venetoclax exhibits the least antioxidant activity, the ferroptosis suppressing action of BH3-mimetics has been missed so far. Of note, also other off-target activities of BH3-mimetics have been reported. For example, ABT-199 can trigger metabolic reprogramming of BCL-2-deficient cells and MOMP-incompetent cells through ATF4 as part of the integrated stress response [47]. We therefore cannot formally exclude that additional off-target effects also affect the outcomes of our experiments.

Lipid oxidation upon ferroptotic stress as well as the steady-state lipid oxidation status are not commonly measured directly, and instead most often are determined by the oxidation of C11 BODIPY 581/591 as a surrogate fluorescence marker. While suitable for determining the overall, accumulated lipid oxidation, endpoint BODIPY measurements might not reflect the actual lipid oxidation status in cells surviving ferroptotic stress. As lipid turnover and membrane repair continuously counteract membrane damage [48, 49], high BODIPY signals, especially after prolonged observation times, might overestimate actual lipid oxidation status. Indeed, we observed cells with high endpoint amounts BODIPY oxidation to survive treatment, indicating the requirement for direct lipidomics measurements to reliably assess oxidative lipid damage.

To conclude, likely dependent on the composition of the BCL-2 family interactome and cell line-specific intrinsic apoptotic priming [50], synergistically enhanced cell death or unexpected cell survival in presence of BH3-mimetics and ferroptotic stress will be the predominant outcome. Our work therefore highlights the need to assess case-specifically if combinations of ferroptosis inducers and BH3-mimetics can be suitable combinations to enhance cancer cell killing or if these will rather result in unexpected and unwanted cell survival. This is of particular relevance since we found that cells maintain or regain proliferative capacity in presence of BH3-mimetics with pronounced antioxidant activities, and since µM concentration ranges of BH3-mimetics are achieved as peak serum concentrations in patients [51–54].

## Supplementary information

Supplemental Material - Legends

Supplemental Figure 1

Supplemental Figure 2

Supplemental Figure 3

Supplemental Figure 4

Supplemental Figure 5

Supplemental Figure 6

Supplemental Figure 7

Supplemental Figure 8

Supplemental Figure 9

Supplemental Figure 10

Supplemental Figure 11

Supplemental Figure 12 - uncropped immunoblots

Supplemental Table 1

Supplemental Table 2

Supplemental Movie 1

## Data Availability

Data are available from the authors. Uncropped immunoblots are provided as Supplemental Figure 12.
