# Supplementary material for: Interplay of ferroptotic and apoptotic cell death and its modulation by BH3-mimetics
Source: Cell Death Differ. 2025 Apr 29;32(11):1970–85. doi: 10.1038/s41418-025-01514-7 (PMC12572382; doi:10.1038/s41418-025-01514-7)
Supplement: Supplementary file 1 — Supplemental Material - Legends [file 41418_2025_1514_MOESM1_ESM.docx]

**Supplemental Figure 1**

**(A)** Quantification of cell death, calculated as percentage of PI-positive cells. Data show mean ± range of technical duplicates of one out of two independent experiments.

**(B)** Profiling of HT1080_S_ and HT1080_M_ strains using highly polymorphic short tandem repeat loci (STRs) analysis, indicating 100% authenticity, as well as principal component analysis of 5,739 reliably identifiable protein IDs. Two separate sample digests were performed to generate two technical replicates for each line sample (B1,B2), in-solution digestion (T1) and SP3-digestion (T2). Proteome data show high similarity of HT1080 strains in comparison to two different cell lines.

**(C-K)** Quantification of cell death as in **(A)**. Cells were stimulated with the indicated concentrations of RSL3 and erastin as well as with 50 µM Q-VD-OPh (QVD), 50 µM z-VAD-FMK (zVAD) and 2 µM ferrostatin-1 (FER). Data are means ± range of technical duplicates **(C,E)** and mean ± SEM of technical triplicates (rest) from one out of three independent experiments.

**Supplemental Figure 2**

**(A, B)** Quantification of cell death, calculated as percentage of PI-positive cells. Cells were stimulated with the respective concentrations of RSL3 in the presence or absence of 50 µM Q-VD-OPh (QVD) and/or 2 µM ferrostatin-1 (FER). Data show mean ± range of technical duplicates of one out of two independent experiments.

**(C)** Images were taken at 10x magnification and are from one representative of three independent experiments. Panels show brightfield overlays with PI fluorescence.

**(D-F)** Cell proliferation. Cells were stimulated with the indicated concentrations of RSL3 and erastin as well as with 50 µM QVD and 2 µM FER. Data are means ± SEM of technical triplicates from one out of three independent experiments.

**(G)** Cell proliferation was determined in HT1080_M_ cells lacking GPX4. QVD was used at 50 µM. Data show means ± SEM of technical triplicates from one out of three independent experiments.

**Supplemental Figure 3**

**(A)** Scheme for the long-term survival and proliferation experiment. HT1080 cells were treated with 2.25 µM RSL3 alone or in combination with either 50 µM Q-VD-OPh (QVD) or 2 µM ferrostatin-1 (FER). After 24 h, RSL3 was washed out and the cells were incubated with either DMSO, 50 µM QVD or 2 µM FER. At day 3, cells were washed and grown in media to assess the proliferation capacity of surviving cells.

**(B)** Images were taken at 10x magnification and are from one representative of two independent experiments.

**(C)** Quantification of viable cells, calculated as percentage of PI-negative cells. Data show mean ± SD of technical triplicates of one out of two independent experiments.

**(D)** After drug washout on day 3 (see scheme), cell numbers in each well were normalized to 1, and the relative increase was measured until the end of the experiment on day 6. Data show mean ± SEM of technical triplicates of one out of two independent experiments.

**(E)** FENIX assay to assess antioxidant activities of z-VAD-FMK (zVAD) and QVD by measuring STY-BODIPY (1 mM) co-autoxidation. All compounds were tested at a concentration of 50 µM, with the exception of FER, which was used at 2 µM. Data were normalised to a DMSO control that lacked STY-BODIPY and are presented as the mean ± range of two technical replicates from one out of three independent experiments.

**(F)** Quantification of cell death, calculated as percentage of PI-positive cells. Data show the mean of technical duplicates of one out of three independent experiments.

**(G)** HT1080_S_ cells were stimulated for 8 h with the indicated compounds and full cell extracts were blotted for the specified proteins. QVD-OPH (Q), ferrostatin-1 (FER). One representative of two independent experiments is shown.

**Supplemental Figure 4**

Cells loaded with MitoTracker Red CMXRos were stimulated with the indicated concentrations of RSL3 and with 10 µM ABT-199, 10 µM S63845 and 50 µM QVD-OPH (QVD) for 4 h as an apoptosis positive control. At the respective time points, cells were fixed and immunostained for cytochrome-c. Images are from one representative of three independent experiments.

**Supplemental Figure 5**

**(A-D)** Cells were fractioned into cytoplasm and pellet containing the mitochondria. Fractions were blotted for cytochrome-c. One representative of three (HT1080_S_) or two (HT1080_M_) independent experiments is shown.

**(E)** Quantification of cell death, calculated as percentage of PI-positive cells. Cells were stimulated with 10 µM ABT-199 + 10 µM S63845. Data are means ± range of technical duplicates from one out of three independent experiments.

**(F)** Quantification of cell death, calculated as percentage of PI-positive cells. Cells were stimulated with 4 µM erastin in the presence or absence of 50 µM QVD-OPH (QVD) and/or 2 µM ferrostatin-1 (FER). Data show mean ± range of technical duplicates of one out of three independent experiments.

**Supplemental Figure 6**

**(A-C)** Cells were fractioned into cytoplasm and pellet containing the mitochondria. Fractions were blotted for cytochrome-c. One representative of two (erastin) or three (RSL3) independent experiments is shown.

**(E, F)** Cells were stimulated with the indicated compounds and full cell extracts were blotted for the specified proteins. One representative of two independent experiments is shown.

**Supplemental Figure 7**

**(A)** U87 cells were treated as indicated. Cell death was determined by the uptake of PI at 24 h. Heatmap shows the mean of three independent experiments.

**(B, C)** Cells were treated with RSL3 and AZD5991 (10 µM). Cell death was determined by PI uptake using time-lapse imaging. Data show means ± SD from three independent experiments.

**Supplemental Figure 8**

LDH release was measured following treatments with DMSO, 10 µM WEHI-539, 2 µM ferrostatin-1 (FER), or 100 nM of RSL3 alone or in combination. Data are means ± range from one experiment.

**Supplemental Figure 9**

**(A)** Treatment schedule.

**(B, C)** Pfa1 cells received a 1st treatment with DMSO or a combination treatment of RSL3 (100 nM) with WEHI-539 (10 µM) or RSL3 with ferrostatin-1 (FER, 2 µM) for 24 h. Cells were then washed with PBS and the medium was changed as indicated in (A). Cell death was measured by PI uptake after the first treatment and 6 h after the medium change. One representative experiment of three independent experiments is shown.

**Supplemental Figure 10**

**(A)** Treatment schedule

**(B, C)** Cells received the 1st treatment with DMSO or RSL3 (100 nM) for 2 h. Cells were then washed with PBS and the medium was changed as indicated. Cell death was measured by PI uptake at 2 h (B) as well as 6 h and 24 h after the medium change. One out of two independent experiments is shown.

**Supplemental Figure 11**

**(A)** Cells were stimulated with 50 nM RSL3, 10 µM WEHI-539, 10 µM A-1331852 and 2 µM ferrostatin-1. Shown are the controls, mean ± SD of three independent experiments.

**(B, C)** U87 cells were stimulated with 12.5 nM RSL3, 10 µM WEHI-539 (W), 10 µM A-1331852 (A) and 2 µM ferrostatin-1 (F) for the indicated times. Data shown are mean ± range of two independent experiments. Overlay graphs are from one representative experiment. DMSO control (8h) is the same in all bar graphs and plots.

**Supplemental Movie 1**

HT1080_S_ cells were stimulated with 2.25 µM RSL3 and monitored over a time period of 24 h. Images were taken every 10 min at 20x magnification. Medium contained PI. One representative movie of three independent experiments is shown.
