# Supplementary figures and images for: Interplay of ferroptotic and apoptotic cell death and its modulation by BH3-mimetics
Source: Cell Death Differ. 2025 Apr 29;32(11):1970–85. doi: 10.1038/s41418-025-01514-7 (PMC12572382; doi:10.1038/s41418-025-01514-7)

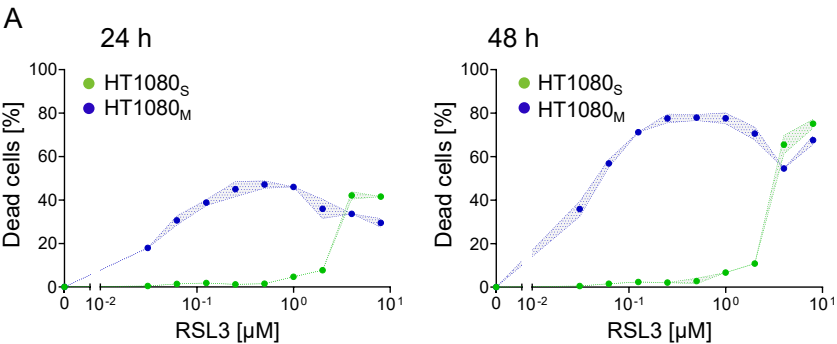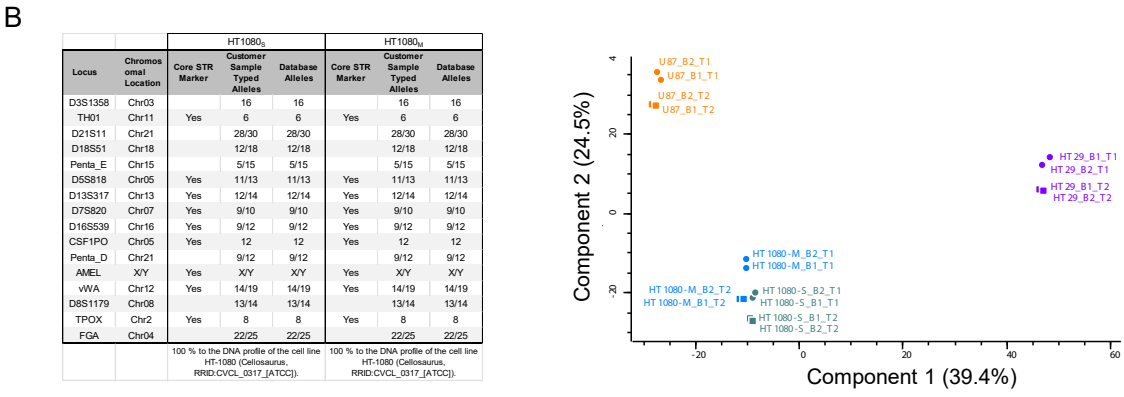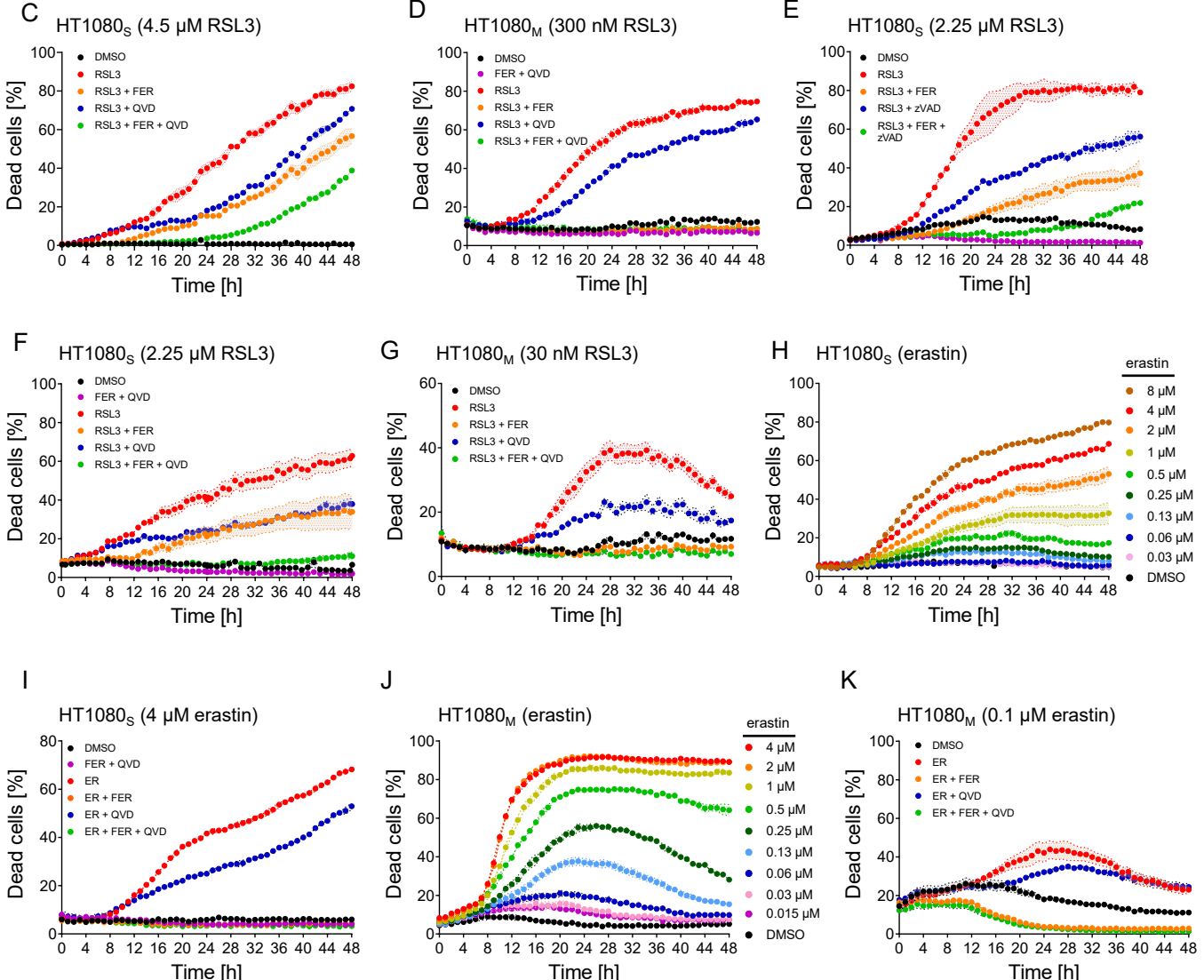

Supplement: Supplementary file 2 — Supplemental Figure 1 [file 41418_2025_1514_MOESM2_ESM.pdf]

A

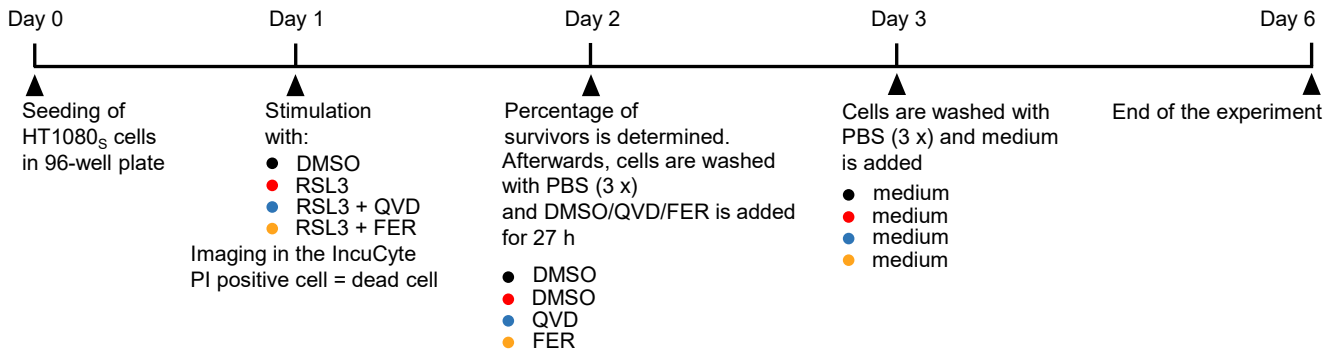

B

Cells after wash out of:

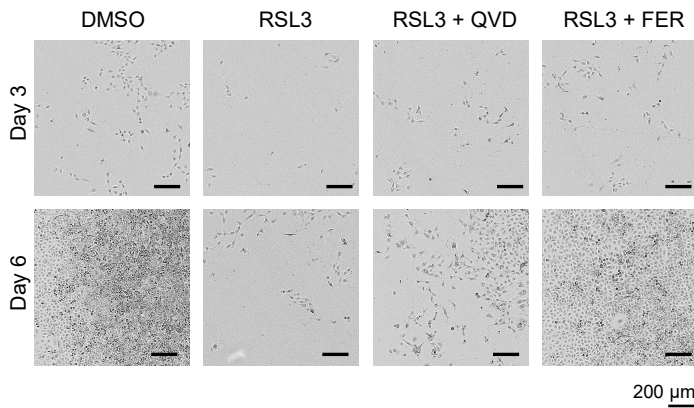

C

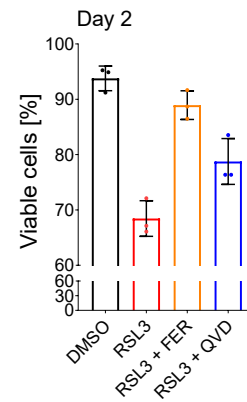

D

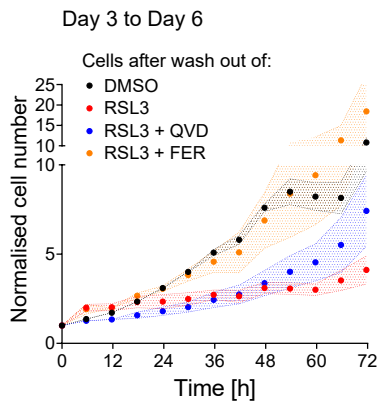

E

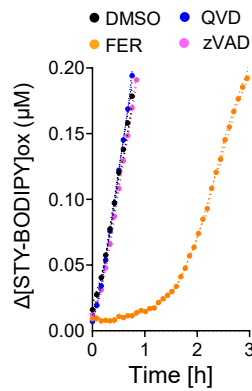

F

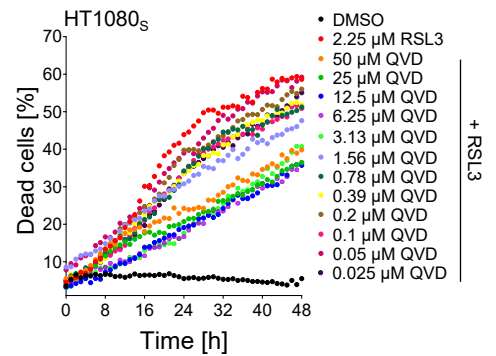

G

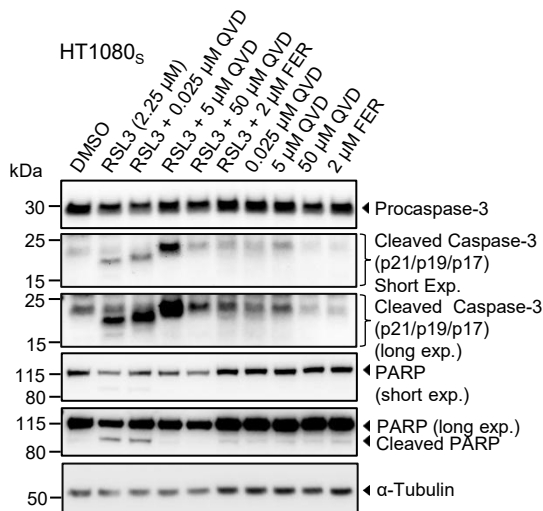

Supplement: Supplementary file 4 — Supplemental Figure 3 [file 41418_2025_1514_MOESM4_ESM.pdf]

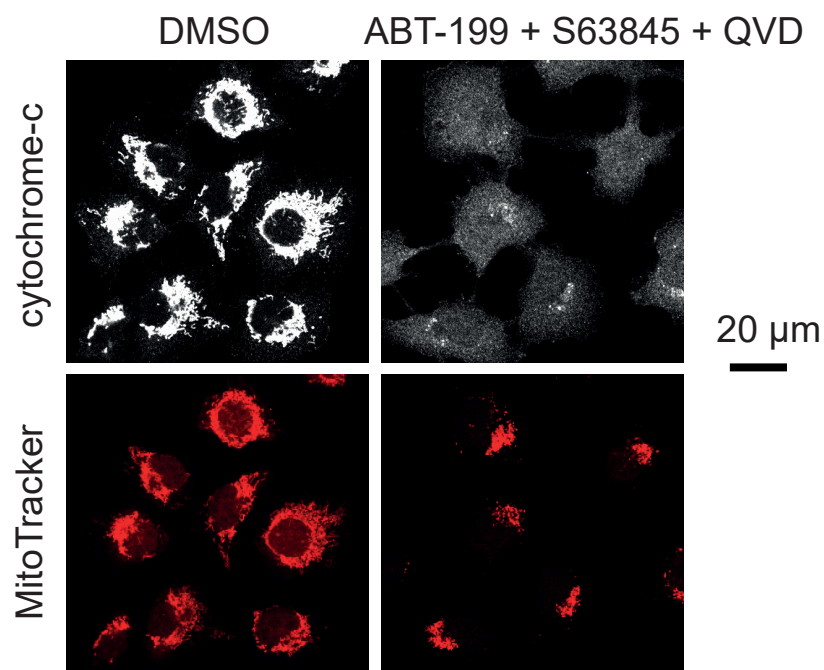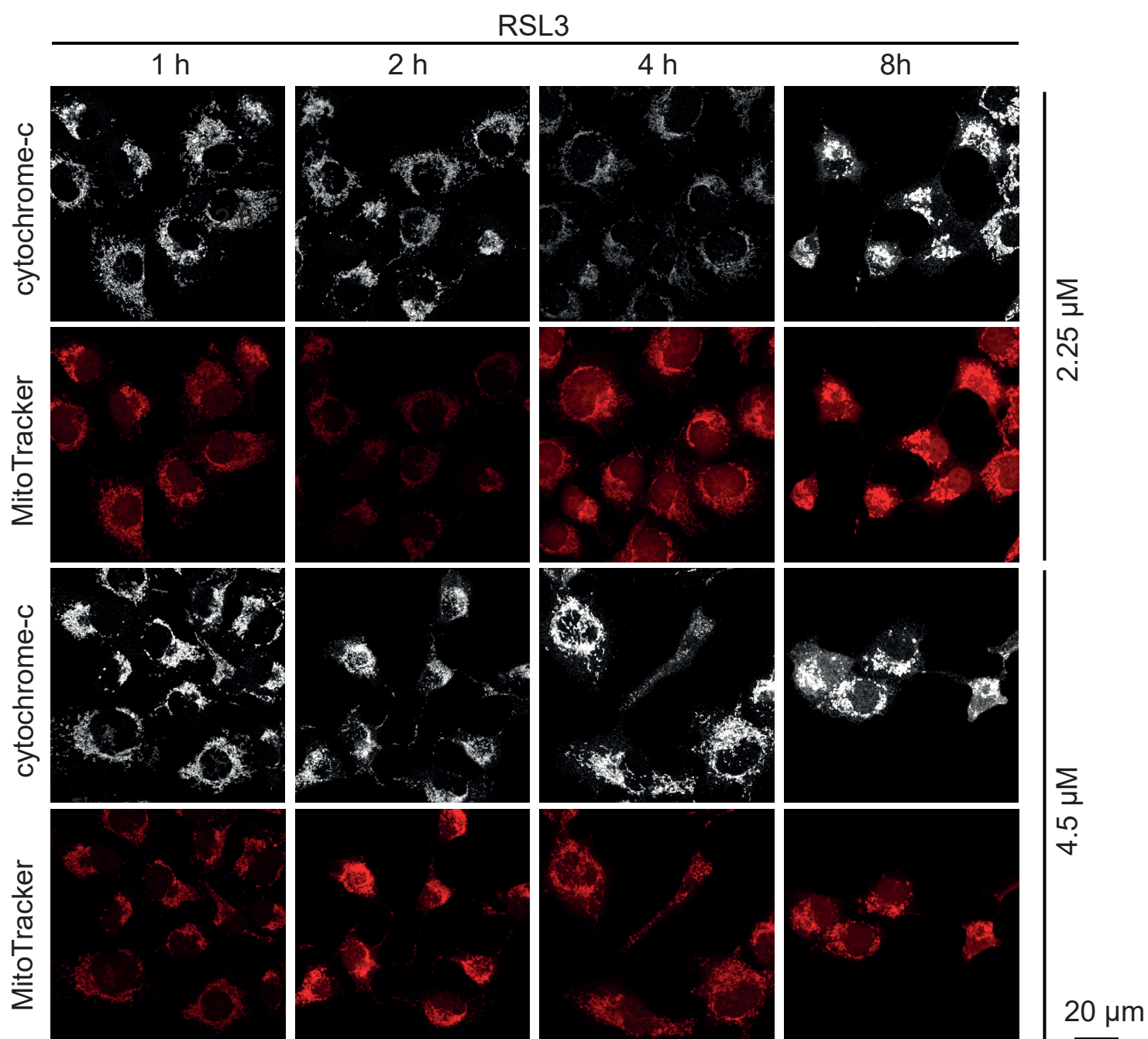

Supplement: Supplementary file 5 — Supplemental Figure 4 [file 41418_2025_1514_MOESM5_ESM.pdf]

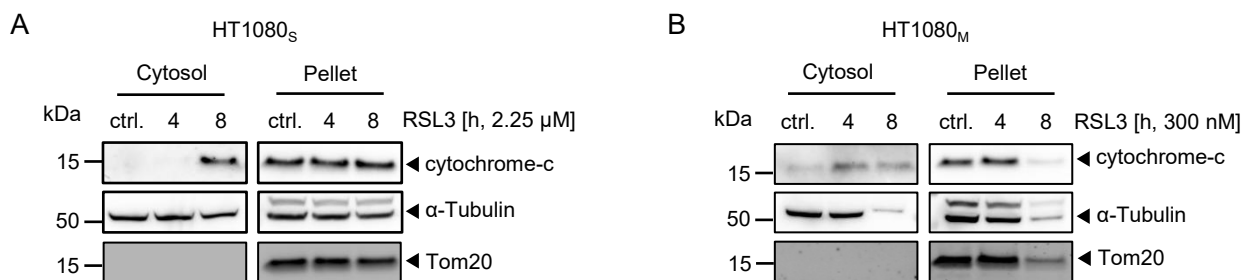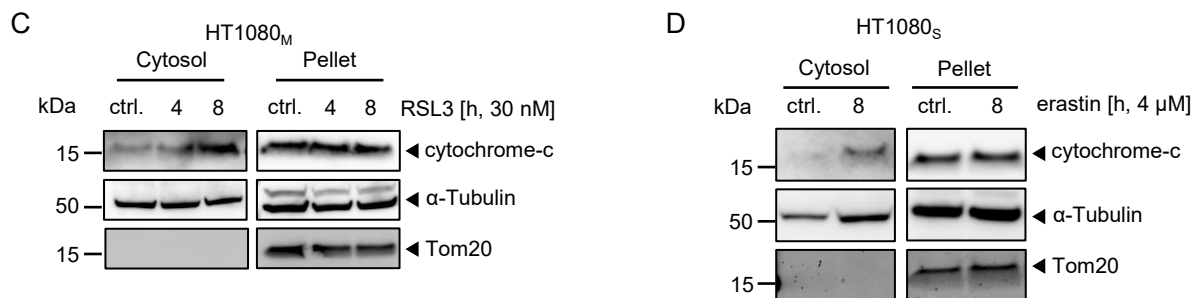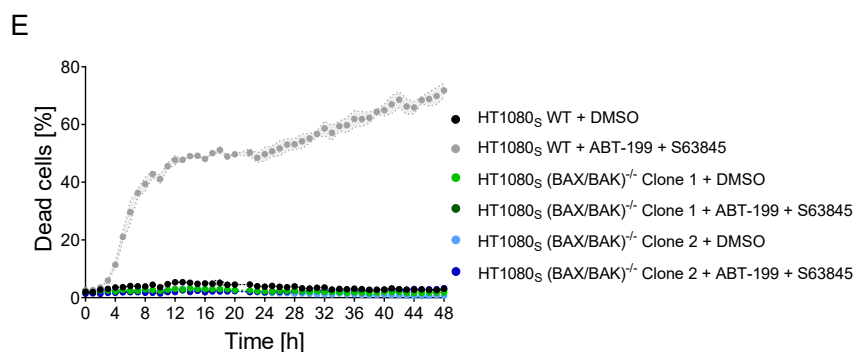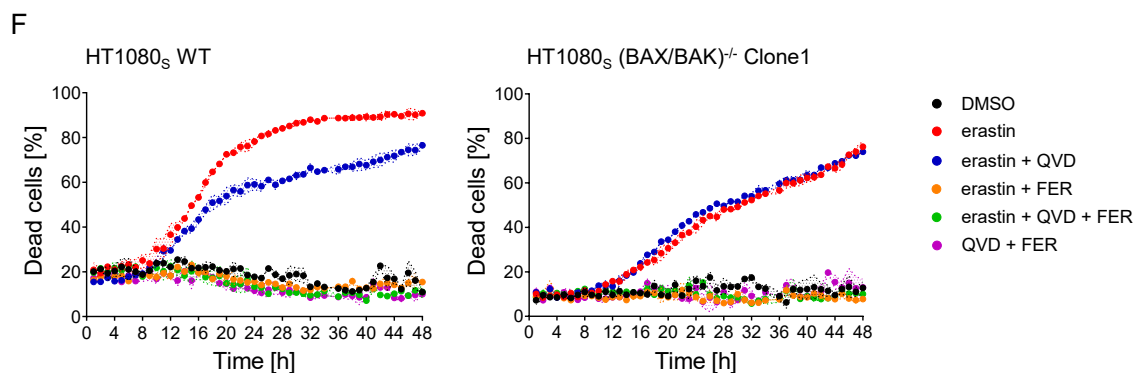

Supplement: Supplementary file 6 — Supplemental Figure 5 [file 41418_2025_1514_MOESM6_ESM.pdf]

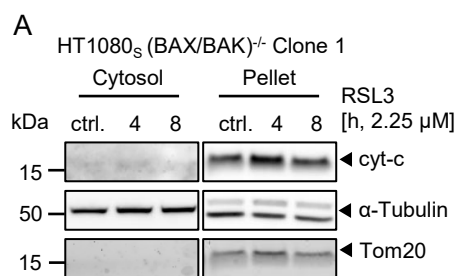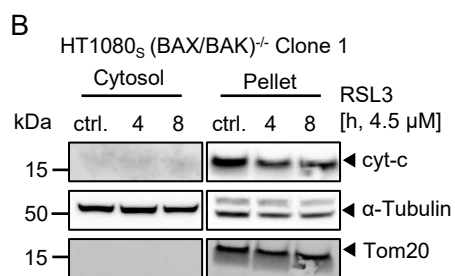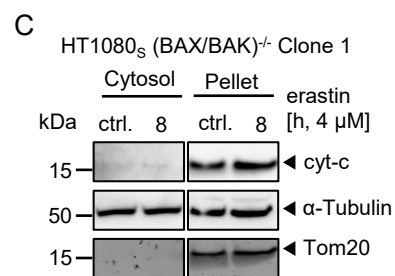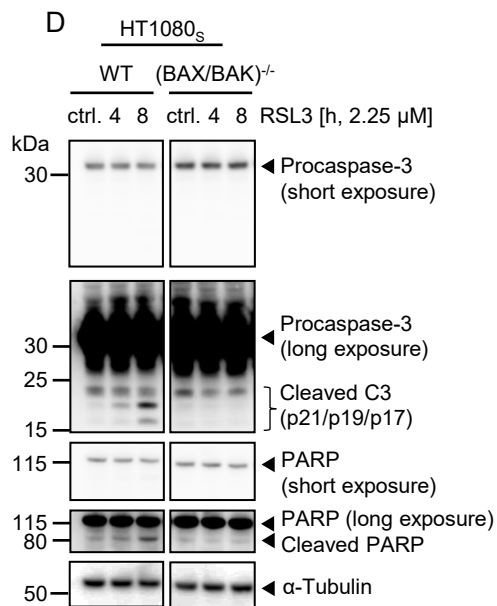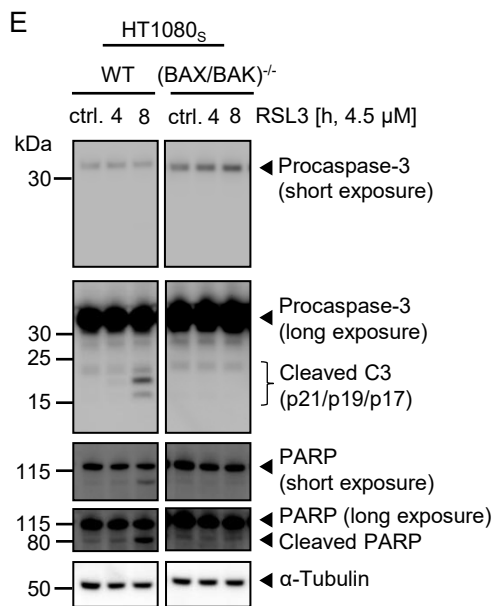

Supplement: Supplementary file 7 — Supplemental Figure 6 [file 41418_2025_1514_MOESM7_ESM.pdf]

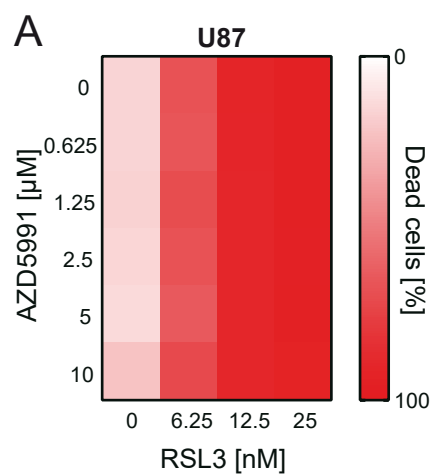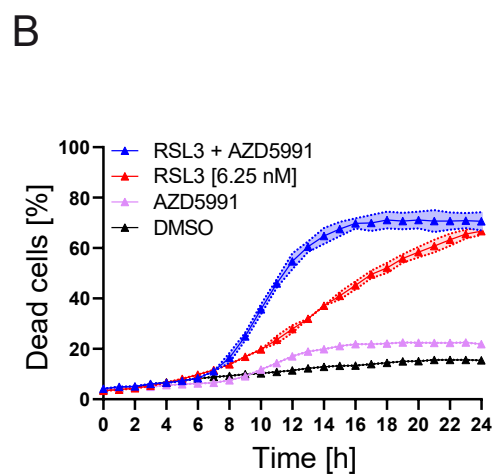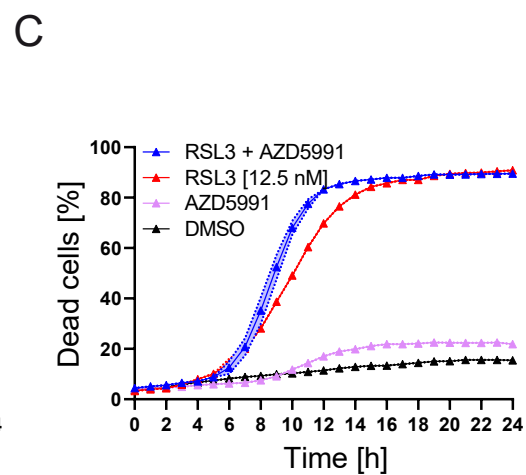

Supplement: Supplementary file 8 — Supplemental Figure 7 [file 41418_2025_1514_MOESM8_ESM.pdf]

**U87**

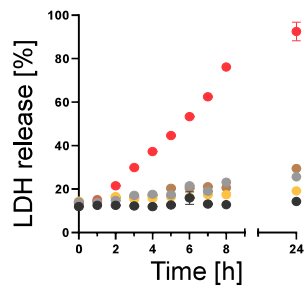

**Pfa1**

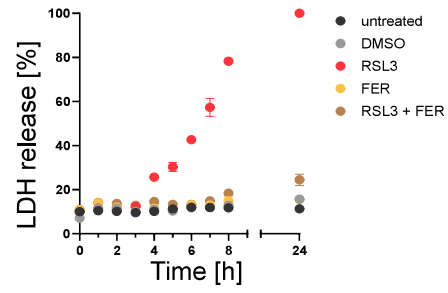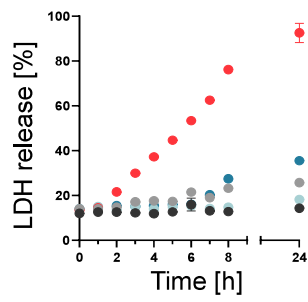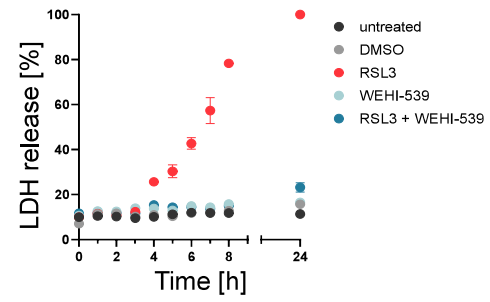

Supplement: Supplementary file 9 — Supplemental Figure 8 [file 41418_2025_1514_MOESM9_ESM.pdf]

**A**

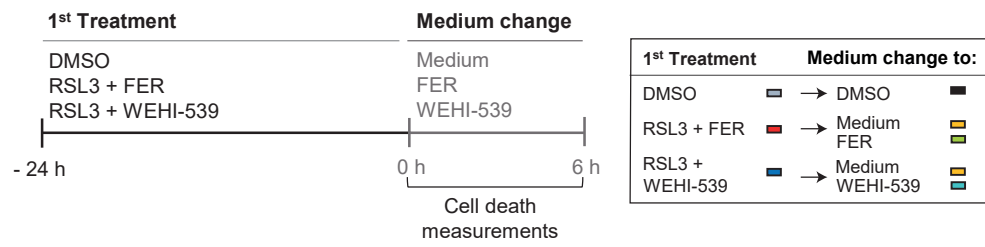

**B**

**Pfa1**

1<sup>st</sup> Treatment for 24 h: 0 h

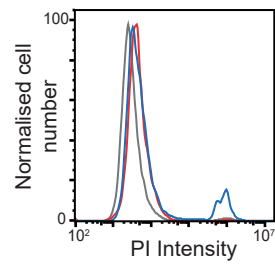

**C**

**Pfa1**

Medium change: 6 h

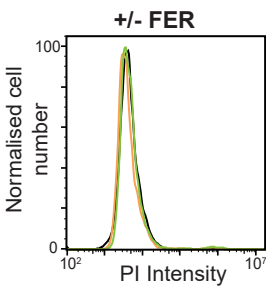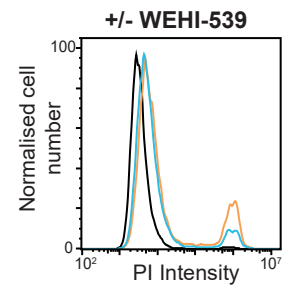

Supplement: Supplementary file 10 — Supplemental Figure 9 [file 41418_2025_1514_MOESM10_ESM.pdf]

A

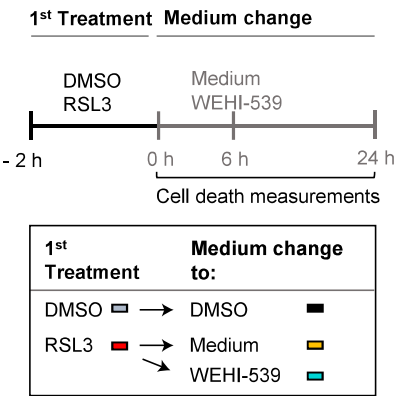

B

Pfa1

1<sup>st</sup> Treatment for 2 h

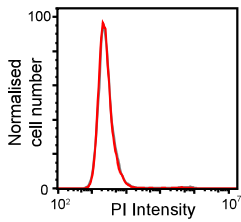

Pfa1

Medium change after 2 h to:

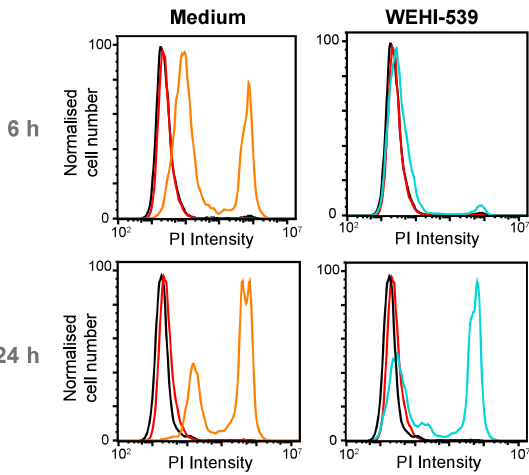

Supplement: Supplementary file 11 — Supplemental Figure 10 [file 41418_2025_1514_MOESM11_ESM.pdf]

A

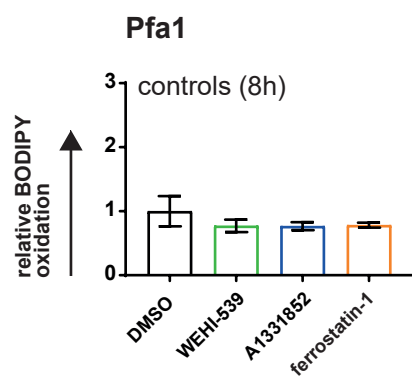

B

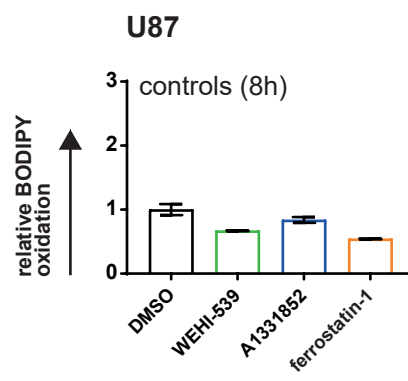

C

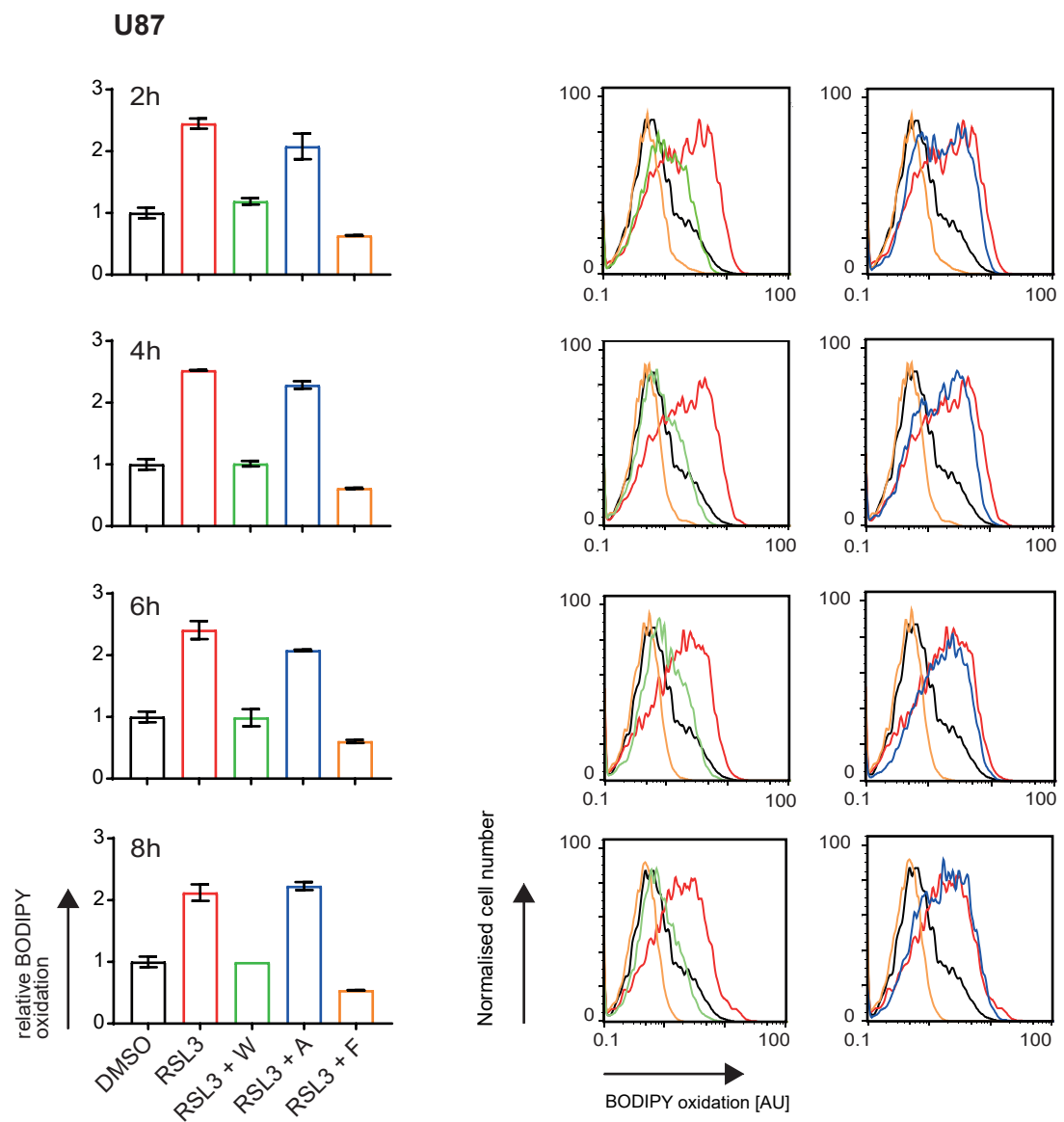

Supplement: Supplementary file 12 — Supplemental Figure 11 [file 41418_2025_1514_MOESM12_ESM.pdf]
