# Supplementary material for: Interplay of ferroptotic and apoptotic cell death and its modulation by BH3-mimetics
Source: Cell Death Differ. 2025 Apr 29;32(11):1970–85. doi: 10.1038/s41418-025-01514-7 (PMC12572382; doi:10.1038/s41418-025-01514-7)
Supplement: Supplementary file 3 — Supplemental Figure 2 [file 41418_2025_1514_MOESM3_ESM.pdf]

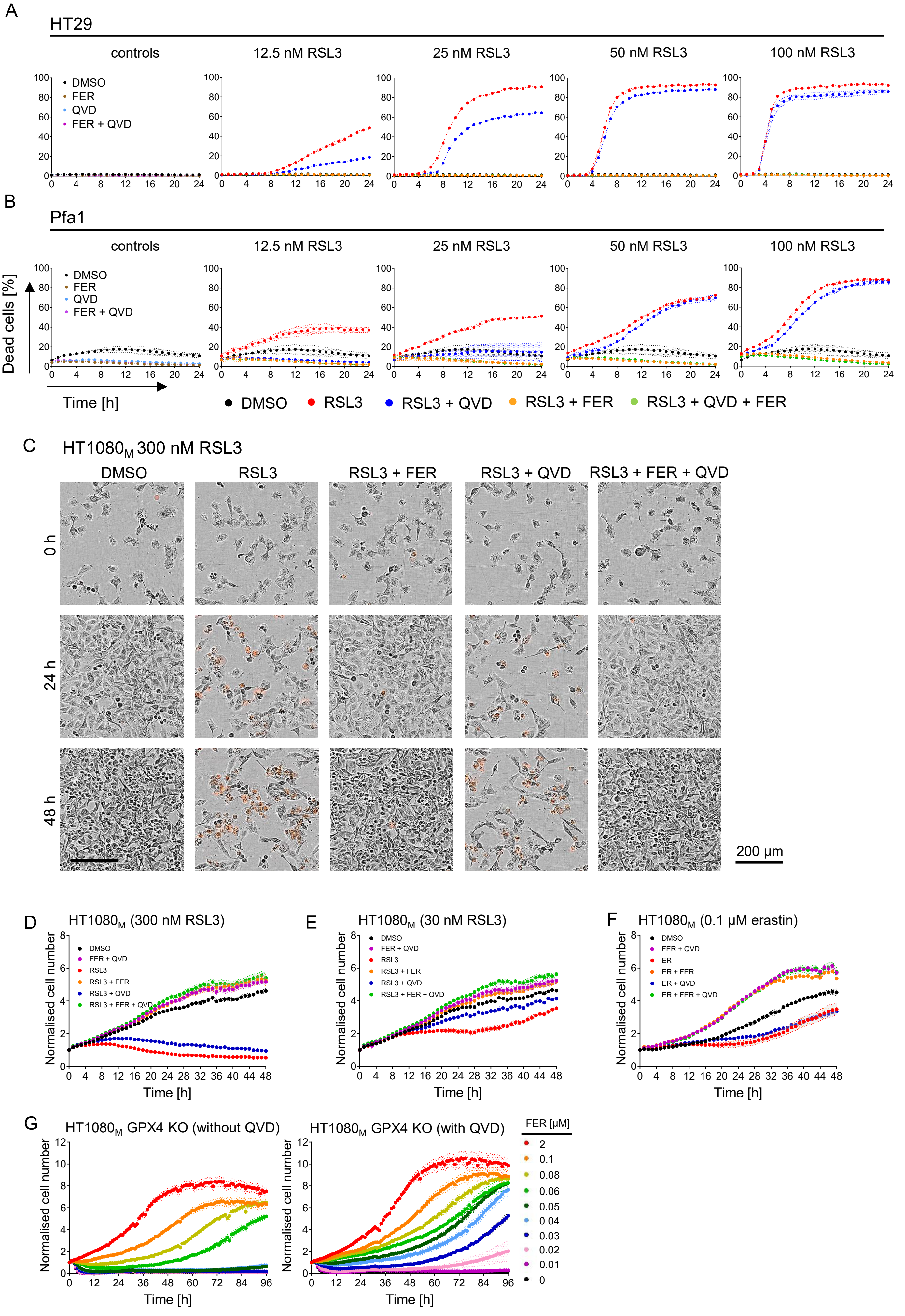

A
HT29
controls
12.5 nM RSL3
25 nM RSL3
50 nM RSL3
100 nM RSL3
DMSO
FER
QVD
FER + QVD
B
Pfa1
controls
12.5 nM RSL3
25 nM RSL3
50 nM RSL3
100 nM RSL3
DMSO
FER
QVD
FER + QVD
Dead cells [%]
Time [h]
DMSO
RSL3
RSL3 + QVD
RSL3 + FER
RSL3 + QVD + FER
C
HT1080M 300 nM RSL3
DMSO
RSL3
RSL3 + FER
RSL3 + QVD
RSL3 + FER + QVD
0 h
24 h
48 h
200 µm
D
HT1080M (300 nM RSL3)
DMSO
FER + QVD
RSL3
RSL3 + FER
RSL3 + QVD
RSL3 + FER + QVD
Normalised cell number
Time [h]
E
HT1080M (30 nM RSL3)
DMSO
FER + QVD
RSL3
RSL3 + FER
RSL3 + QVD
RSL3 + FER + QVD
Normalised cell number
Time [h]
F
HT1080M (0.1 µM erastin)
DMSO
FER + QVD
ER
ER + FER
ER + QVD
ER + FER + QVD
Normalised cell number
Time [h]
G
HT1080M GPX4 KO (without QVD)
HT1080M GPX4 KO (with QVD)
Normalised cell number
Time [h]
FER [µM]
2
0.1
0.08
0.06
0.05
0.04
0.03
0.02
0.01
0
