# Supplementary material for: Interplay of ferroptotic and apoptotic cell death and its modulation by BH3-mimetics
Source: Cell Death Differ. 2025 Apr 29;32(11):1970–85. doi: 10.1038/s41418-025-01514-7 (PMC12572382; doi:10.1038/s41418-025-01514-7)

Figure 1 H

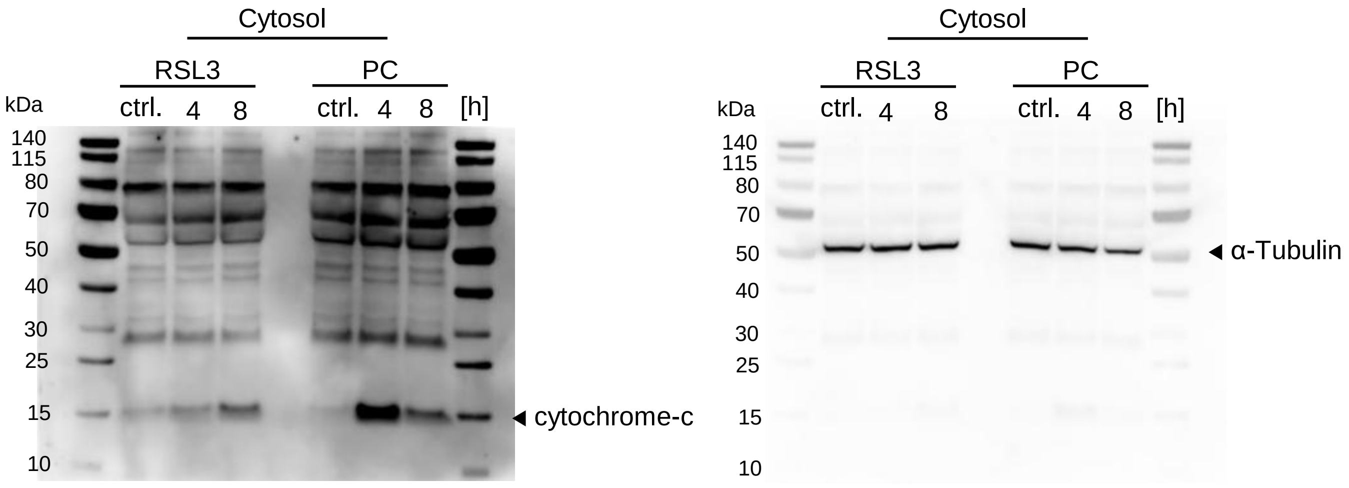

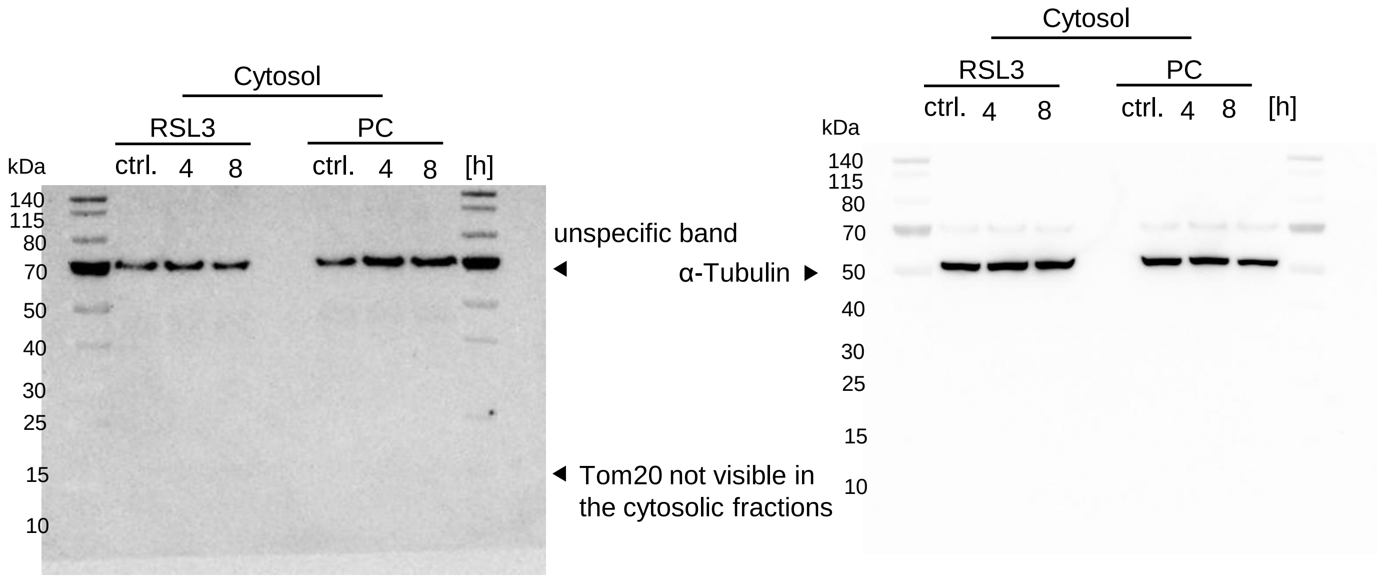

For the detection of Tom20, the same samples as for the detection of cytochrome-c were loaded on a separate gel. Loading control is not shown in the paper but is provided here.

Figure 1 H

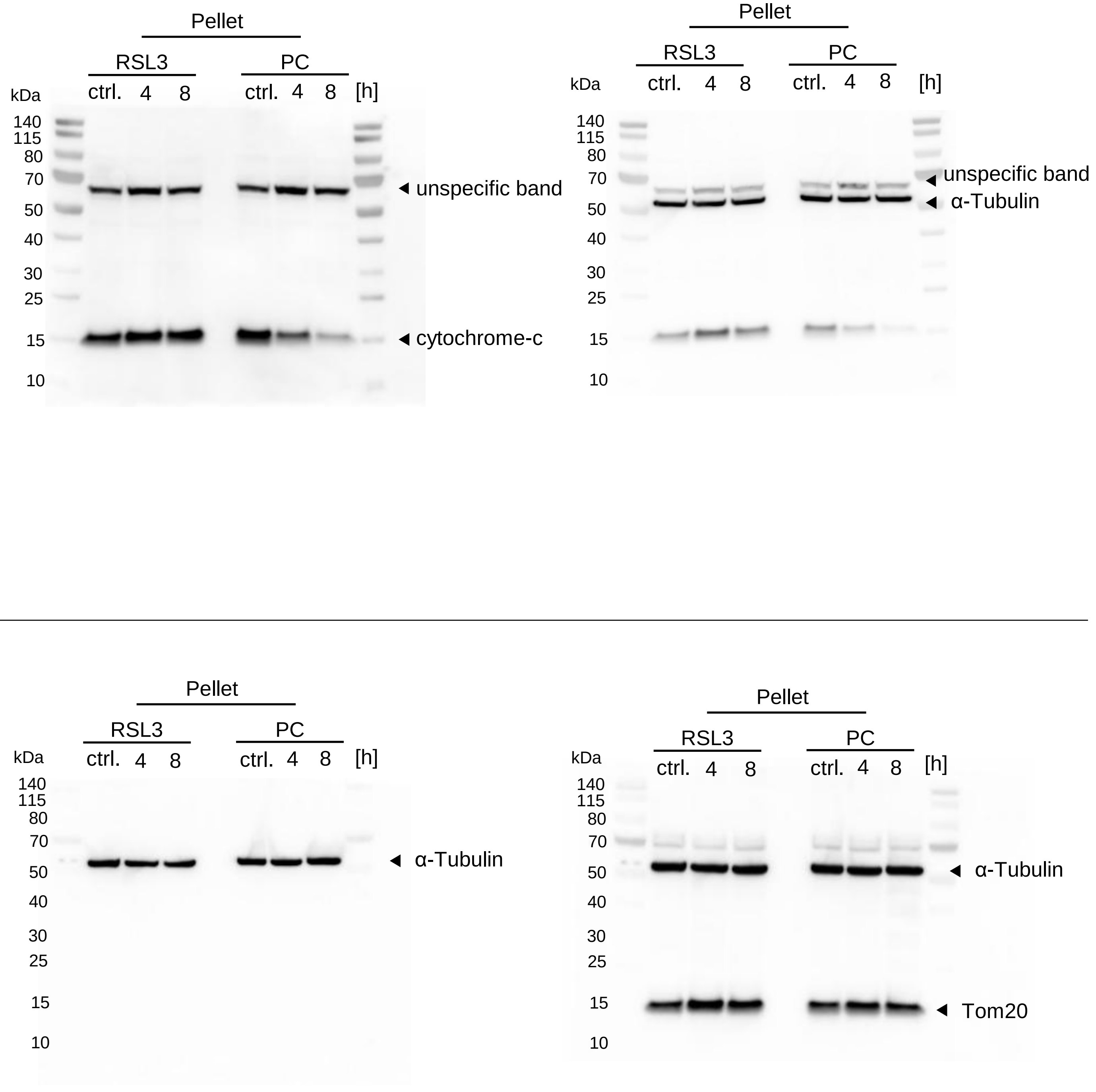

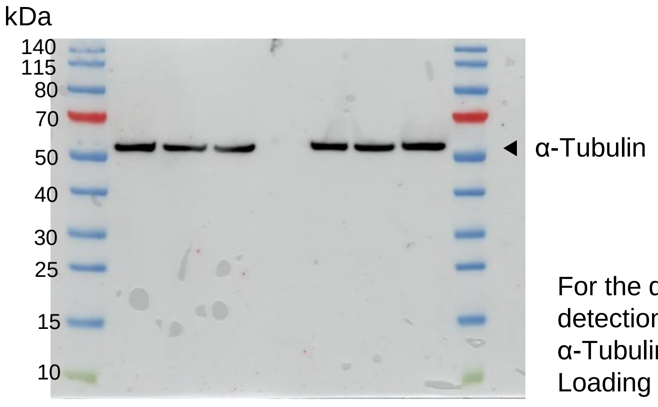

For the detection of Tom20, the same samples as for the detection of cytochrome-c were loaded on a separate gel. First α-Tubulin was detected then Tom20.
Loading control is not shown in the paper but is provided here.

Figure 1 I

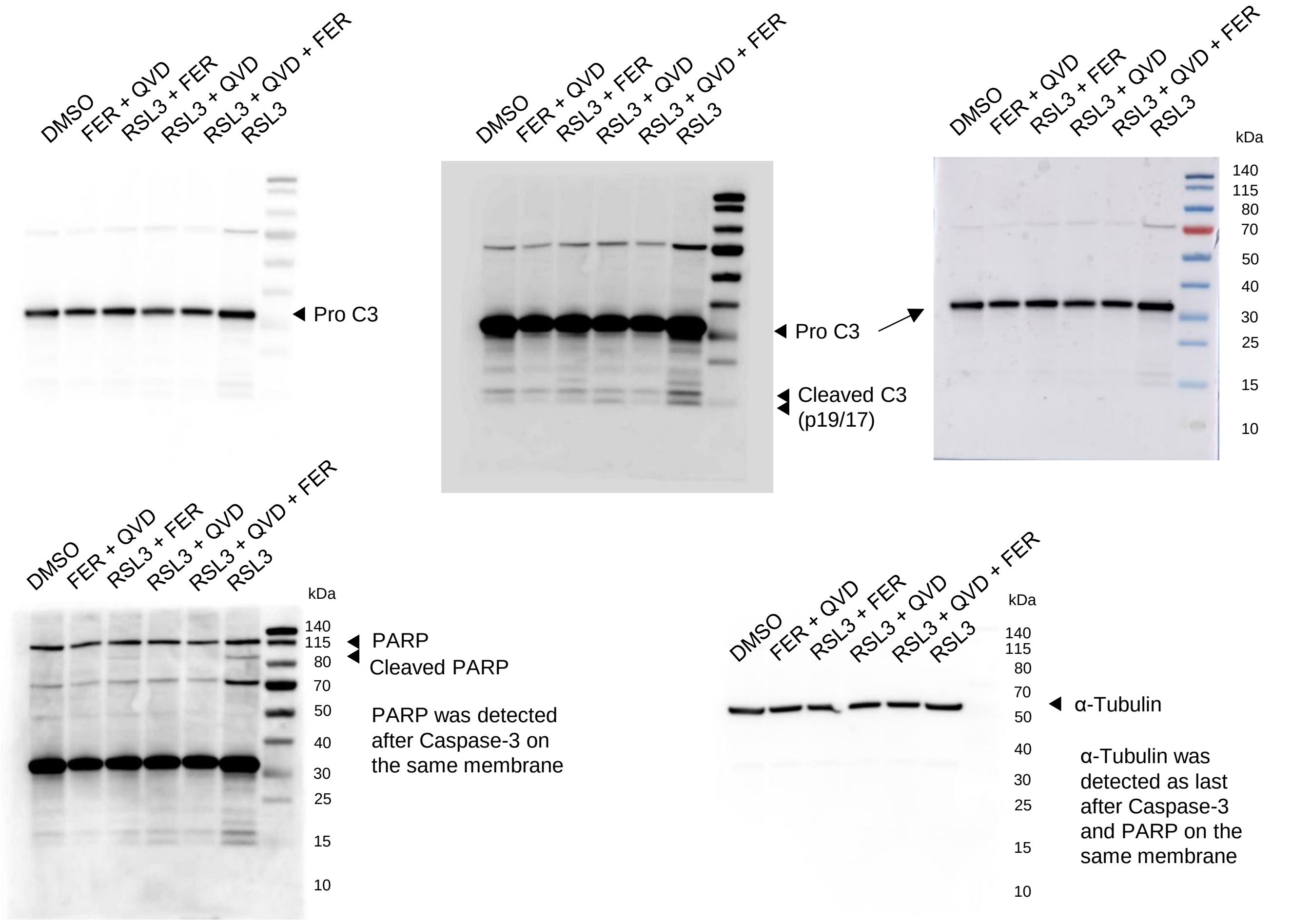

DMSO
FER + QVD
RSL3 + FER
RSL3 + QVD
RSL3 + QVD + FER
RSL3
Pro C3
Pro C3
Cleaved C3
(p19/17)
kDa
140
115
80
70
50
40
30
25
15
10
PARP
Cleaved PARP
PARP was detected after Caspase-3 on the same membrane
α-Tubulin
α-Tubulin was detected as last after Caspase-3 and PARP on the same membrane

Figure 1 J

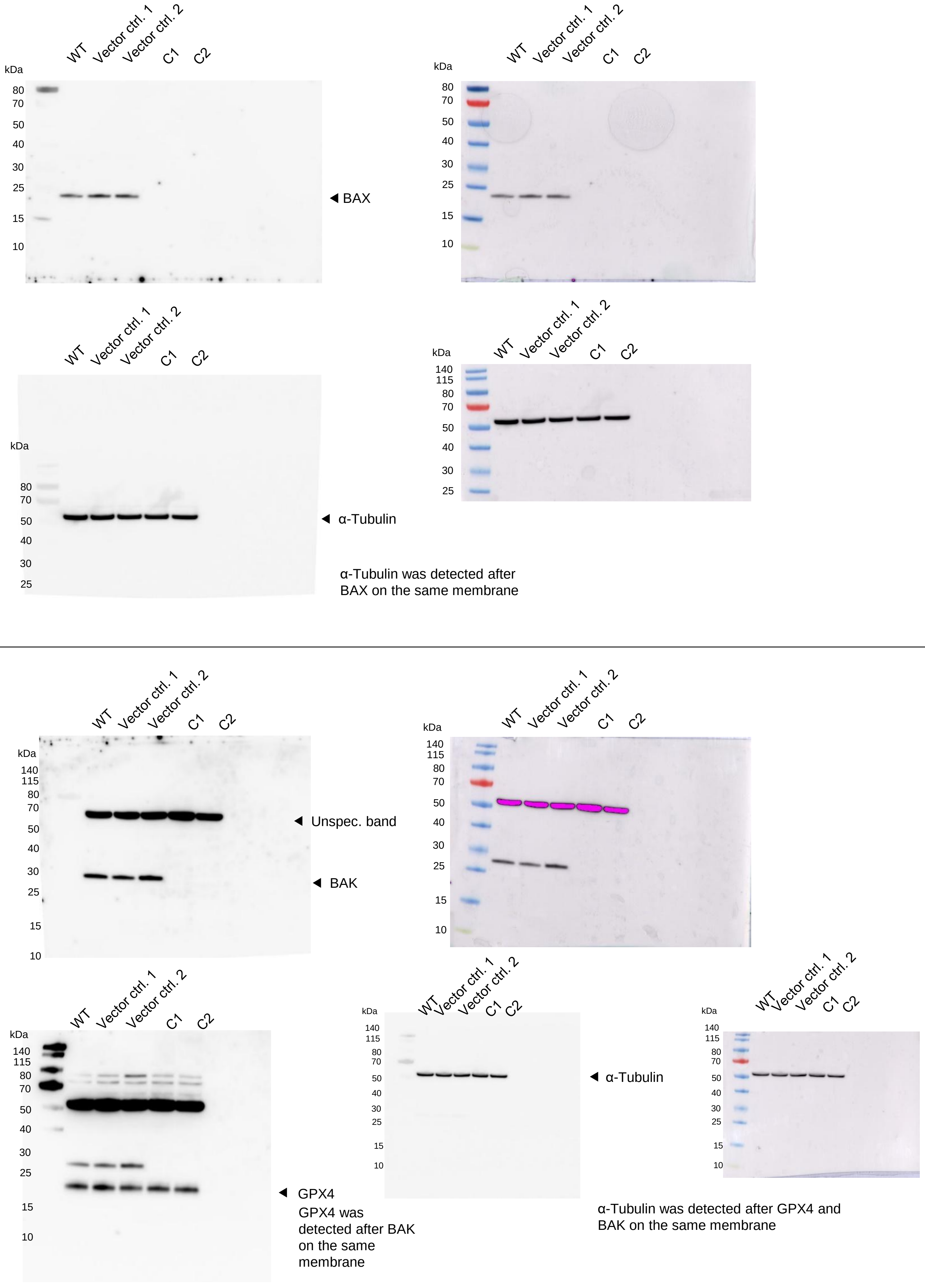
WT
Vector ctrl. 1
Vector ctrl. 2
C1
C2
kDa
80
70
50
40
30
25
15
10
BAX
kDa
140
115
80
70
50
40
30
25
α-Tubulin
α-Tubulin was detected after
BAX on the same membrane
Unspec. band
BAK
GPX4
GPX4 was
detected after BAK
on the same
membrane
α-Tubulin
α-Tubulin was detected after GPX4 and
BAK on the same membrane

Figure 5 B

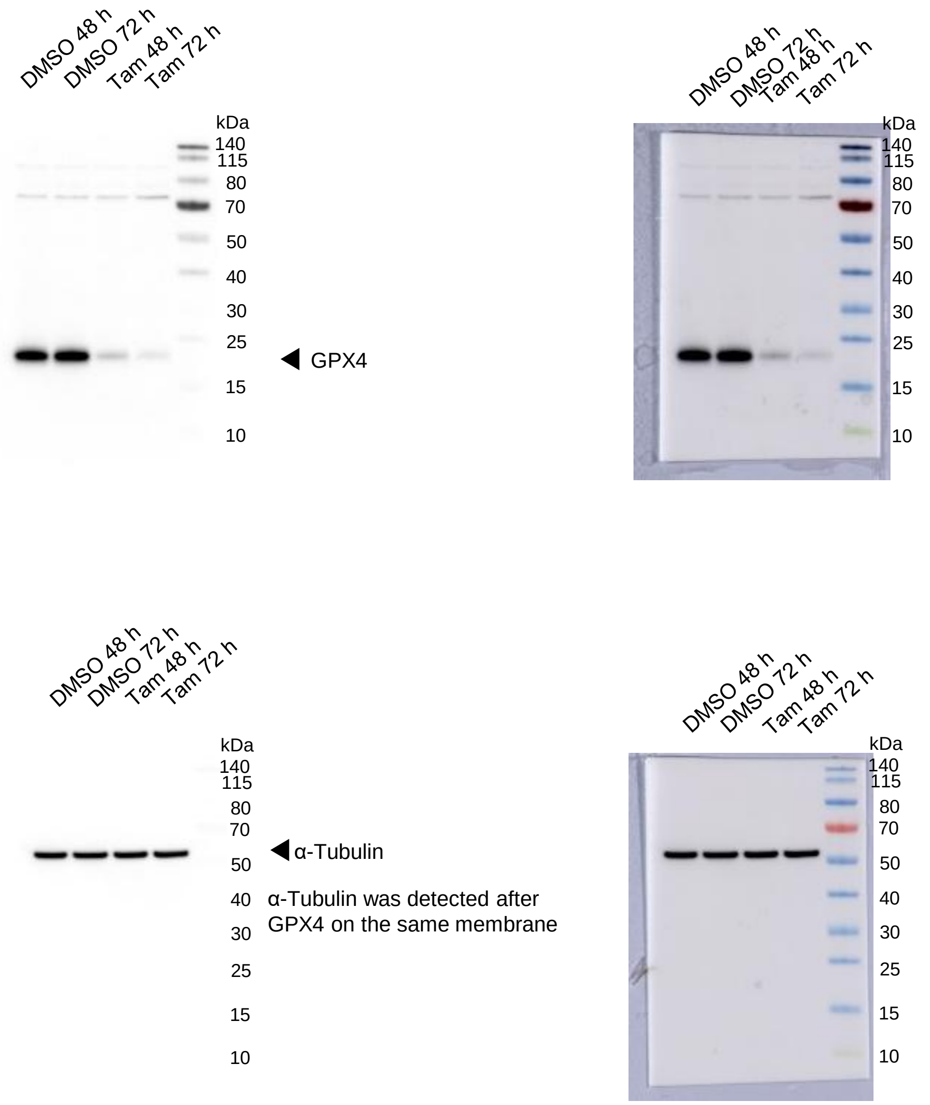
DMSO 48 h
DMSO 72 h
Tam 48 h
Tam 72 h
kDa
140
115
80
70
50
40
30
25
15
10
GPX4
α-Tubulin
α-Tubulin was detected after
GPX4 on the same membrane

Figure 5 C

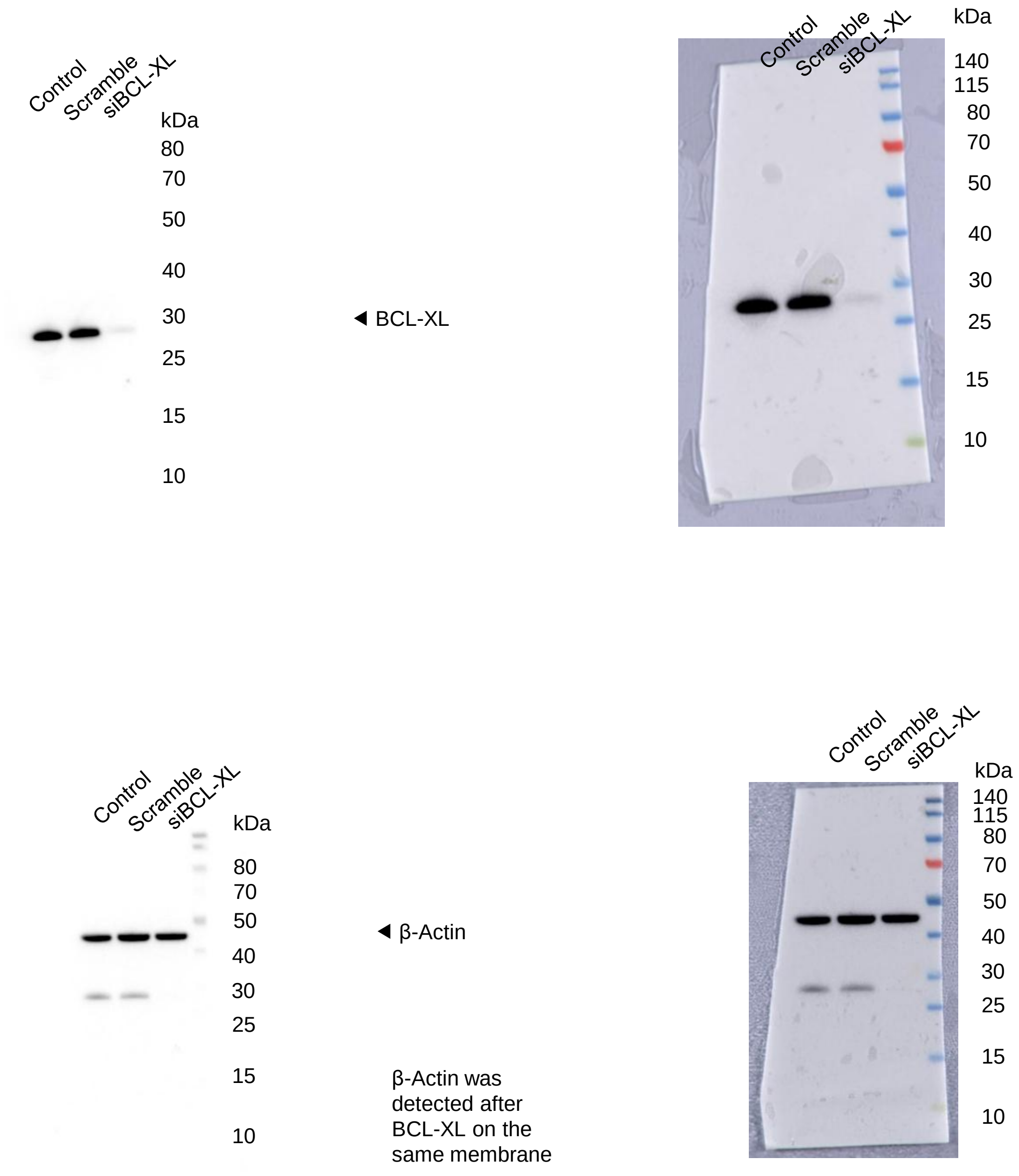
Control
Scramble
siBCL-XL
kDa
80
70
50
40
30
25
15
10
BCL-XL
Control
Scramble
siBCL-XL
kDa
140
115
80
70
50
40
30
25
15
10
Control
Scramble
siBCL-XL
kDa
80
70
50
40
30
25
15
10
β-Actin
β-Actin was detected after BCL-XL on the same membrane
Control
Scramble
siBCL-XL
kDa
140
115
80
70
50
40
30
25
15
10

Figure 5 D

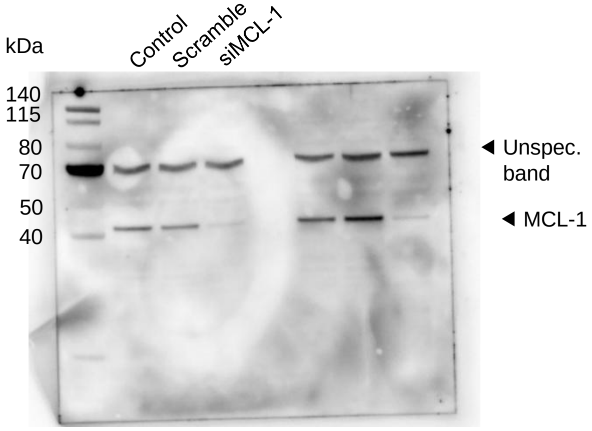

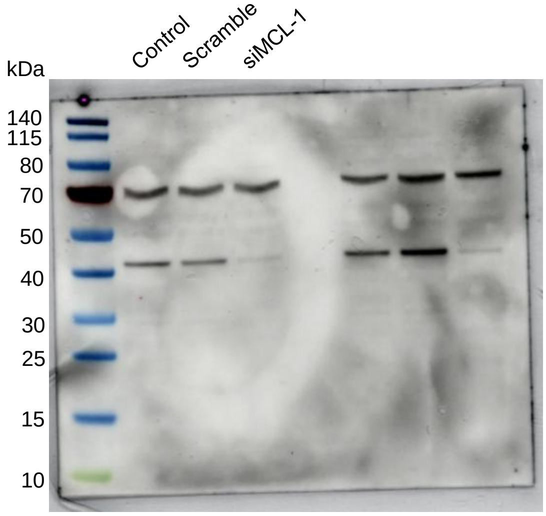

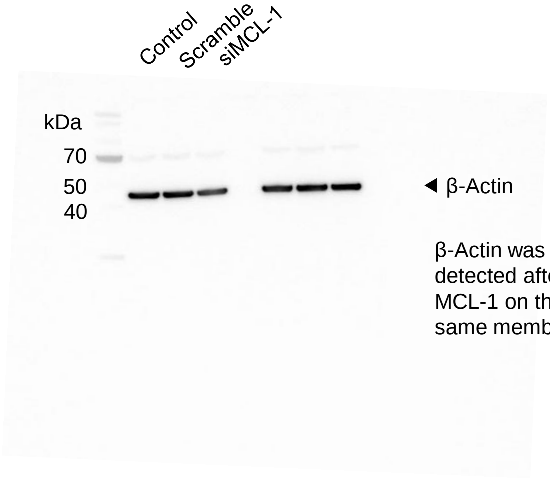

β-Actin was detected after MCL-1 on the same membrane

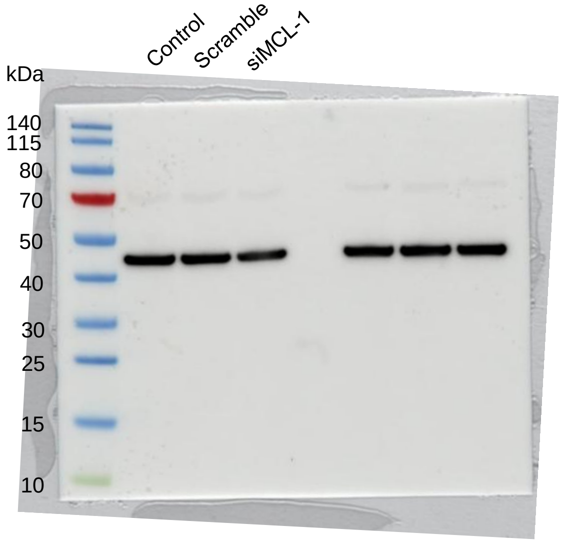

Supplemental Figure 3 G

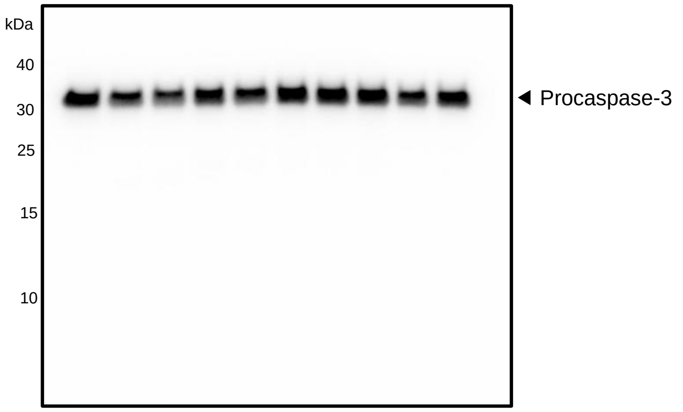

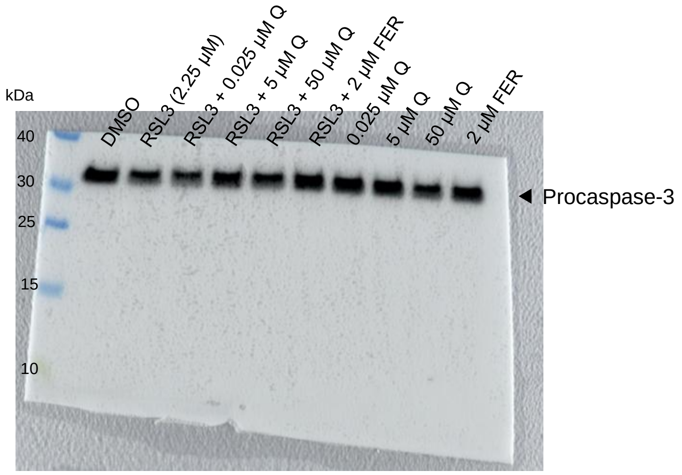

Membrane was cut

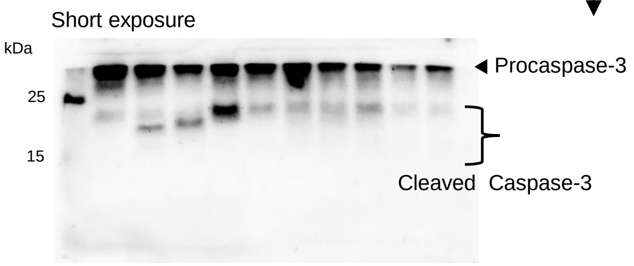

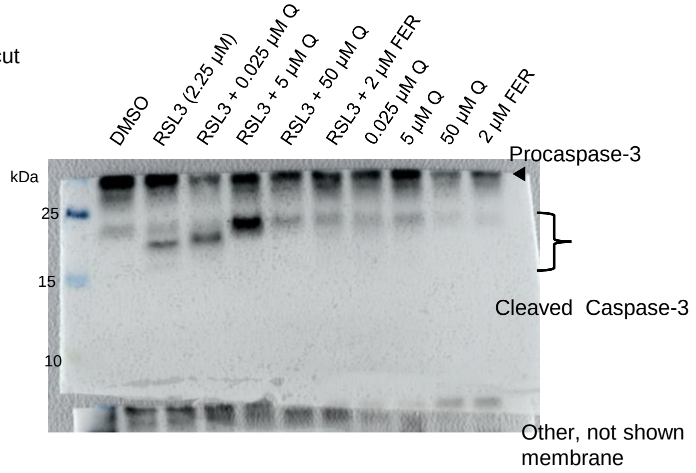

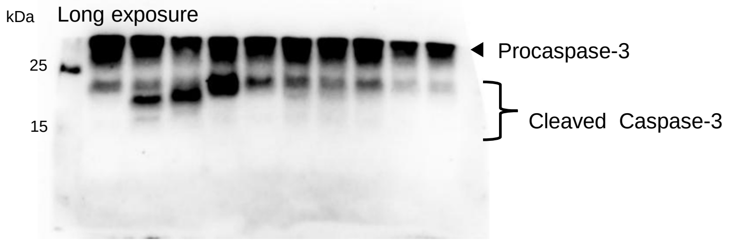

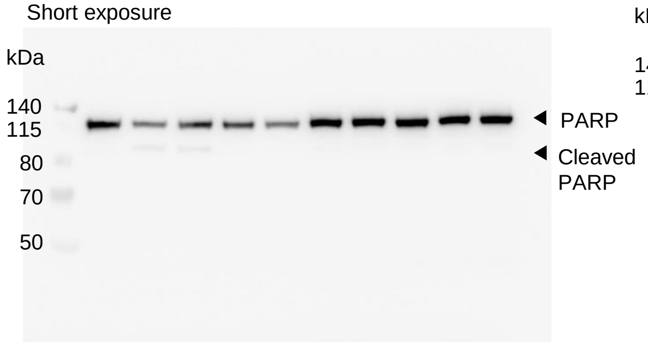

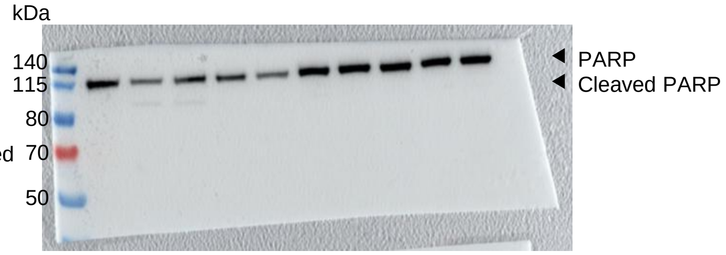

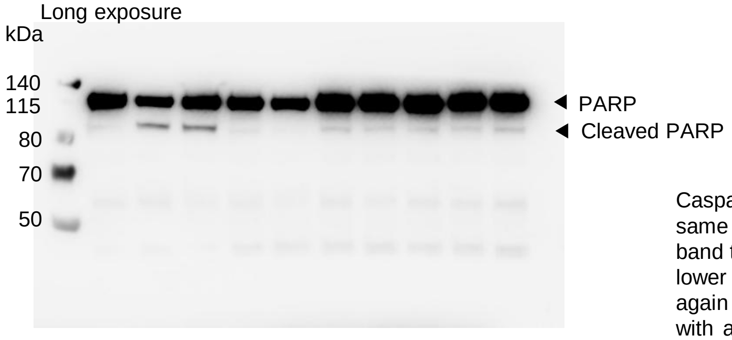

Caspase-3, PARP and α-Tubulin were all detected on the same membrane. First, membrane was cut at the 40 kDa band to detect PARP on the upper part and caspase-3 on the lower part of the membrane. Then the lower part was cut again to remove the procaspase-3 band and incubate again with anti-caspase-3 to better detect the cleavage fragments of caspase-3. α-Tubulin (next page) was detected on the upper part of the membrane were before PARP was detected.

Supplemental Figure 3 G

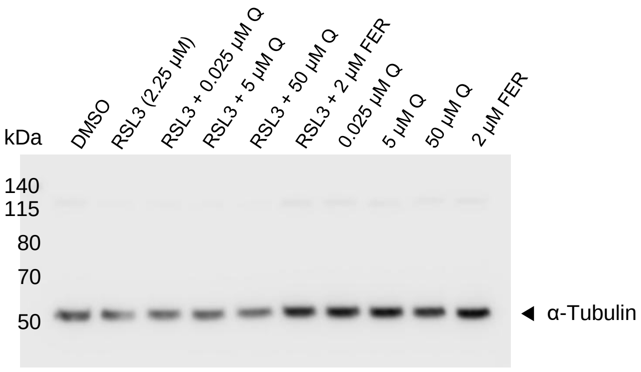

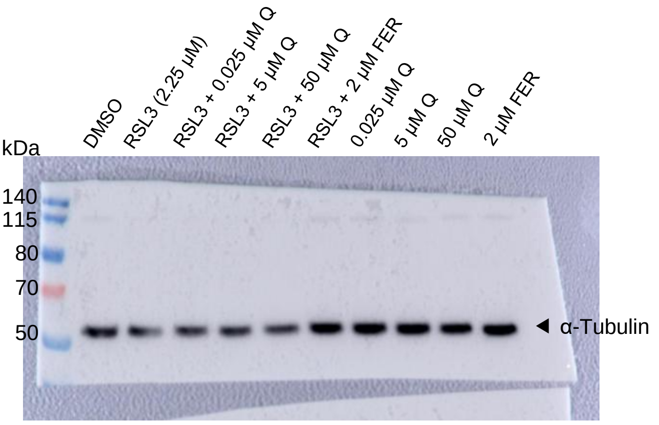

Supplemental Figure 5 A

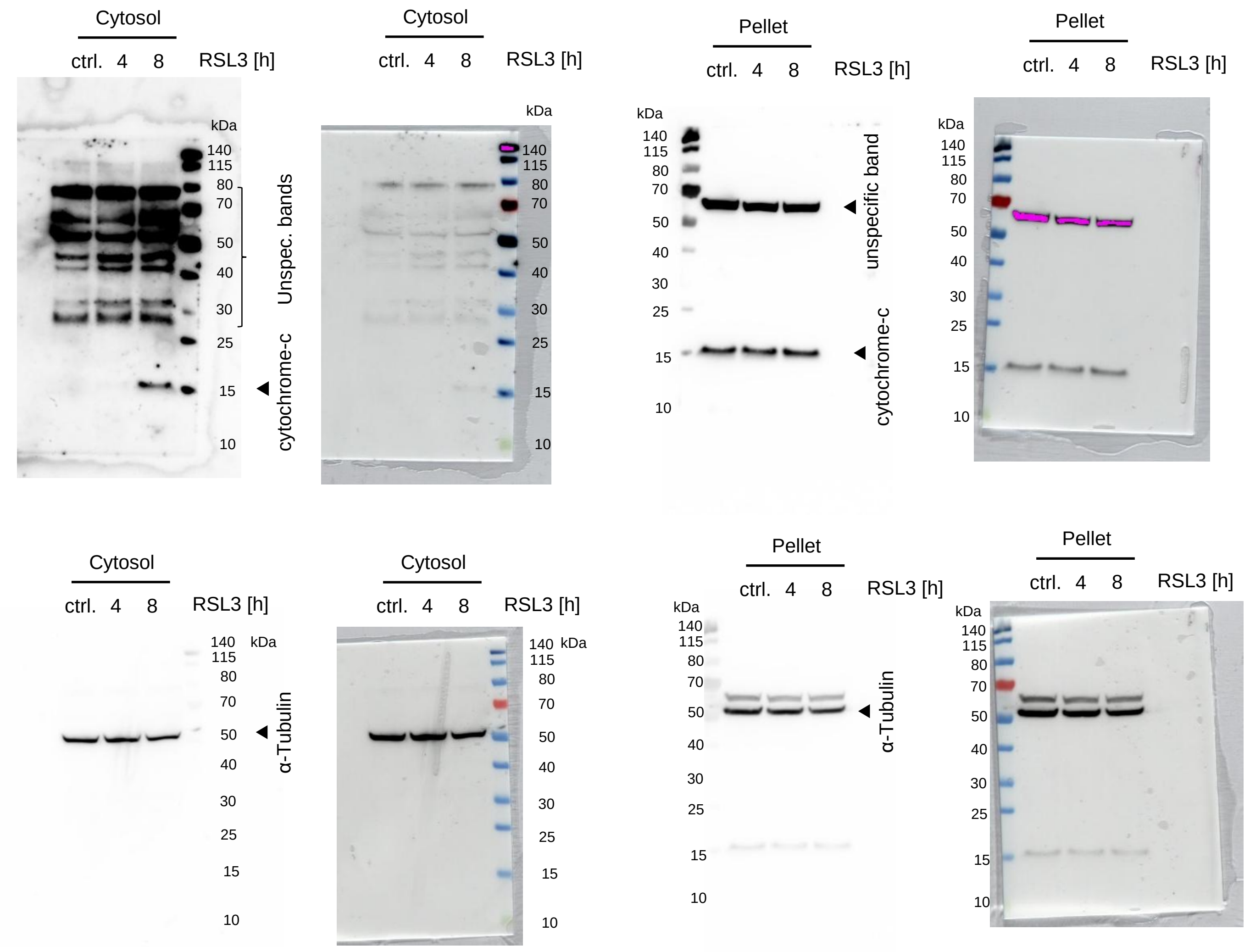

Supplemental Figure 5 A

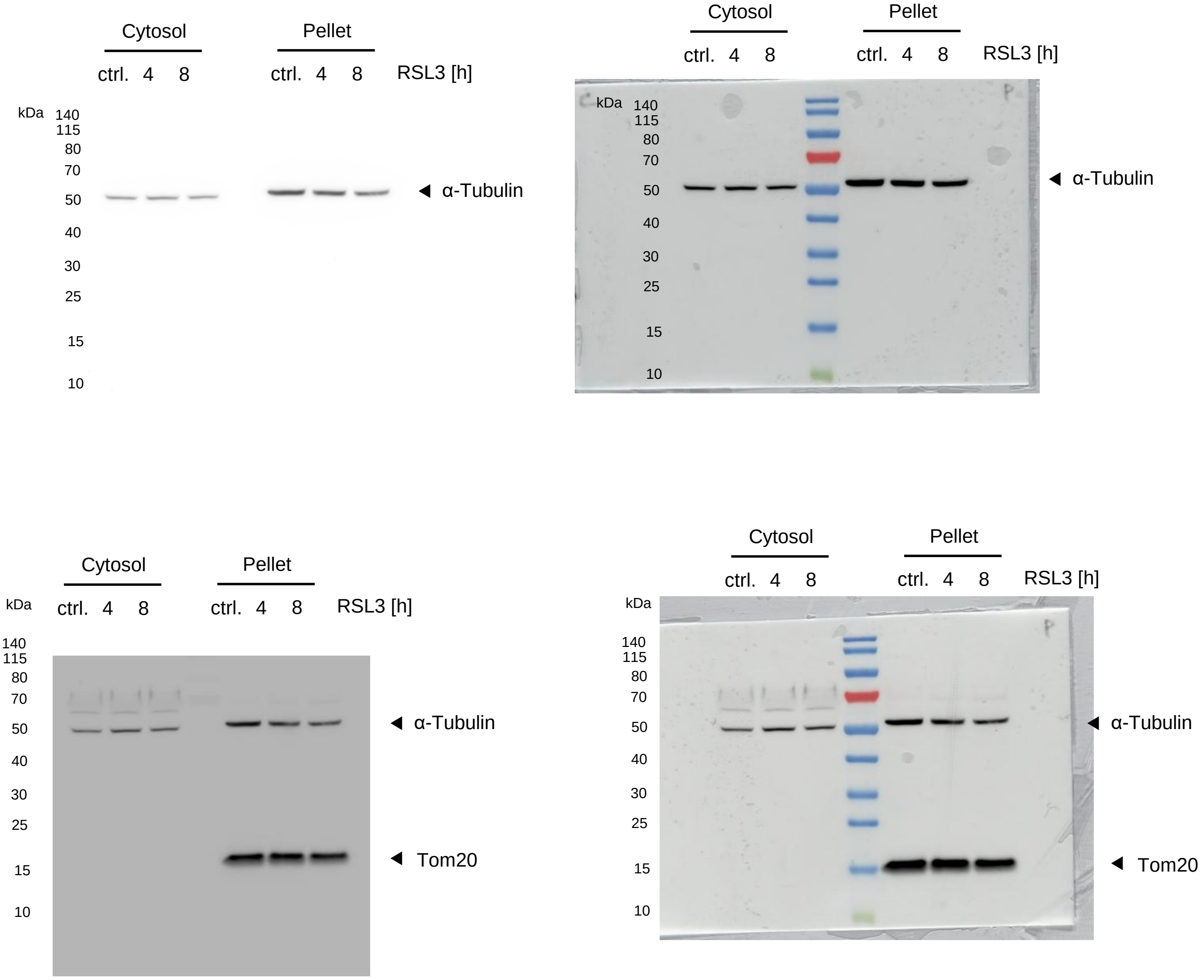

For the detection of Tom20, the same samples as for the detection of cytochrome-c were loaded on a separate gel. First α-Tubulin was detected then Tom20.
Loading control is not shown in the paper but is provided here.

Supplemental Figure 5 B

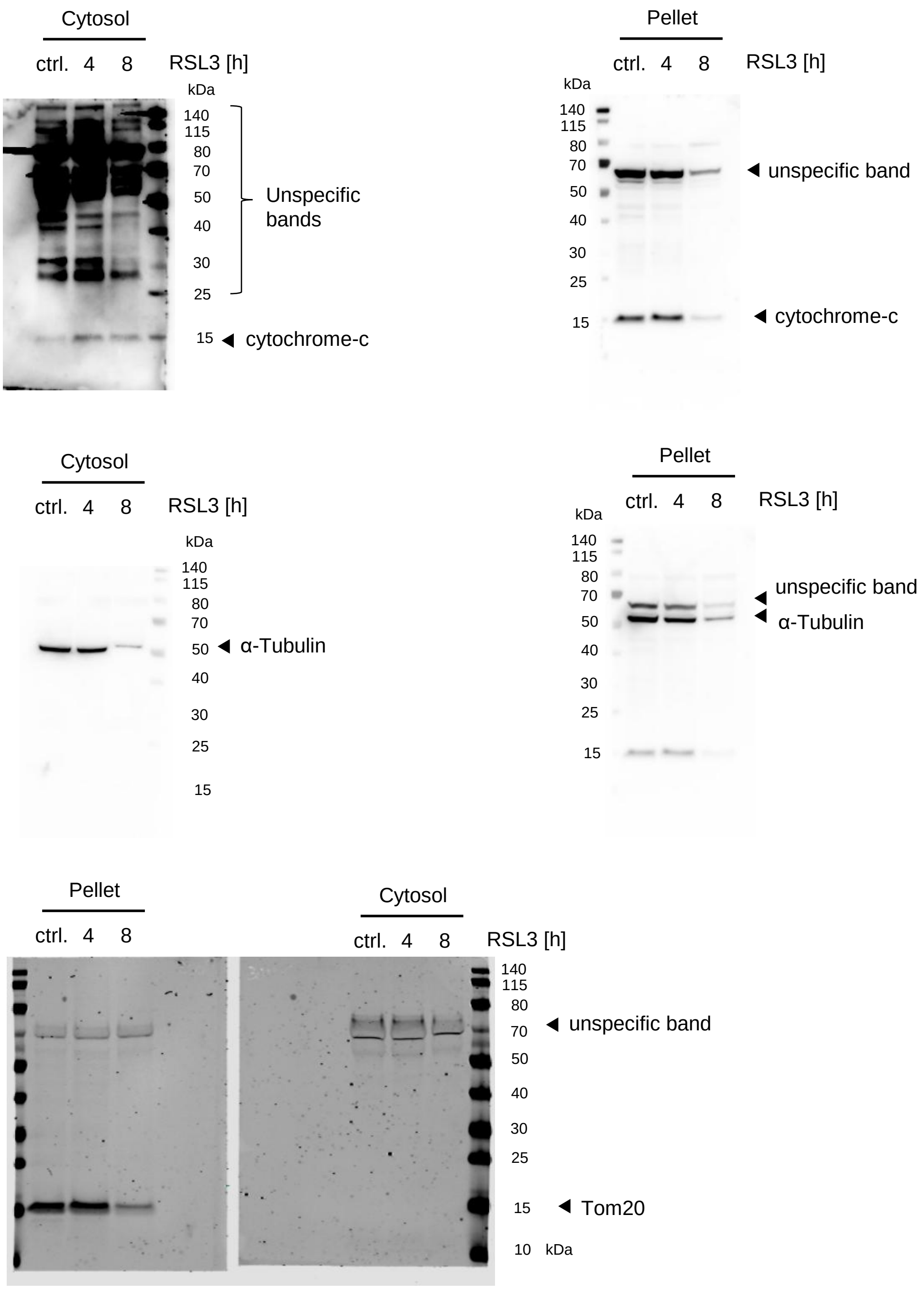

Tom20 was detected after cytochrome-c and α-Tubulin on the same membrane using a fluorescently-labelled secondary antibody and the LI-COR system.

Supplemental Figure 5 C

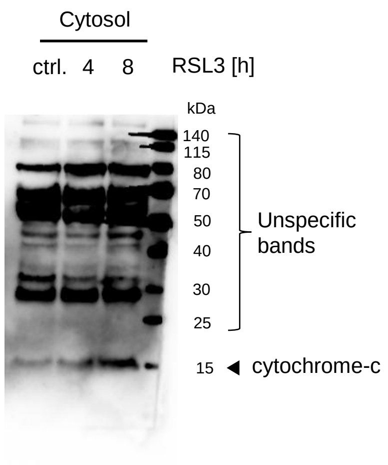

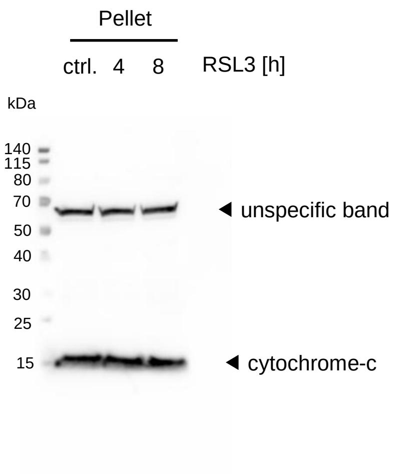

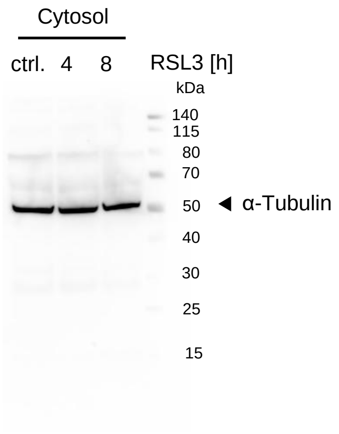

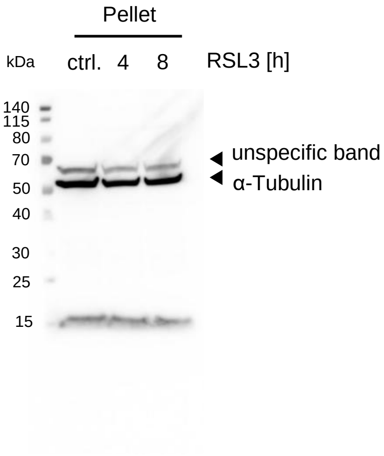

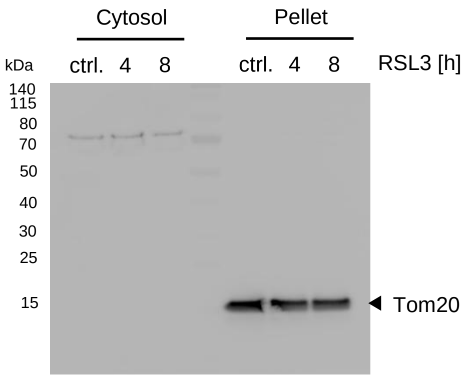

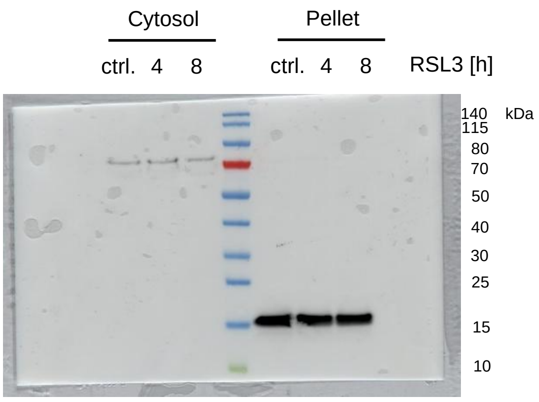

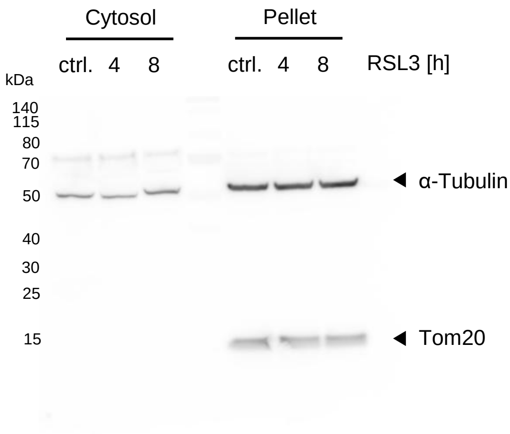

Samples for detection of Tom20 are the same as for cytochrome-c but they were loaded on a separate gel. Loading control for this membrane is not shown in the paper but is provided here.

Supplemental Figure 5 D

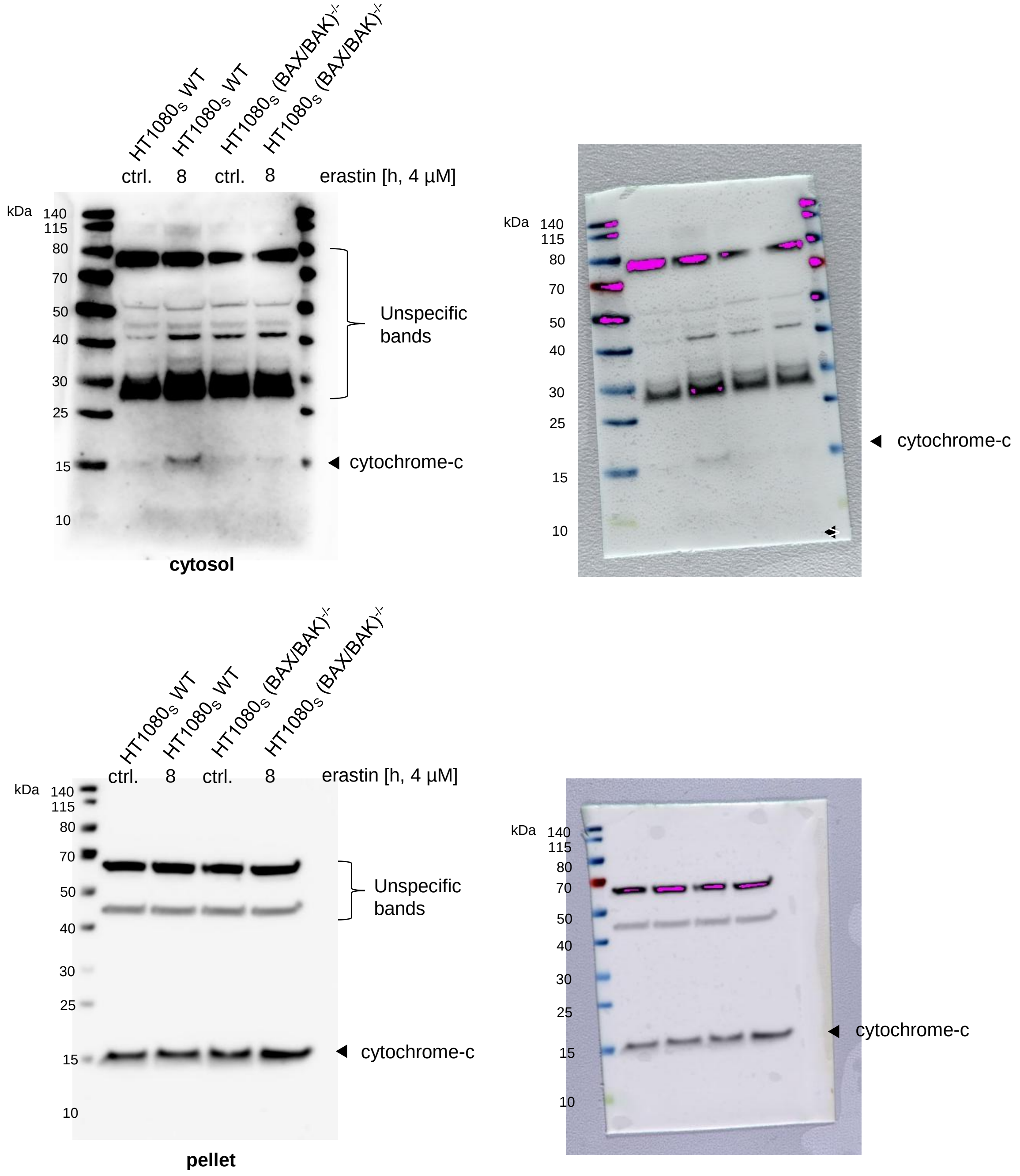

Supplemental Figure 5 D

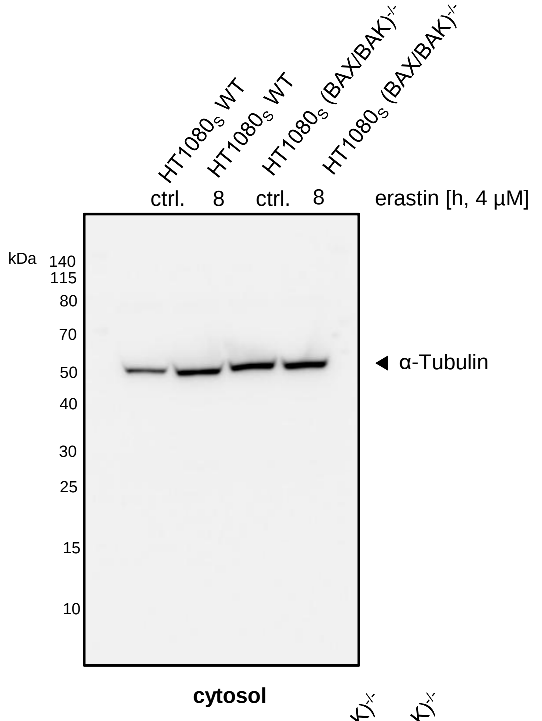

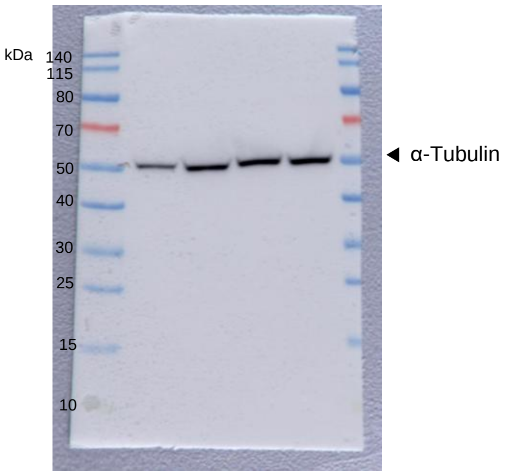

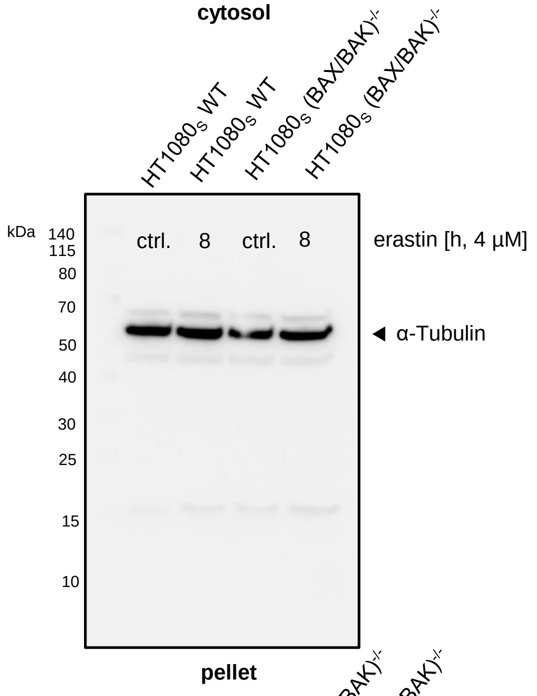

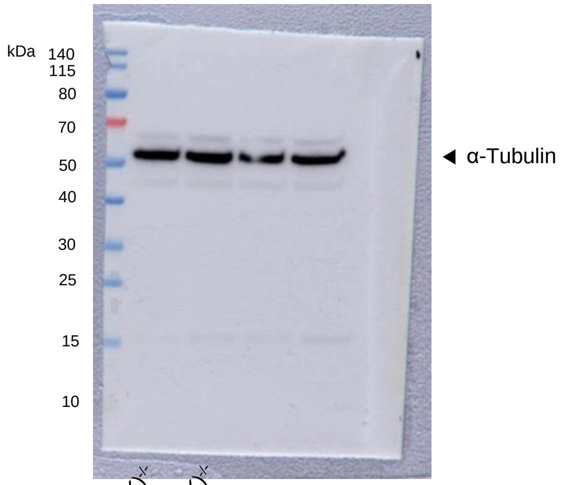

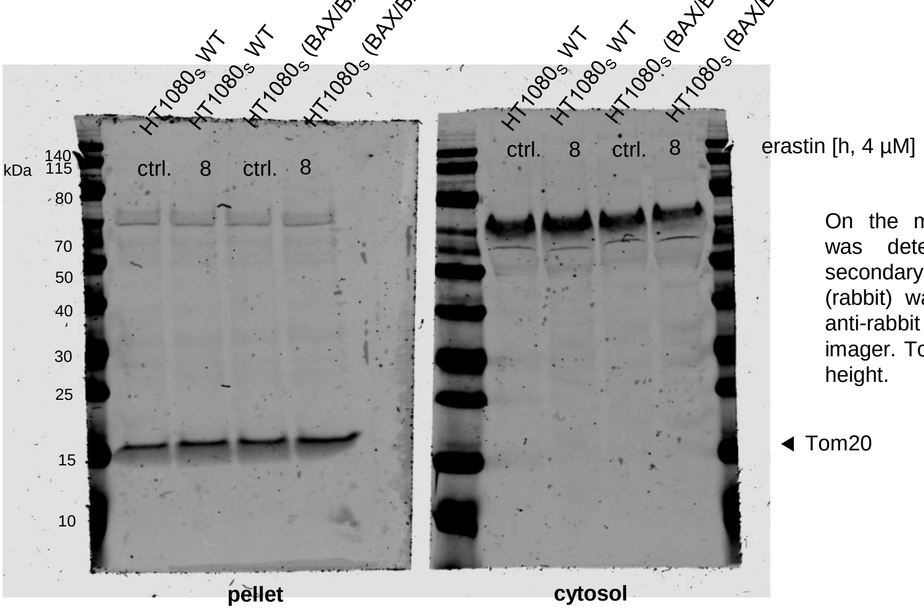

On the membranes, anti-cytochrome-c (mouse) was detected first, using an HRP-coupled secondary anti-mouse antibody. Then anti-Tom20 (rabbit) was detected with a fluorescently-labled anti-rabbit secondayry antibody and the LI-COR imager. Tom20 and cytochrome-c run at the same height.

Supplemental Figure 6 A

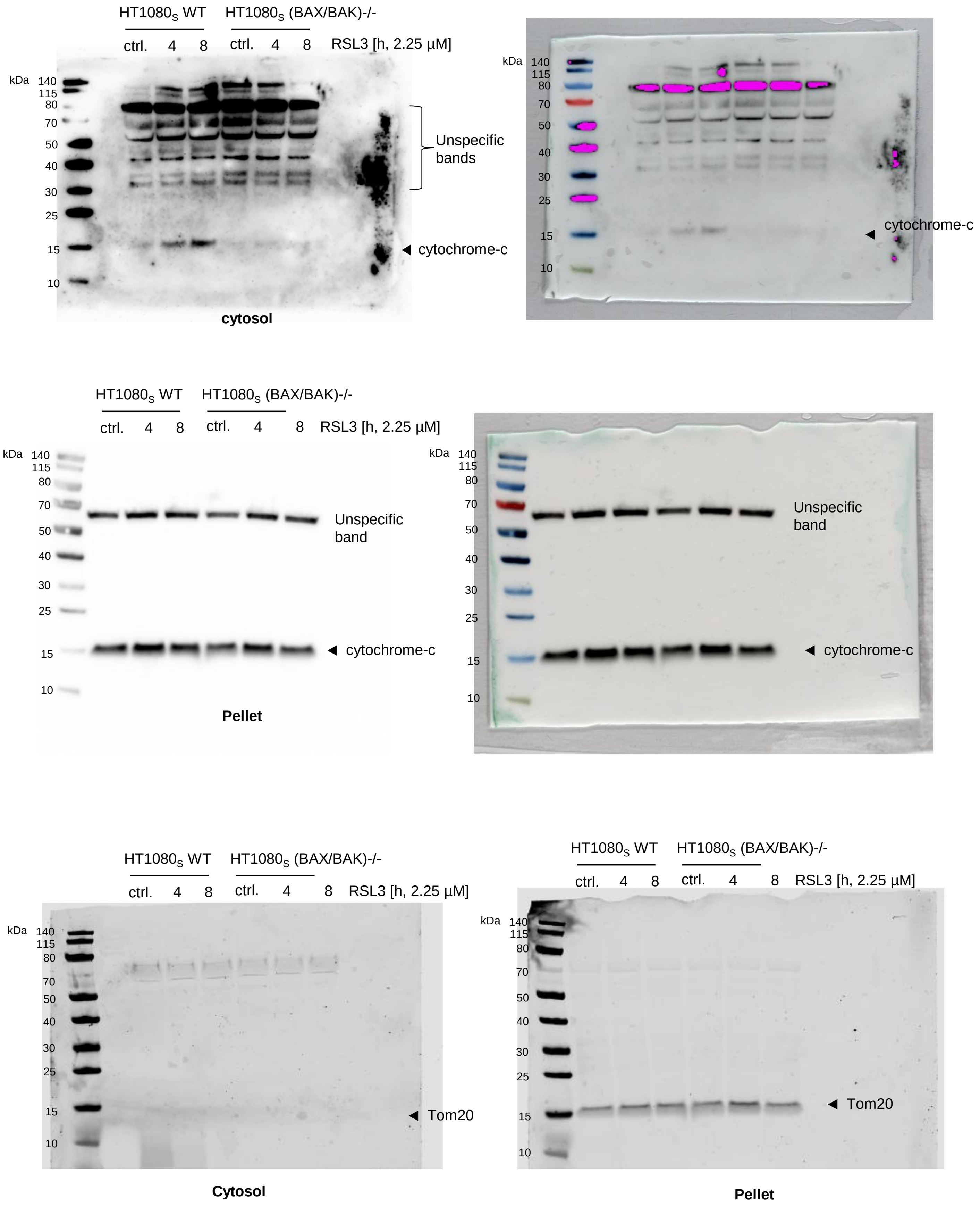

On the membranes, anti-cytochrome-c (mouse) was detected first, using an HRP-coupled secondary anti-mouse antibody. Then anti-Tom20 (rabbit) was detected with a fluorescently-labled anti-rabbit secondayry antibody and the LI-COR imager. Tom20 and cytochrome-c run at the same height.

Supplemental Figure 6 A

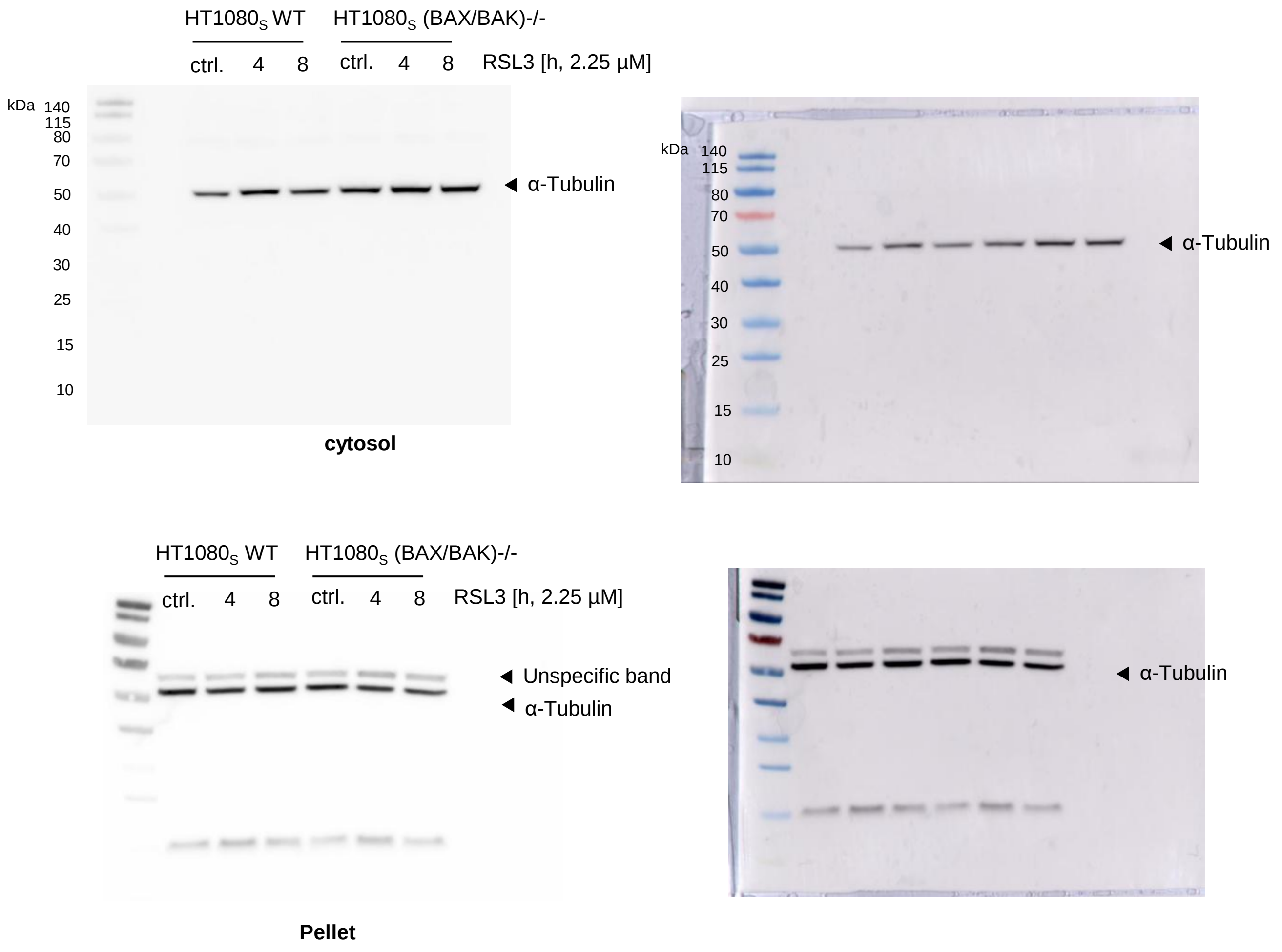

Supplemental Figure 6 B

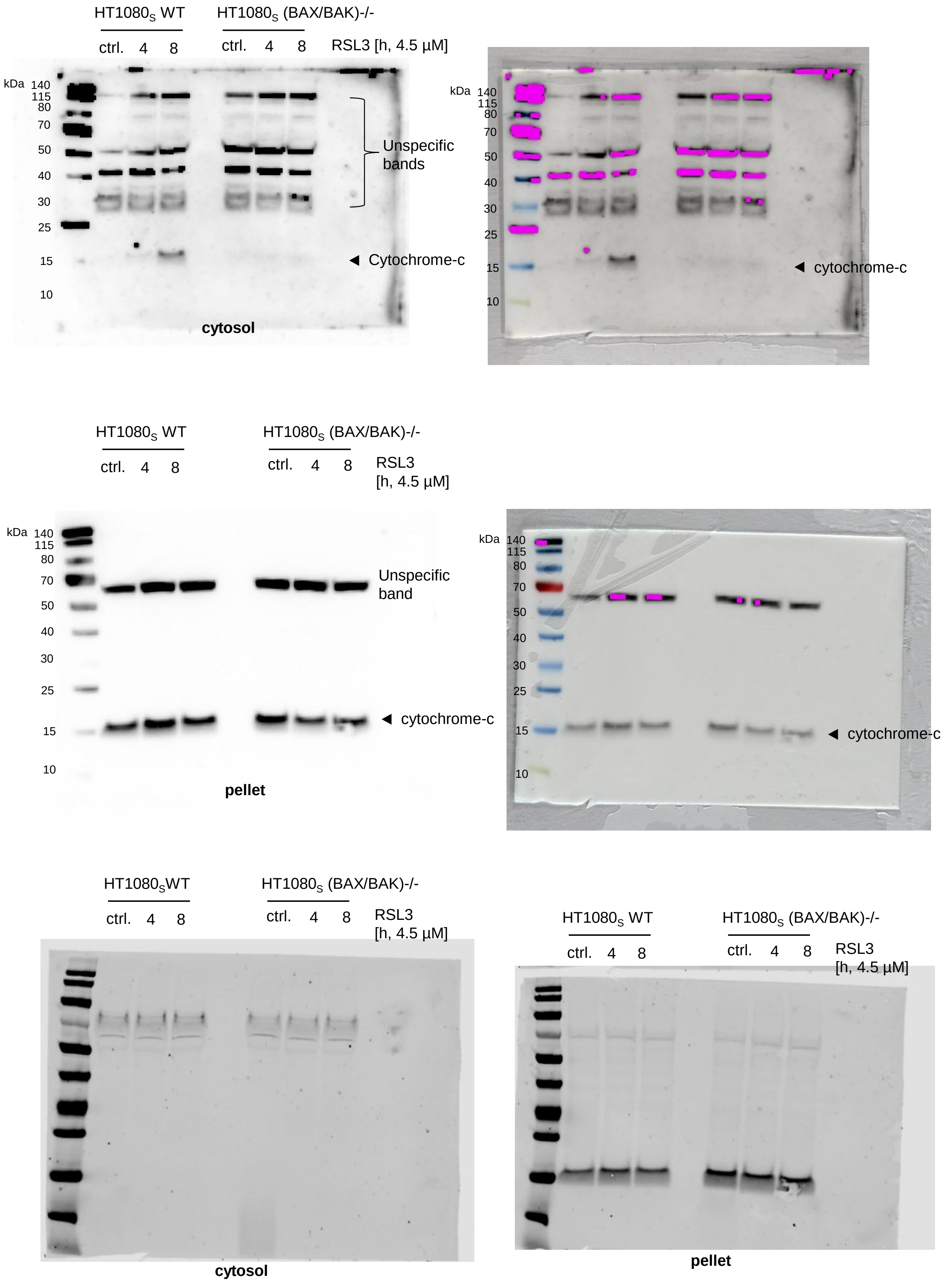

On the membranes, anti-cytochrome-c (mouse) was detected first, using an HRP-coupled secondary anti-mouse antibody. Then anti-Tom20 (rabbit) was detected with a fluorescently labled anti-rabbit secondayry antibody and the LI-COR imager. Tom20 and cytochrome-c run at the same height.

Supplemental Figure 6 B

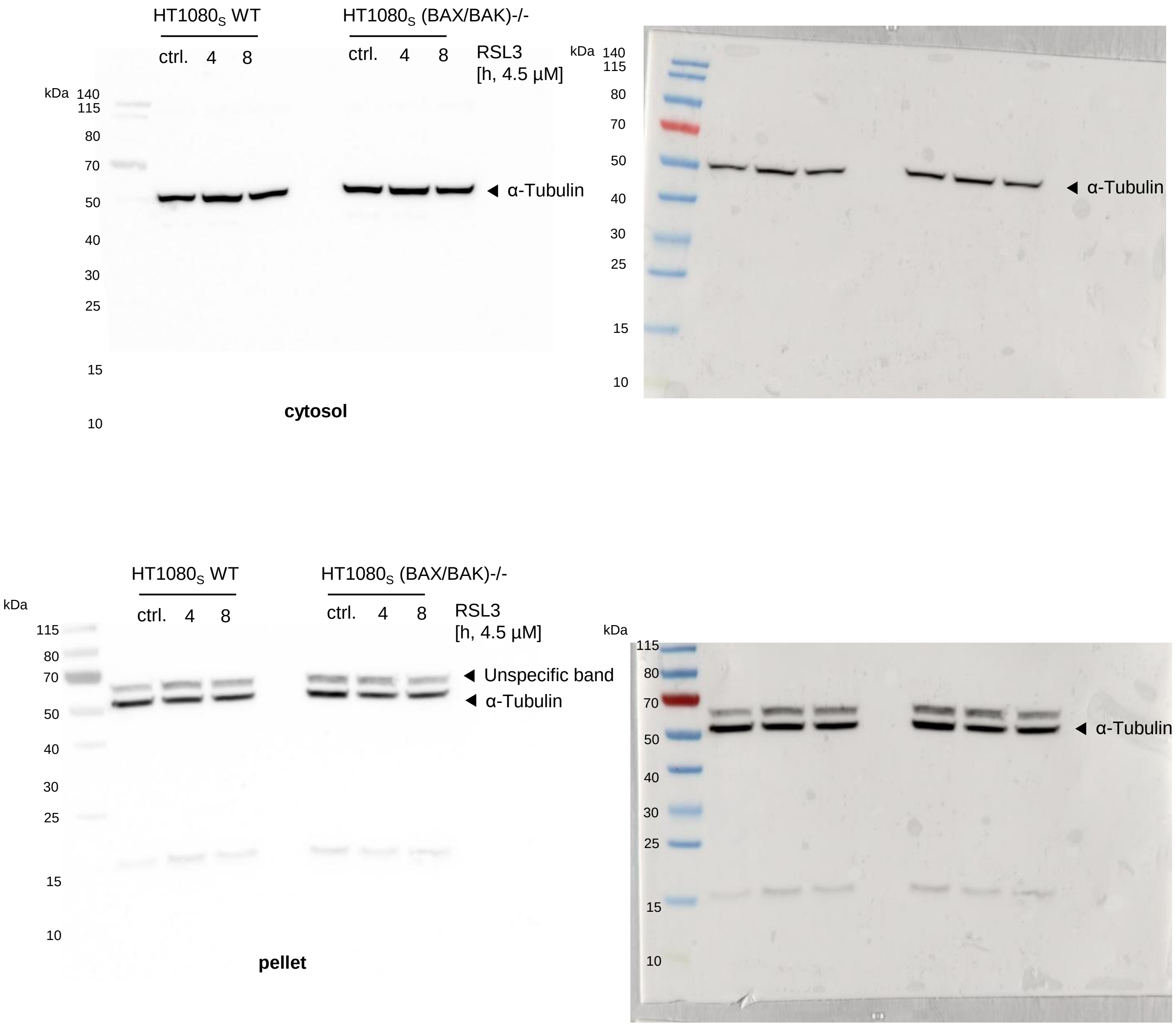

Supplemental Figure 6 C

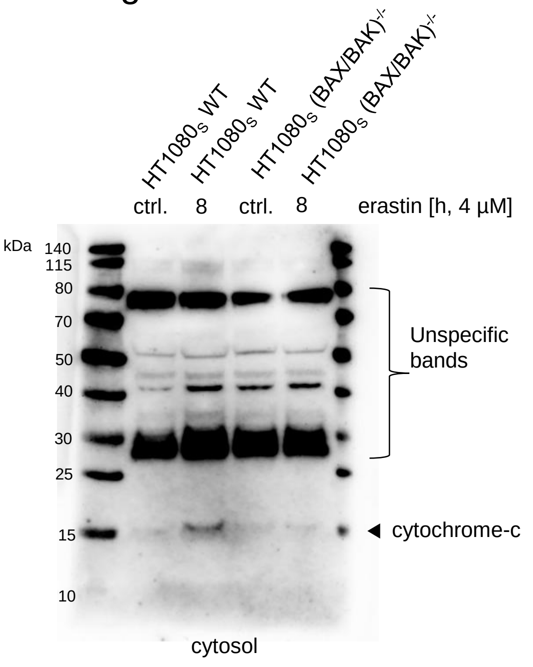

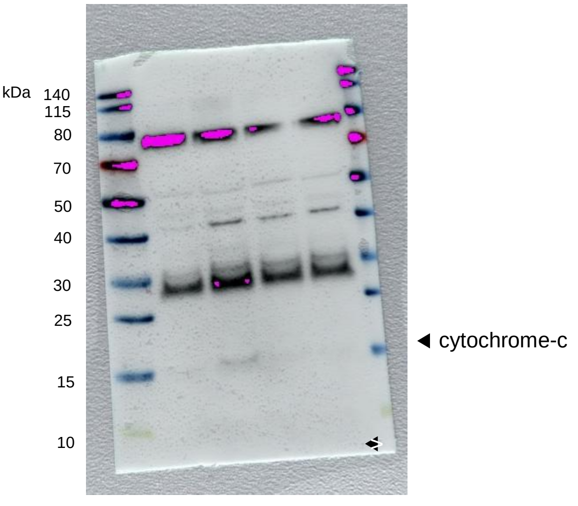

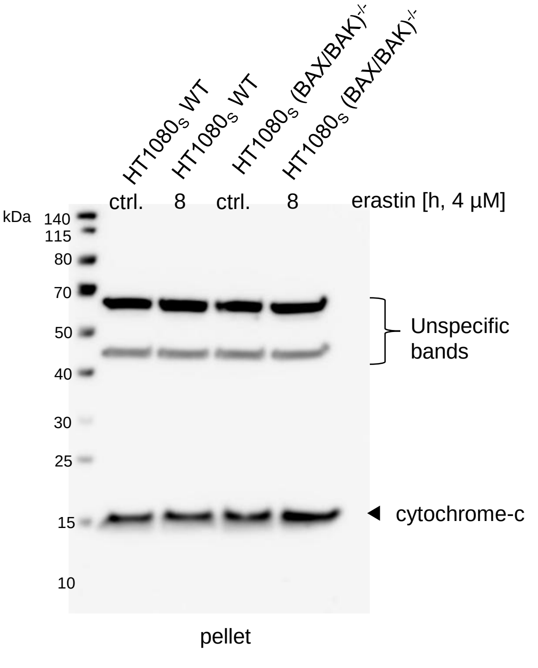

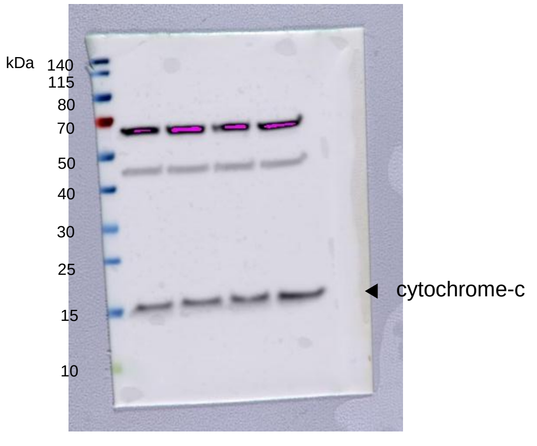

Supplemental Figure 6 C

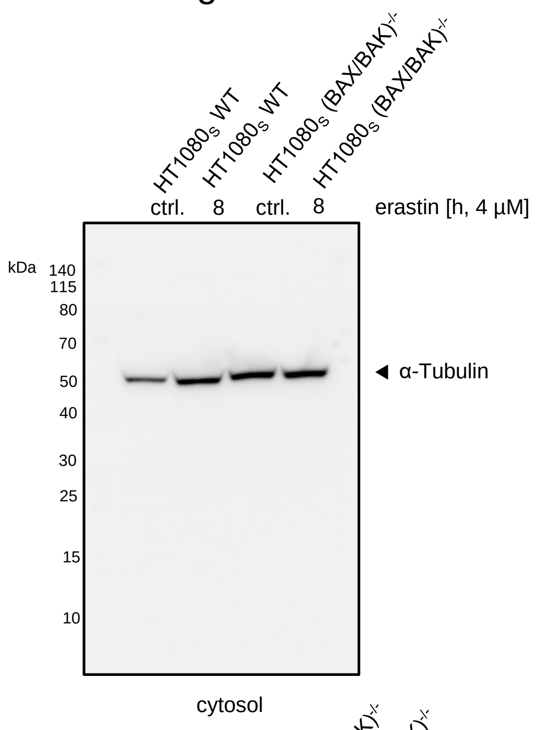

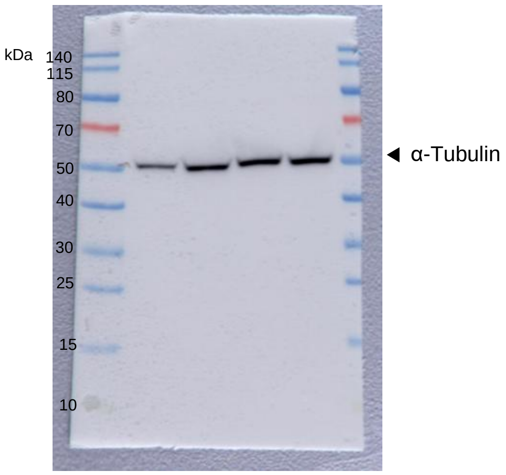

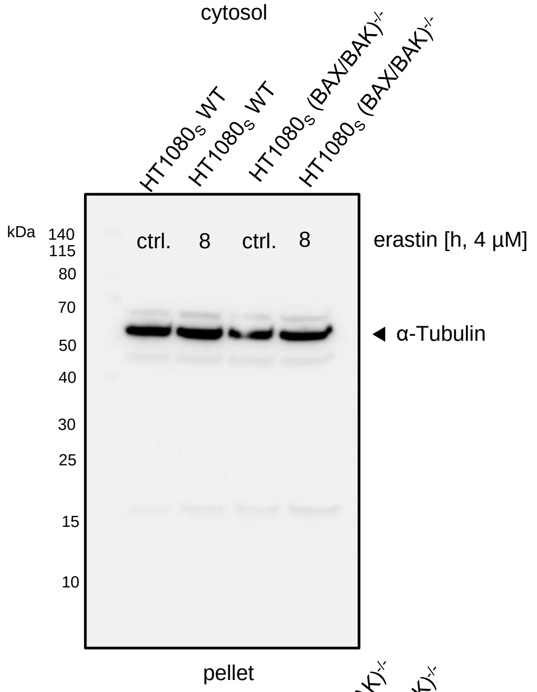

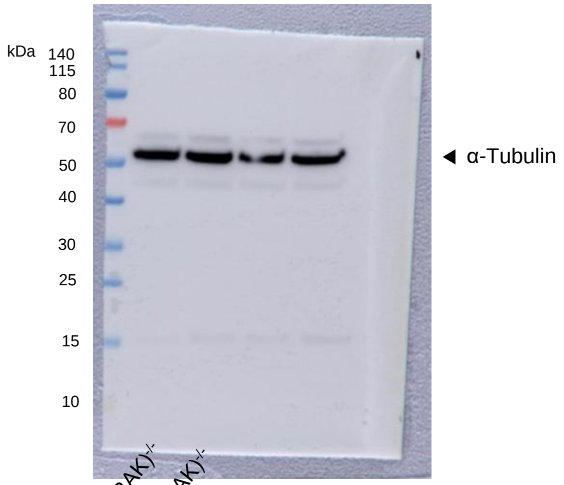

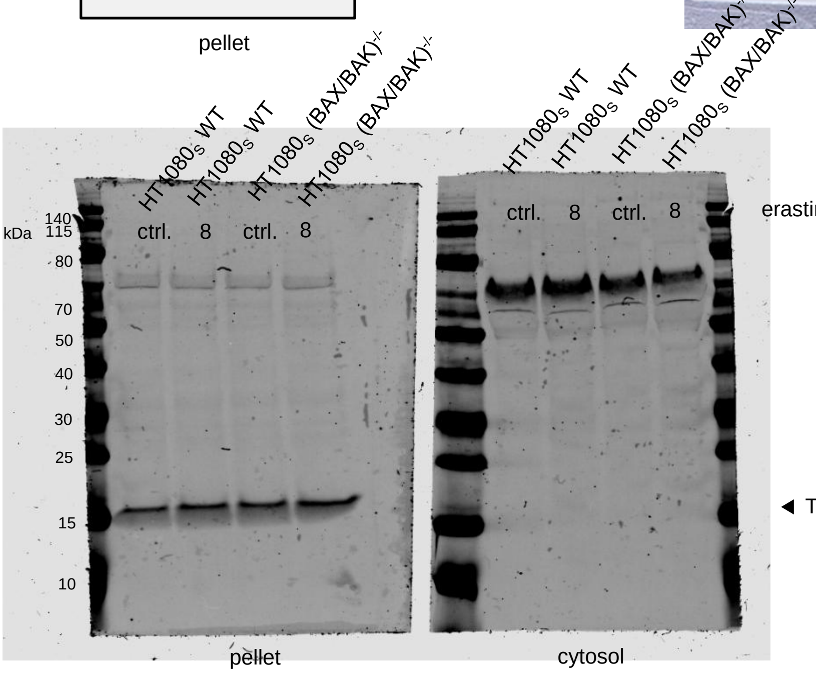

On the membranes, anti-cytochrome-c (mouse) was detected first, using an HRP-coupled secondary anti-mouse antibody. Then anti-Tom20 (rabbit) was detected with a fluorescently labled anti-rabbit secondayry antibody and the LI-COR imager. Tom20 and cytochrome-c run at the same height.

Supplemental Figure 6 D

First caspase-3 was detected, afterwards PARP was detecetd then α-Tubulin was detected on the same membrane.

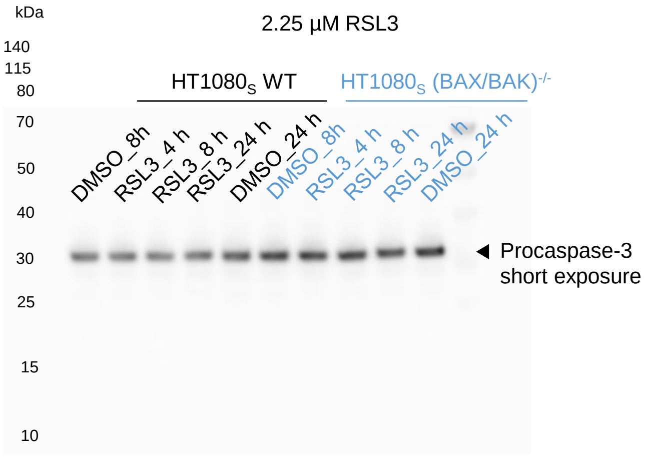

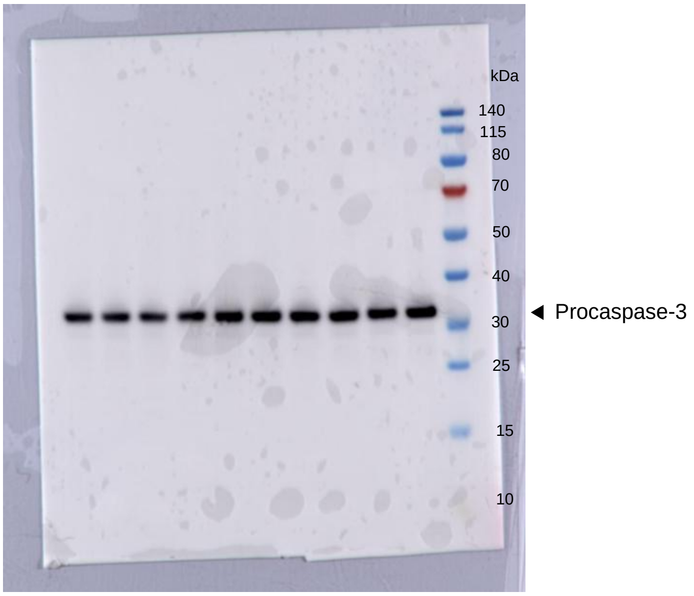

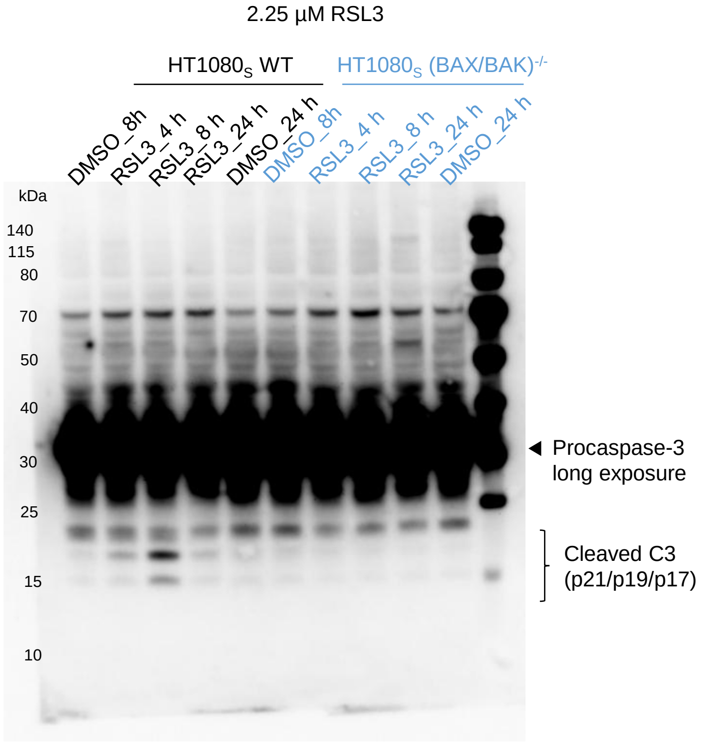

Supplemental Figure 6 D

First caspase-3 was detected, afterwards PARP was detecetd then α-Tubulin was detected on the same membrane.

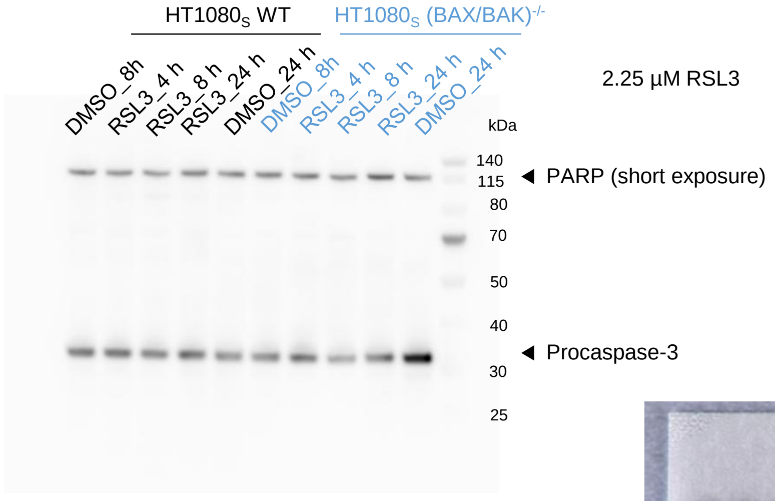

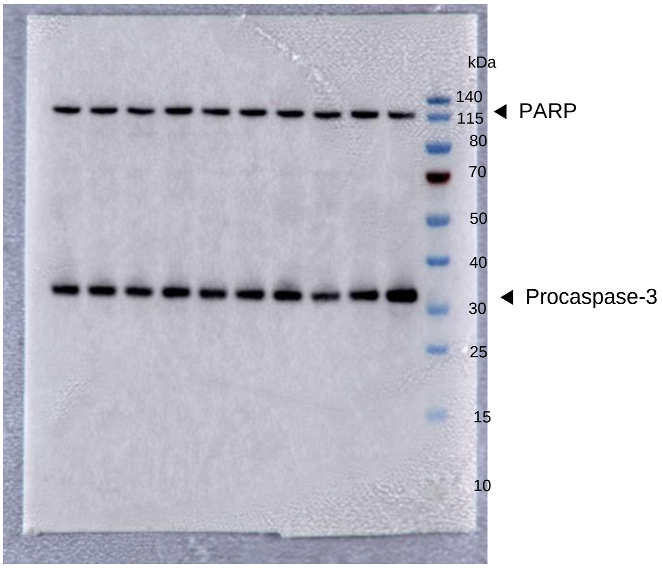

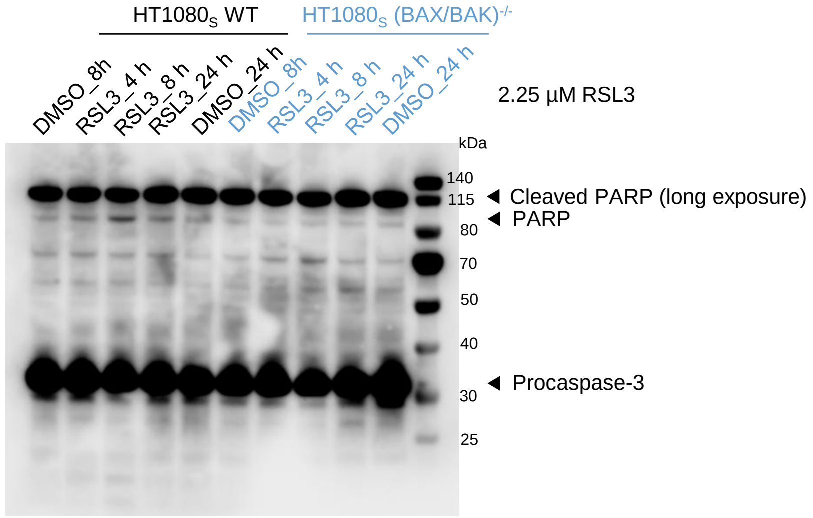

Supplemental Figure 6 D

Lastely, α-Tubulin was detected on the same membrane

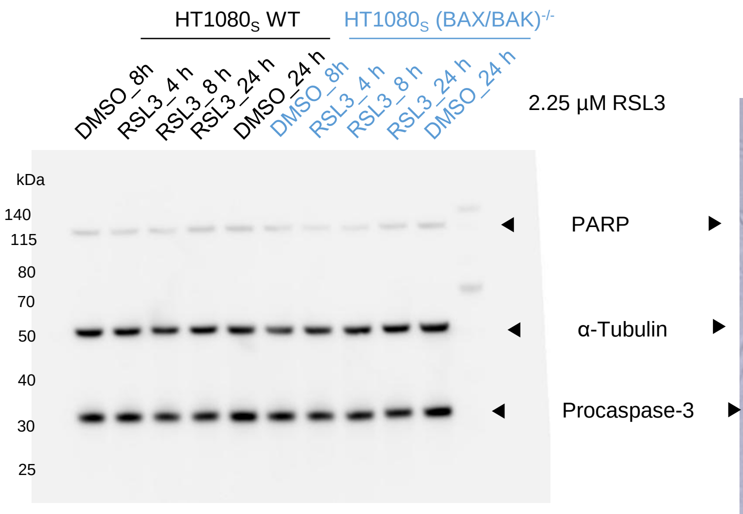

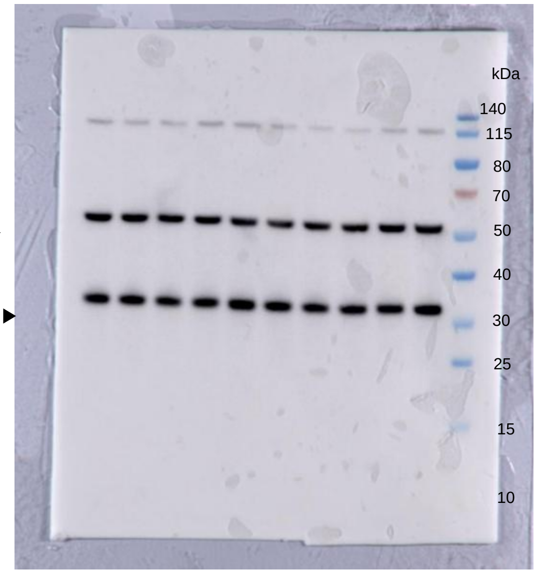

Supplemental Figure 6 E

First caspase-3 was detected, afterwards PARP was detecetd then α-Tubulin was detected on the same membrane.

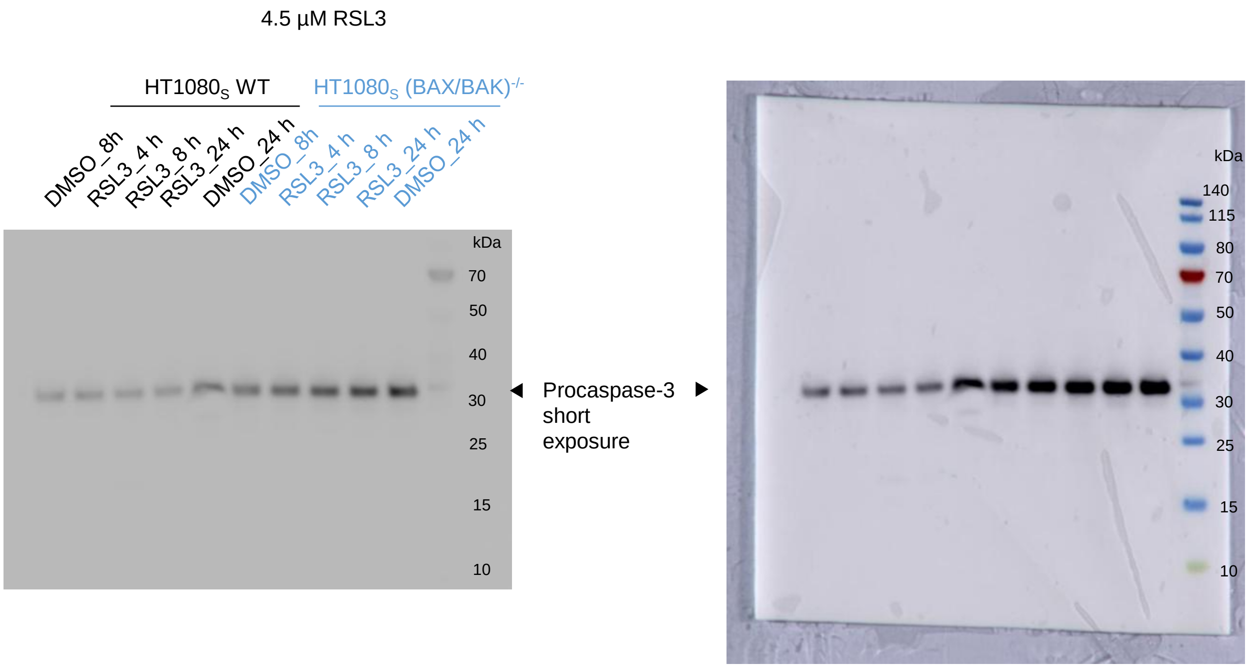

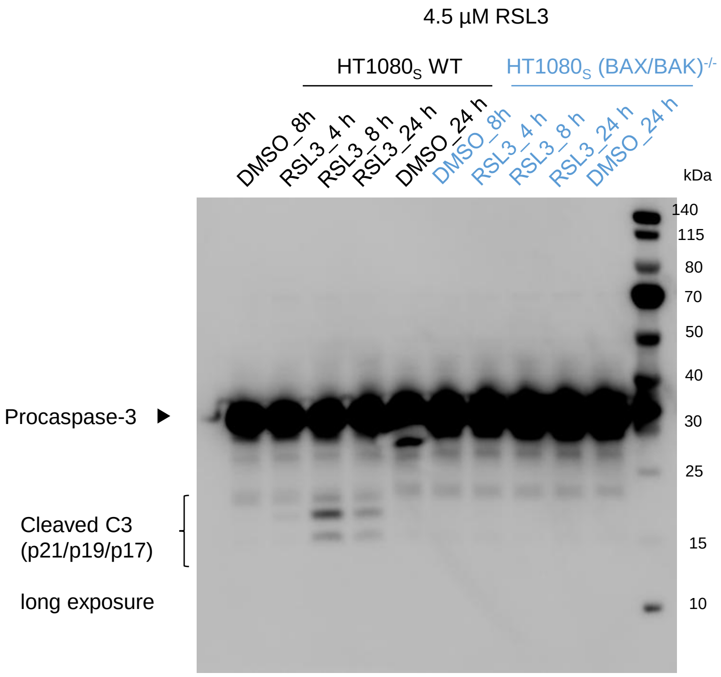

Supplemental Figure 6 E

First caspase-3 was detected, afterwards PARP was detecetd on the same membrane.

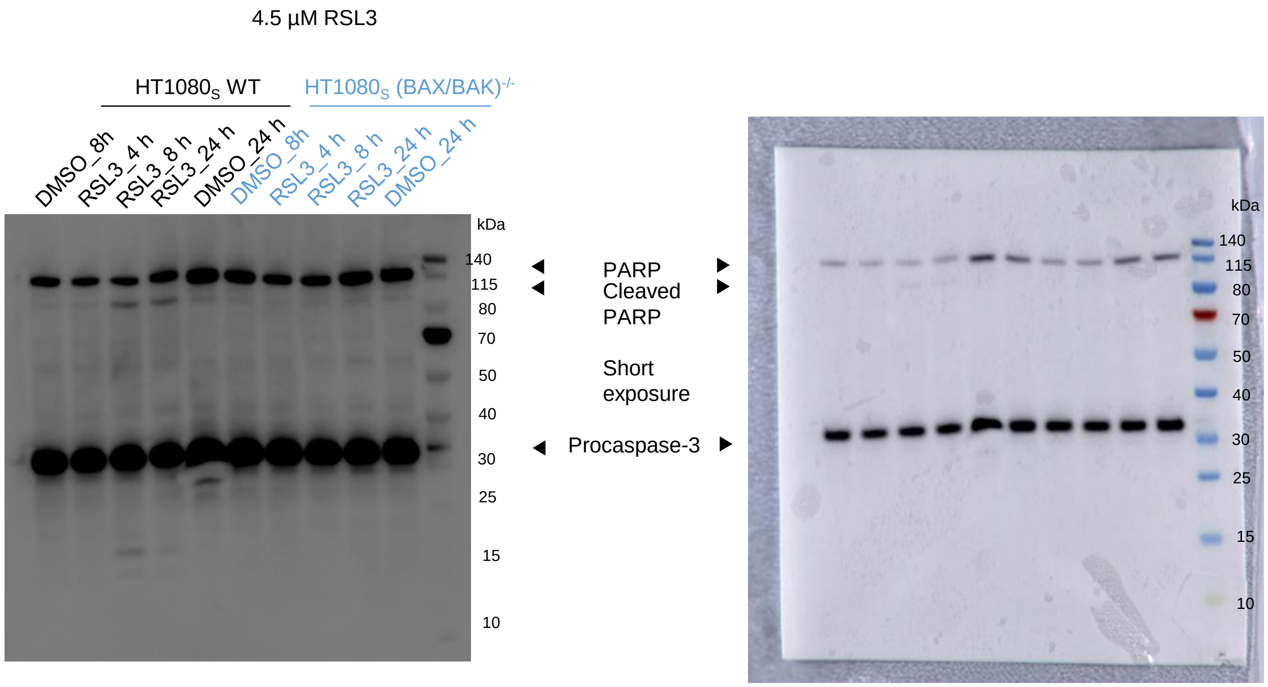

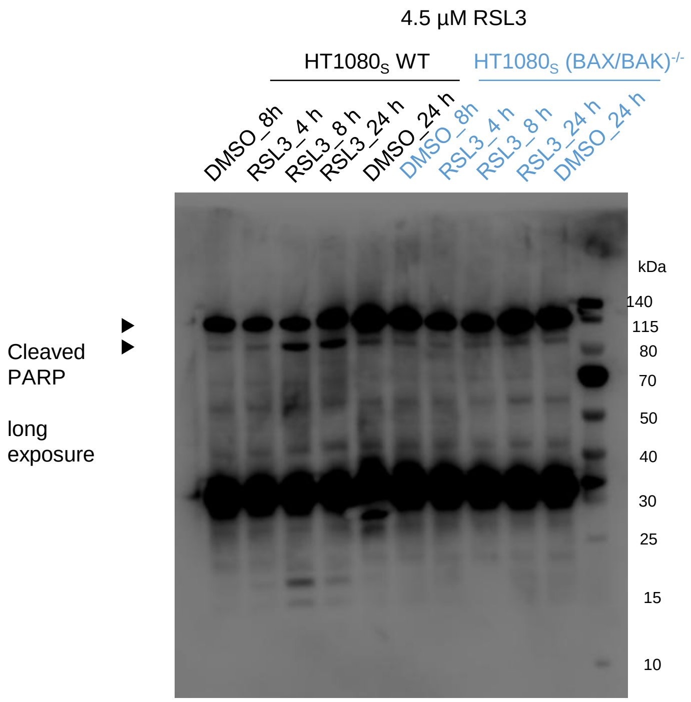

Supplemental Figure 6 E

Lastely, α-Tubulin was detected on the same membrane

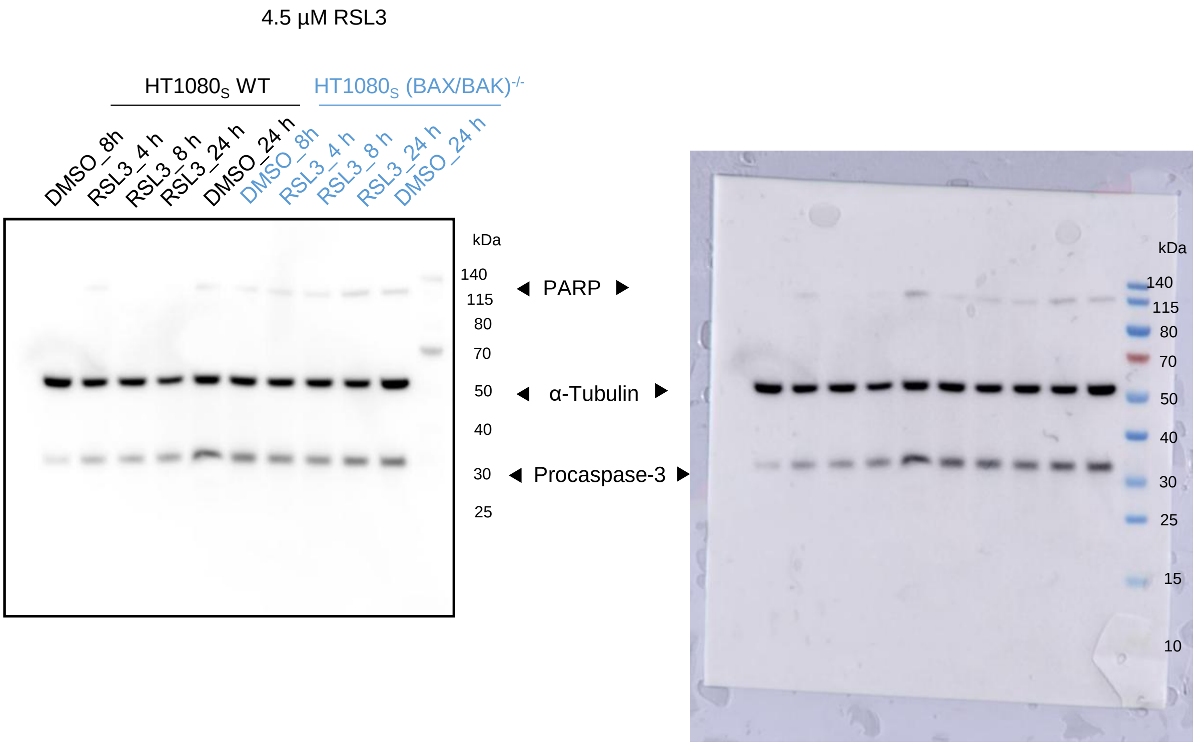

Supplement: Supplementary file 13 — Supplemental Figure 12 - uncropped immunoblots [file 41418_2025_1514_MOESM13_ESM.pdf]
